# Chromatin states reveal functional associations for globally defined transcription start sites in four human cell lines

**DOI:** 10.1186/1471-2164-15-120

**Published:** 2014-03-26

**Authors:** Morten Rye, Geir Kjetil Sandve, Carsten O Daub, Hideya Kawaji, Piero Carninci, Alistair RR Forrest, Finn Drabløs

**Affiliations:** 1 Department of Cancer Research and Molecular Medicine, Norwegian University of Science and Technology, P.O. Box 8905, NO-7491 Trondheim, Norway; 2 St. Olavs Hospital, Postboks 3250, Sluppen 7006, Trondheim; 3 Department of Informatics, University of Oslo, Oslo, Norway; 4 RIKEN Omics Science Center (OSC), 1-7-22 Suehiro-cho, Tsurumi-ku, Yokohama 230-0045, Japan; 5 RIKEN Center for Life Science Technologies, Division of Genomic Technologies, Yokohama, Kanagawa 230-0045, Japan; 6 RIKEN Preventive Medicine and Diagnosis Innovation Program, Wako, Saitama 351-0198, Japan

**Keywords:** Fantom, Encode, Cage, Transcription start sites, Chromatin states, Gene expression

## Abstract

**Background:**

Deciphering the most common modes by which chromatin regulates transcription, and how this is related to cellular status and processes is an important task for improving our understanding of human cellular biology. The FANTOM5 and ENCODE projects represent two independent large scale efforts to map regulatory and transcriptional features to the human genome. Here we investigate chromatin features around a comprehensive set of transcription start sites in four cell lines by integrating data from these two projects.

**Results:**

Transcription start sites can be distinguished by chromatin states defined by specific combinations of both chromatin mark enrichment and the profile shapes of these chromatin marks. The observed patterns can be associated with cellular functions and processes, and they also show association with expression level, location relative to nearby genes, and CpG content. In particular we find a substantial number of repressed inter- and intra-genic transcription start sites enriched for active chromatin marks and Pol II, and these sites are strongly associated with immediate-early response processes and cell signaling. Associations between start sites with similar chromatin patterns are validated by significant correlations in their global expression profiles.

**Conclusions:**

The results confirm the link between chromatin state and cellular function for expressed transcripts, and also indicate that active chromatin states at repressed transcripts may poise transcripts for rapid activation during immune response.

## Background

The transcriptional landscape of human cells is tightly linked to chromatin structure. By modulating chromatin, transcription factors (TFs) and chromatin modifying enzymes decides which transcripts, and the amount of each that are produced by a cell [1,2]. Deciphering the most common modes by which chromatin regulates transcription, and how this is related to cellular status and processes, represents an ongoing endeavor towards our understanding of human cellular biology. However, the diversity of the transcriptional landscapes among different cell types in the human organism, and the complex mechanisms that account for this diversity are just beginning to be understood. Recently two large scale efforts with the goal to map and understand the regulatory and transcriptional landscape of human cells and tissues have been undertaken. Using single molecule Cap Analysis of Gene Expression (CAGE [3]) technology to profile 975 human tissues, cell lines and primary cells, the FANTOM5 consortium has generated a comprehensive map of transcription start sites (TSSs) and their relative expression across the human genome [4] The amount of TSS data produced by this consortium have been condensed into a global set of 184 827 defined Robust clusters of Transcription Start Sites (here abbreviated as RTSSs) throughout the human genome. A robust cluster is defined as groups of TSSs which are in close proximity of each other in the genome, have the same direction of transcription, share a similar global expression pattern across all cells and tissues, and have sufficient support in the number of CAGE sequence tags [4]. The ENCODE project [5] has generated data on a huge amount of features that participate in the regulation of gene expression in human cell lines. Among the several approaches taken by ENCODE to investigate the different aspects of transcript regulation, the mapping of chromatin modifications and transcription factor binding sites in selected human cell lines using ChIP-Seq [6,7] is probably the most comprehensive. To facilitate comparison and utilize the efforts made by both projects, the four cell lines K562, GM12878, HeLa-S3 and HepG2 used by ENCODE where specifically subjected to CAGE in FANTOM5.

One of the important findings in the ENCODE project was the impact on gene expression by different combinations of chromatin modifications at regulatory elements throughout the genome. Chromatin modifications are post-translational chemical modifications, most commonly methylations and acetylations, on the N-terminal tails of the eight histone proteins constituting the nucleosome core. These modifications affect the interaction between the core and the DNA wrapped around it, as well as interactions with chromatin-binding proteins, resulting in configurations of open and closed chromatin [8]. In addition, variants of the histone proteins and modifications to the DNA itself also impact the chromatin configuration. The general distribution of chromatin and other DNA-binding proteins can be analyzed by DNase Hypersensitivity (DNase HS) [9], which can identify regions of open chromatin. Overall features like chromatin modifications, histone variants and open chromatin are referred to as chromatin marks. Studies made by ENCODE and others have shown that different combinations of chromatin marks can separate the chromatin landscape of the genome into states of open and closed chromatin, where closed chromatin generally corresponds to repression of transcription, and open chromatin corresponds to active transcription. Active chromatin can further be separated into additional states, depending on the enrichment of various active chromatin modifications [10-13]. Two examples of such states are found in promoters and enhancers, which affect transcription from proximal and distal genomic locations, respectively. Other studies have shown that the actual shapes of enrichment for individual chromatin marks also differ between genomic locations. However, the functional implications of these differences have been less investigated [14-17].

For studies of chromatin profile shapes, a crucial step is the definition of anchor points throughout the genome, which are used as reference positions for the study of shapes in the neighborhood of the anchor points. TSSs of annotated genes are examples of such anchor points. However, these generally represent a too limited selection of genomic sites for general analysis, considering that a large amount of regulation takes place distal from any annotated gene TSS. Other strategies for anchor point definitions have thus included binding sites for clusters of transcription factors [17] or for specific transcription factors, like the enhancer associated protein p300 [14,18,19]. One challenge with this approach is the lack of directionality of such data. Directionality is important, because individual chromatin shapes have been shown to display asymmetry around anchor points, especially if the anchor points are transcript-producing [17]. Another challenge is the functional heterogeneity of various transcription factors, which can make the anchor points difficult to compare. In contrast to transcription factors, RTSSs as defined in FANTOM5 are both directional and represent a set of genomic sites associated with the same function, that is, activation of transcription. In addition, due to aggregation of data across multiple cell types, a lot of RTSS regions will have zero expression in any individual cell type, since the general FANTOM5 RTSS regions are defined over a comprehensive set of human cells and tissues. This information represents a novel opportunity to investigate chromatin marks genome-wide at locations where transcription is known to be repressed, which could previously be investigated only for TSS positions of annotated genes. TSSs from CAGE were previously used to analyze states for a single chromatin mark (H3K9ac) in few cell lines during FANTOM4 [20].

It has now become well established that the regulatory landscape of the human genome includes much more than the genomic regions surrounding the approximately 22 000 currently well annotated genes. The 184 827 globally defined transcripts from FANTOM5, as well as the mapping of chromatin states and transcription factors in ENCODE are both attempts to map the characteristics and diversity of these transcriptional events, and the mechanism that regulate them. In contrast to most previously known genes, the function of these novel transcripts is mostly unknown. However, several have been shown to correlate with transcriptional outputs of nearby genes [4,19,21-30]. Whether this correlation is due to direct spatial interaction between regulatory elements, co-transcription from the same promoter, assisted recruitment of factors promoting transcription, or establishment of favorable chromatin domains remains to be determined [31], but should nevertheless encourage the association of such non-coding transcripts to nearby genes.

The four cell lines K562, GM12878, HeLa-S3 and HepG2 common to ENCODE and FANTOM5 all include the complete set of 12 chromatin marks mapped by ChIP-Seq in ENCODE. In addition, the 184 827 RTSSs from FANTOM5 defined globally over 975 human tissues, cell lines and primary cells represent an opportunity to investigate chromatin marks at RTSSs repressed in the respective cell-lines, as well as the expressed ones. In this study we have used globally defined RTSSs from FANTOM5 as anchor points, and investigated combinations of enrichment and shape profiles for chromatin marks around these anchor points. Most RTSSs are intra- or inter-genic, rather than being located at or close to currently annotated TSSs. These RTSSs are mostly repressed in the four cell lines studied, however, we also discovered a substantial number of such repressed inter- and intra-genic RTSSs harboring activating chromatin marks and Pol II, indicative of regulatory elements poised for transcription. Using a tool for ontology analysis in genomic regions, we found that these RTSSs were strongly associated with immediate-early responses and cell signaling. Shape profiles for chromatin marks around expressed RTSSs were subjected to a two-level clustering procedure, identifying metaclusters with combinatorial characteristics of enrichment and shape. These metaclusters differed substantially in functional ontology annotations, average RTSS expression, location of RTSSs with respect to nearby genes, and CpG content, indicating that the clusters are biologically relevant. Finally we validated the associations between RTSSs within metaclusters, showing that the global expression levels of corresponding RTSSs are correlated.

This work is part of the FANTOM5 project. Data downloads, genomic tools and co-published manuscripts are summarized at http://fantom.gsc.riken.jp/5/.

## Results

### Globally defined RTSSs are mostly located in intra- and intergenic regions, and repressed in individual cell lines

We defined a set of 179 369 global RTSSs from the 184 827 RTSSs produced by the FANTOM5 consortium, and used this set throughout the rest of the study (Methods). We then mapped the expression profile for these 179 369 RTSSs in each of the four cell lines K562, GM12878, HeLa-S3 and HepG2. To get an overview of the genomic locations of the globally defined RTSSs in each cell line, we divided the 179 369 RTSSs into expressed and repressed RTSSs (Methods), and then further into the following categories: i) *annotated RTSSs* overlapping exactly with RefSeq TSS annotations, ii) *intragenic RTSSs* overlapping with full gene annotations, iii) *intergenic RTSSs* having no overlap with annotated genes, and iv) *proximal RTSSs* located at most 150 bp up- or downstream for annotated gene TSSs (Table 1). The last category was included to account for proximal alternative TSSs for the same gene which often surrounds the annotated TSSs in CAGE data [3,32]. Comparing the fraction of expressed versus repressed RTSSs within the four location categories we are considering, the intergenic and intragenic RTSSs are dominated by repressed RTSSs (2 to 5 fold more repressed than expressed), while the annotated and proximal categories are dominated by expressed RTSSs (1 to 2.4 fold more expressed than repressed). We also observe that more RTSSs are intragenic than intergenic. An overall observed trend is that the RTSS density drops while the cell line specificity of the RTSSs increases as one move away from annotated gene TSSs. In addition, the large number of RTSSs falling into the proximal compared to the annotated category is indicative of substantial alternative TSS usage ±150 bp around annotated TSSs of genes. The number of RTSSs falling into the four categories is quite consistent for all cell lines, and the slight deviation observed for K562 is likely attributable to the lower number of CAGE tags in the K562 library. Our observations fit with previous reports that distal regulatory elements, like enhancers, are generally more cell-type specific than regulatory elements proximal to annotated genes [11,14,33], and the consistent pattern across the cell lines indicates that this is a general feature.

**Table 1:** Cell line specific expressed and repressed RTSSs and their association with genomic regions

		**Total**	**Annotated**	**Proximal**	**Intragenic**	**Intergenic**
K562	Expressed	41 472	6 793	18 134	15 090	8 248
	Repressed	122 575	3 678	17 105	64 677	40 793
GM12878	Expressed	54 475	7 151	19 998	22 949	11 528
	Repressed	101 964	3 084	14 395	52 675	34 894
HeLaS3	Expressed	50 324	7 055	19 892	20 429	10 003
	Repressed	107 756	3 311	14 831	55 866	37 059
HepG2	Expressed	52 862	7 311	21 005	22 305	9 552
	Repressed	105 793	2 973	13 623	54 193	37 977

### Markers for active chromatin show enrichment at both expressed and repressed RTSSs

**Figure 1:**
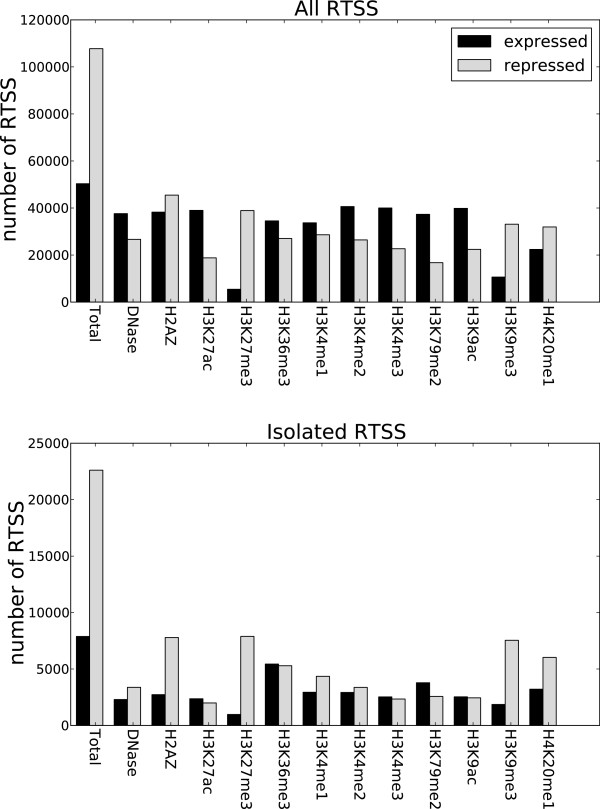
**Active chromatin marks overlap with repressed RTSSs.** The figure shows data for HeLa-S3for all RTSS and isolated RTSS. Data for isolated RTSSs defined as RTSSs separated by at least 2kbp from its nearest neighboring RTSS. The p-values also confirmed highly significant overlaps of active marks with repressed RTSSs, especially for the marks DNase HS, H2A.Z, H3K4me1, H3K4me2, H3K4me3, H3K27ac, H3K9ac and H4K20me1, but less significant overlap with the transcriptional marks H3K36me3 and H3K79me2 [Additional file 1: Table S18]. Plots for the other three cell lines are in [Additional file 1: Figure S1].

The four cell lines used in this study were specifically mapped by CAGE in FANTOM5 for comparison with ENCODE. We could therefore use the 179 369 globally defined RTSSs as anchor points for studying enrichment and profile shapes for the 12 chromatin marks downloaded from ENCODE for each of the four cell lines (Methods). We divided the RTSSs into expressed and repressed, and calculated the number of overlaps for each chromatin mark in a 500 bp extension around each RTSS. Figure 1 shows results from HeLa-S3. Plots for all cell lines are in [Additional file 1: Figure S1]. In general, if results are similar for all cell lines, we display results from only one cell line. As expected, we observed a depletion for the transcriptional repressive marks H3K27me3 and H3K9me3 in expressed RTSSs, but general enrichment for the 10 other marks, which are traditionally regarded as transcription activating marks [34]. More surprisingly, we also observed a considerable enrichment of active marks for the repressed RTSSs. The actual number of repressed RTSSs overlapping with active chromatin marks is comparable to expressed RTSSs. However, the ratio of overlaps compared to the total number of repressed or expressed RTSSs is lower for repressed, since there are more repressed RTSSs than expressed. Many RTSSs are located close to each other in the genome, often separated by less than 100 bp, making it possible that the enrichment observed in repressed RTSSs was due to confounding from neighboring expressed RTSSs. To account for this possibility, we identified RTSSs separated from other RTSSs by at least 2kbp, which resulted in 35 500 isolated RTSSs, and performed the same analysis on these isolated RTSSs. We observed similar, and sometimes increased, enrichment of active marks in the repressed isolated RTSSs compared to the full set of global RTSSs (Figure 1; [Additional file 1: Figure S1]), and p-values calculated for each overlap also confirmed highly significant overlaps (Methods, [Additional file 1: Table S18]). The enrichment was most significant for DNase HS, H2A.Z, H3K4me1, H3K4me2, H3K4me3, H3K27ac, H3K9ac and H4K20me1 and less significant for the transcriptional markers H3K36me3 and H3K79me2. We thus conclude that several active chromatin marks are enriched at both expressed and repressed RTSSs.

### Active chromatin marks at expressed and repressed RTSSs show distinct chromatin profiles, and differ in nucleosome positioning at their TSSs

**Figure 2:**
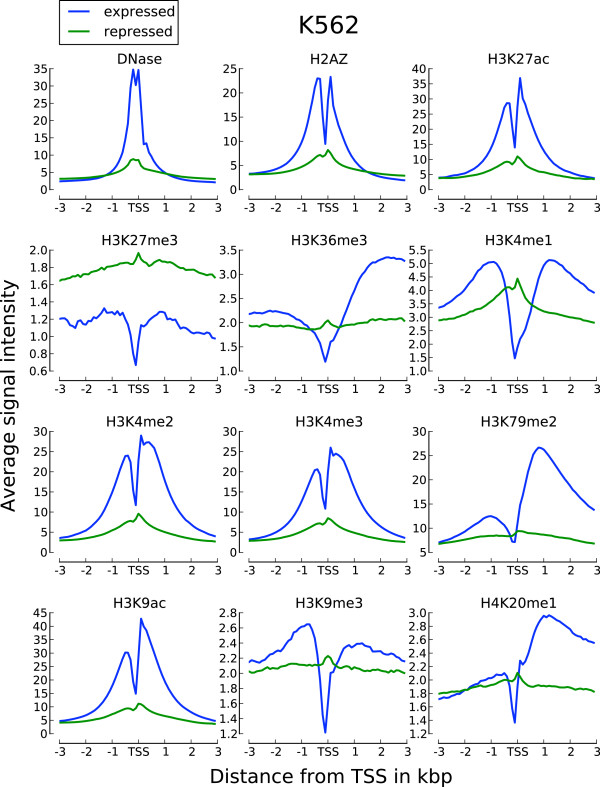
**Expressed and repressed RTSSs display distinct chromatin profiles.** Chromatin profiles are shown around their RTSS center position, here for K562. Profiles for other cell lines are in [Additional file 1: Figure S2].

**Figure 3:**
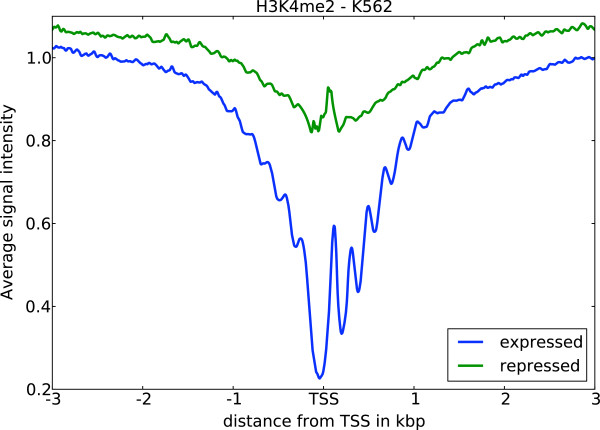
**Nucleosome data at bp resolution reveal different nucleosome positioning.** Nucleosome data at bp resolution reveal different nucleosome positioning around expressed and repressed RTSSs enriched for H3K4me2 in K562. Expressed RTSS nucleosome profiles display highly ordered nucleosome positioning, while this is not evident around repressed RTSSs, except for a well-positioned nucleosome exactly at the RTSS center. In contrast, expressed RTSSs generally display nucleosome depletion at the TSS center.

The enrichment of active chromatin marks on a subset of repressed RTSSs led us to further investigate the shapes of chromatin marks around these RTSSs. We therefore collected and analyzed profiles for all chromatin marks in expressed and repressed RTSSs for the 179 369 globally defined RTSSs in each cell line (Methods). Average profiles (Figure 2; [Additional file 1: Figure S2]) showed that profiles around expressed RTSSs had increased signal for active marks around the RTSS center and transcript body, while repressed RTSSs only had increased signal at the RTSSs center. Expressed RTSSs also display a characteristic dip in the signal exactly at the RTSS center, which corresponds to a nucleosome-free region commonly observed at actively transcribed TSSs [35,36]. In contrast, profiles of active marks around repressed genes lack this characteristic dip, which indicates that these RTSSs retain nucleosome occupancy at the RTSS center. Repressed RTSSs also seem to display a similar symmetric profile around the RTSSs for all active marks, while profiles for expressed genes are either symmetric (DNase HS, H3K4me3, H3K4me2, H2A.Z, H3K27ac, H3K9ac) or show increased signal primarily in the direction of the main transcript (H3K36me3, H3K79me2, H4K20me1). H3K4me1 display slight asymmetry, thus deviating from the other K4 methylation marks in this aspect. Similar profiles were also observed for the isolated RTSSs described above, confirming that the general observations were not due to confounding. Though the repressive marks H3K27me3 and H3K9me3 were more pronounced in repressed RTSSs compared to active marks, we also observed weak enrichment of repressive marks, especially H3K9me3, in expressed RTSSs (Figure 2; [Additional file 1: Figure S2]). To further investigate the nucleosome occupancy around expressed and repressed RTSSs we used nucleosome data from ENCODE, which gives nucleosome occupancy at base pair resolution for the cell lines K562 and GM12878. Nucleosome positioning in K562 around expressed and repressed RTSS enriched for the active chromatin mark H3K4me2 is shown in Figure 3. A clear periodic nucleosome positioning pattern with a dip at TSS is observed for expressed RTSSs, while the repressed RTSSs show no sign of ordered nucleosomes, except for an increased signal exactly at the RTSS center, indicative of a well-positioned nucleosome at this location. The general presence of a nucleosome at the center of repressed RTSSs was also confirmed for all chromatin marks in both cell lines using a lower resolution mapping (Methods). A well-positioned nucleosome at TSS was also a general feature for all repressed RTSSs, not only the ones enriched for active chromatin marks.

Because expressed and repressed RTSSs displayed such distinct profiles characteristics of active chromatin marks, we chose to analyze these two classes of RTSSs separately throughout the rest of our study. We start with the analyses of expressed RTSSs, and continue with the repressed RTSSs afterwards.

### Clustering of chromatin profiles around expressed RTSSs identifies combinatorial subsets of various asymmetric chromatin shapes

**Figure 4:**
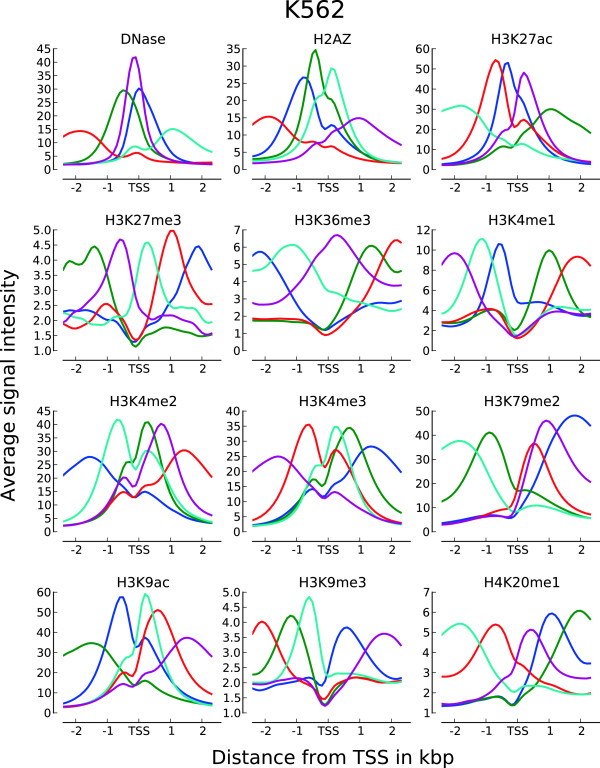
**Average subprofiles for 12 chromatin marks in HeLa-S3.** Average subprofiles for the other three cell lines are in [Additional file 1: Figure S3].

Profiles for a single chromatin mark around expressed genes and active regulatory elements have been shown to display a considerable variation in asymmetric shapes within the same cell type [17]. To identify profile shape variations within each chromatin mark, we used the set of expressed RTSSs as profile anchor points and k-means clustering to identify distinct chromatin profiles over a ±3kbp extension around expressed RTSS center positions. We first performed clustering of RTSS profiles on each chromatin mark in each cell line individually, and continued with a meta-clustering using a combination of correlation coefficients for each RTSS towards each chromatin mark in the respective cell line (Methods). Between 15 000 and 50 000 profiles for active marks and 1000 and 15 000 profiles for repressive marks passed the filtering criteria for inclusion in the first individual clustering. Though k-means clustering is designed to handle a large number of profiles, it requires the number of clusters to be specified prior to clustering. To investigate whether an intuitive prior number of clusters could be identified, we used Principal Component Analysis (PCA) on each set of profiles to see if they displayed discrete profile groupings [Additional file 1: Figure S3]. We could not identify any distinct groups for any mark in any cell line, and the landscape of profile differences in all sets seemed to represent a continuum, rather than discrete groupings. We therefore consistently set the prior number of clusters to 5 for each individual clustering. This number was mainly chosen to give a manageable number of clusters for later interpretation of the meta-clusters. For the first individual clustering we calculated average subprofiles over all RTSS clusters for each chromatin mark in each cell line (Figure 4; [Additional file 1: Figure S4]), resulting in a total of 60 subprofiles (5 clusters and 12 chromatin marks) in each cell line. The same subprofiles were generally observed in all four cell lines, with active chromatin marks displaying more similarity than repressive marks. Several of the identified subprofiles were comparable to profiles identified in previous studies [17], showing various asymmetrical shapes around TSSs. Canonical average profiles for several of the chromatin marks were also visible, for example H3K79me2 and H4K20me1 had several subprofiles with reduced signal upstream of TSSs and gradually increased signal in the transcript direction. In addition we also observed opposite non-canonical subprofiles for H3K79me2 and H4K20me1, with increased signal upstream of TSSs and reduced signal in the transcript direction.

**Figure 5:**
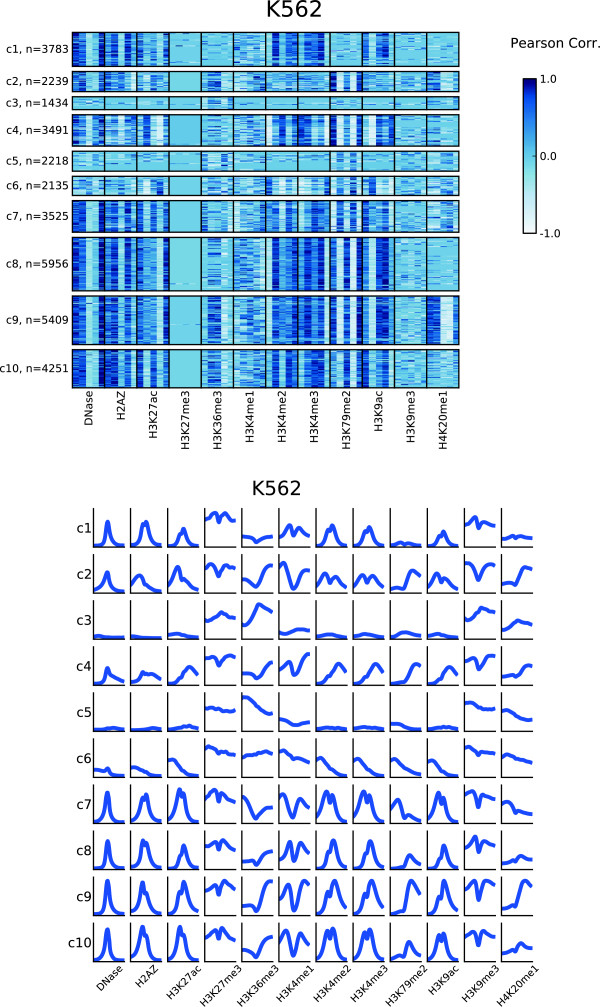
**Meta-clustering identifies combinatorial subprofiles for chromatin marks.** Meta-clustering identifies metaclusters of combinatorial subprofiles for different chromatin marks in K562. A) Heatmap of characteristic subprofiles in each metacluster. Each cell in the heatmap represents the correlation of a RTSS profile with one of five subprofiles identified from the clustering of each chromatin mark. B) Average subprofile over all RTSSs in each metacluster for each chromatin mark in K562. Plots for other cell-lines are in [Additional file 1: Figure S4 and S5].

Having identified subprofiles for individual chromatin marks in each cell line, we next wanted to identify overrepresented combinations of subprofiles using several chromatin marks within each cell line. This was achieved by a meta-clustering approach based on Pearson correlation coefficients between RTSS profiles and subprofiles (Methods). The meta-clustering was applied independently in each cell line. For each chromatin mark, the Pearson correlation coefficient was calculated between chromatin mark profiles for each expressed individual RTSS and the 5 subprofiles for the respective chromatin marks, resulting in 60 correlation coefficients (55 for HepG2, see Methods) calculated for each RTSS. The matrix of all expressed RTSSs and corresponding correlation coefficients was then subjected to k-means clustering with the prior number of clusters set to 10. A heatmap of the cluster results together with subprofiles for all clusters for all chromatin marks are shown in Figure 5 for K562 and in [Additional file 1: Figure S5 and S6] for the other three cell lines. The heatmaps show characteristic subprofiles in all 10 metaclusters, as well as general enrichment of specific marks in each metacluster. For example metaclusters c9 and c2 are exclusively enriched for H4K20me1, c1 is the only cluster depleted for H3K79me2, while c6 is depleted for both H3K4me2 and H3K4me3. Metaclusters c2-10 are all enriched for H3K79me2, however, c5-c7 are dominated by a different H3K79me2 profile than the other 6 metaclusters. Likewise, while general H3K9ac enrichment is found in most metaclusters, c4 displays a dominating H3K9ac profile not characteristic for the other metaclusters. Of all the chromatin marks, the elongation mark H3K79me2 and the two acetylations H3K27ac and H3K9ac seem to contribute most to the subprofile variations between the metaclusters. DNase HS, H2A.Z and H3K4me3 seem to be most stable, showing similar subprofiles in many metaclusters, while profiles for the repressive marks H3K27me3 and H3K9me3, together with H3K4me1, show no specific subprofiles in any metacluster. Most of the trends observed for K562 were also observed in the other cell lines, though some cell type specific differences were also visible. The overall conclusion for the complete clustering approach is that expressed RTSSs can be clustered into distinct groups displaying different enrichment and profile shapes of various chromatin marks.

### Metaclusters of RTSSs differ in functional associations, average expression level, localization with respect to nearby genes and enrichment of CpG-islands

The efforts made by FANTOM5, ENCODE and others have confirmed the huge landscape of transcriptional events existing in addition to the well-known catalogue of protein coding genes. Most of this landscape consists of non-coding transcripts, whose exact functions have yet to be determined. One commonly described property of these non-coding transcripts is their tendency to affect the regulation of nearby genes. To determine the biological relevance of the defined metaclusters, we linked the metaclusters to functional annotations using the publicly available Genomic Regions Enrichment of Annotations Tool (GREAT) [37]. GREAT is a tool that assigns functionality to a set of genomic regions based on nearby genes, and is thus well suited for analyses of RTSS metaclusters with an abundance of intra- and inter-genic elements. In addition, we investigated whether the metaclusters differed with respect to number of associated RTSSs, average RTSS expression level, localization with respect to nearby genes and CpG content.

**Figure 6:**
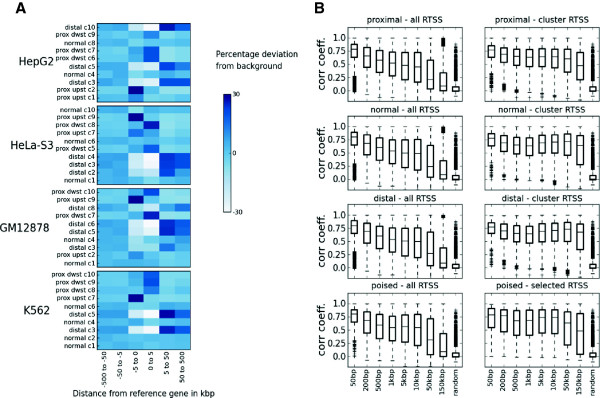
**RTSSs in metaclusters are enriched at different genomic locations. A)** RTSSs in metaclusters are enriched at different genomic locations relative to their nearby genes as calculated by GREAT. The color in each cell represents enrichment (dark blue) or depletion (light blue) of RTSSs in the given genomic interval relative to associated genes. The enrichment is calculated with respect to average enrichment of the full set of RTSSs from all metaclusters. The observed patterns can be divided into normal (resembling the average distribution for the full set of RTSSs), distal, proximal upstream and proximal downstream according to where they are mostly enriched. **B)** Global correlations between RTSSs validate RTSS-to-gene associations identified by GREAT. Both intra-correlations between all globally defined RTSSs in each window (all), and between RTSSs present in the respective clusters (clusters) are calculated for increasing window sizes centered on annotated gene TSSs from GREAT. Correlation values for all proximal, normal, distal and poised cluster sets (as defined in A) have been aggregated for all cell lines. Intra-correlations among RTSS for specific clusters are generally higher compared to intra-correlations between all globally defined RTSSs. The improved correlation was also higher in the more distal windows.

We first observed that the metaclusters in each cell line differed in the number of associated RTSSs, and average RTSS expression level. The number of RTSSs associated with each cluster varied from >11 000 for the largest clusters to 2–3000 for the smallest, while expression levels could be separated into high, intermediate and low [Additional file 1: Figure S7]. Average expression level did correlate with metacluster size. However, the correlation was not absolute in any cell-line. For example the three largest clusters, each containing more than 11 000 RTSSs, were not the ones with the highest average expression in any of their respective cell lines. We also observed differences in RTSS localization preferences with respect to nearby genes for the different clusters (Figure 6a). The most prominent difference was observed between clusters with distal and proximal enrichment of RTSSs relative to genes. Typically 2 or 3 metaclusters in each cell line displayed a distal enrichment, and these clusters generally displayed a low average expression, and contained few RTSSs. The separation of distal and proximal RTSSs was expected, and in concordance with previous reports of different chromatin enrichments in gene proximal and distal elements. More unexpectedly we also observed differences between clusters with RTSSs preferentially enriched upstream or downstream from the TSSs of nearby genes. This property was observed for clusters in all cell lines, involved clusters with most of their RTSSs located proximal to nearby genes, and was mostly observed as a considerable enrichment of RTSSs in the 5 kb region either up- or downstream of their associated gene TSS. Finally, several clusters did not show any specific enrichment of RTSSs in the proximal or distal regions.

To determine functional associations of the different metaclusters, we analyzed functional terms extracted from GREAT for each metacluster. Due to possible confounding of RTSSs located close to each other in the genome, we used two strategies referred to as permissive and conservative for analysis in GREAT (Methods). For both strategies we used the total set of RTSSs in all metaclusters as background data. By doing this, we identify terms significantly over-represented in one metacluster compared to other metaclusters, rather than compared to a general genomic background. A total of 5229 and 3671 genes significantly related to 2114 and 1293 terms were retrieved from GREAT for all metaclusters by this approach, for the permissive and conservative strategy respectively, while no significant genes or terms were retrieved for random selections of RTSSs. Using the permissive strategy, all metaclusters were associated with many, often related, significant terms, while this was only true for a subset of metaclusters in the conservative strategy. Terms associated with individual metaclusters were considerably more different between metaclusters in the same cell line than between metaclusters in different cell lines [Additional file 1: Figure S8]. Metaclusters with similar functional terms between the cell lines also shared individual RTSSs, as well as chromatin configurations in these cell lines, showing that chromatin configurations are reproducible. Both the enrichment of individual chromatin marks and the profile shape of these marks were important for separating metaclusters. For example metacluster c4 in K562 is separated from metacluster c2 by enrichment differences for H3K9ac, H3K36me3 and H3K4me3/2, while the separation from metacluster c8 is mainly due to differences in the profile shapes of H3K9ac, H3K27ac and H3K4me2/3.

**Figure 7:**
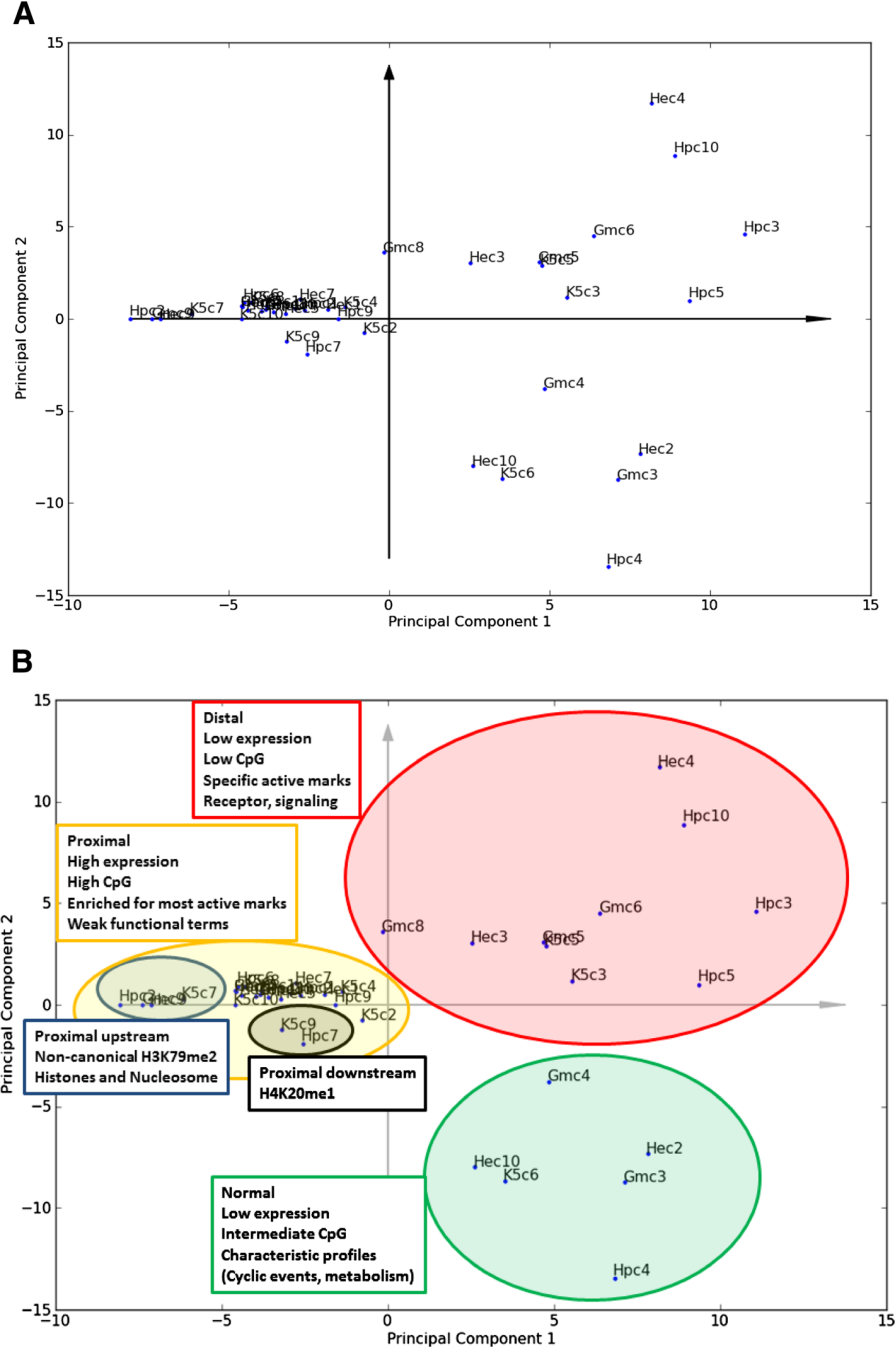
**PCA of metacluster terms from GREAT identifies groups with different functional associations.** The two first components from a Principal Component Analysis (PCA) on the functional terms from GREAT for all clusters in all cell lines. Cell line abbreviations are K5 for K562, Gm for GM12878, He for HeLa-S3 and Hp for HepG2, and c1-c10 are cluster indexes as used previously. **A)** PCA plot for all cluster scores. Clusters with scores in the same quadrant of the PCA plot have related functional terms, and the distance between two clusters corresponds to the degree of similarity. Clusters with scores close to origo (the crossing of the axes arrows) have few functional terms associated with them, while those with scores further away from origo are enriched for several terms. **B)** Interpretations of groupings in the PCA plot, with additional cluster properties also taken into consideration. Three main groups are apparent, with one group (yellow shading) also displaying within-group variation. The terms proximal, normal and distal refers to the localization of RTSS enrichment relative to genes as calculated by GREAT (Figure 6). Interestingly, the more distal clusters with low or intermediate RTSS expression show the strongest functional associations. Displayed PCA plot are from the permissive GREAT analysis. A PCA plot from the conservative analysis are in [Additional file 1: Figure S9].

To investigate metaclusters with corresponding functional associations between cell-lines, we performed PCA on all clusters from each cell-line (40 clusters) using genes from the most significant terms from GREAT for each cluster as model variables. After performing PCA, a plot of the first two principal components revealed several interesting relationships between the metaclusters, and these relations between clusters were mostly conserved between a permissive and conservative strategy employed for functional associations (Figure 7; [Additional file 1: Figure S9]; Methods). The PCA plot indicates that the clusters can be separated into three main groups based on genes from their functional associations. Interestingly, the strongest functional associations are observed among metaclusters enriched with RTSSs distal to their associated genes with scores in the upper right quadrant of the PCA plot. These metaclusters are also characterized by having low expression, low enrichment of CpG islands, and general enrichment for specific active marks. Metaclusters in this group contain terms related to receptors and cell-signaling, with a subgroup of metaclusters (c3 and c5 in K562, c4 in HeLa-S3 and c10 in HepG2) especially enriched for terms related to G-protein coupled receptor (GPCR) signaling. These metaclusters all have a characteristic enrichment of the transcriptional mark H3K36me3, but are depleted for nearly all other marks. Strong functional associations are also observed for the metaclusters with scores in the bottom right quadrant of the PCA plot. These metaclusters are characterized by low levels of transcription, intermediate enrichment of CpG islands, and have a distribution of RTSSs relative to genes resembling the average RTSS-to-gene distribution over all metaclusters. Five of these clusters (c6 in K562, c3 in GM12878, c2 and c10 in HeLa-S3, and c4 in HepG2) share similar chromatin configuration, characterized by H3K9ac, H3K4me3/2, H3K79me2 and to a certain degree H3K27me3 profile shapes that deviate from profile shapes for these chromatin marks in other clusters. Functional terms for these clusters were diverse, but terms related to cell cycle, circadian rhythm and certain metabolic processes like glycolysis were frequent. The third group contains the largest clusters, which generally have lower scores in the left quadrants of the PCA plot. This group is characterized by high and intermediate expression levels, high enrichment of CpG islands, RTSS enrichment proximal to nearby genes, generally high enrichment of many active chromatin marks, and reoccurring functional terms related to various transcriptional activities and processing of RNA and DNA. Metaclusters in this group generally had weaker functional associations, and many metaclusters only returned significant GREAT terms in the permissive setting. The two most prominent subclusters in this group both displayed characteristic chromatin configurations. First, the four clusters with scores leftmost in the PCA plot, with one cluster from each cell line (c7 in K562, c9 in GM12878, c9 in HeLa-S3 and c2 in HepG2), are characterized by the non-canonical upstream enrichment of H3K79me2, are also enriched for RTSSs proximal and upstream of nearby genes, and contain terms related to histone proteins and nucleosome organization. Second, the two metaclusters c9 in K562 and c7 in HepG2 located close together in the PCA plot are the only metaclusters particularly enriched for H4K20me1.

The annotation terms identified by GREAT imply a functional association between RTSSs and their nearby annotated genes. To validate that sensible RTSS-to-gene interactions are represented in these associations, we used the global expression profiles over all 975 FANTOM5 samples and calculated intra-correlations between RTSSs in windows of increasing size, anchored on annotated TSSs of genes associated with RTSSs through GREAT (Figure 6b, Methods). For each window and each cluster, we compared intra-correlation in expression profiles between RTSSs present in each cluster to the correlation observed when all globally defined RTSSs within the window were considered. We generally observed a higher correlation between RTSSs within clusters than within all globally defined RTSSs, especially in windows representing the distal RTSSs. The high correlations observed in all calculations indicate that co-expression of nearby genes within clusters is substantial. Overall the results supports that many of the RTSS-to-gene associations identified by GREAT are sensible, and validate that strategies such as those applied by GREAT to attach possible functions to non-coding transcripts that currently lack functional annotations are feasible.

In general, all observations described above show that the identified metaclusters differ in several properties and associated functions, that properties, functions and chromatin states are related, and that these relations are reproducible across cell lines. All these results when taken together show that the subclusters that were identified by the different chromatin configurations through the metaclustering approach are biologically relevant.

### Repressed RTSSs enriched for active marks are linked to immune response by gene ontology terms, and contain additional enrichment of polymerase II

**Figure 8:**
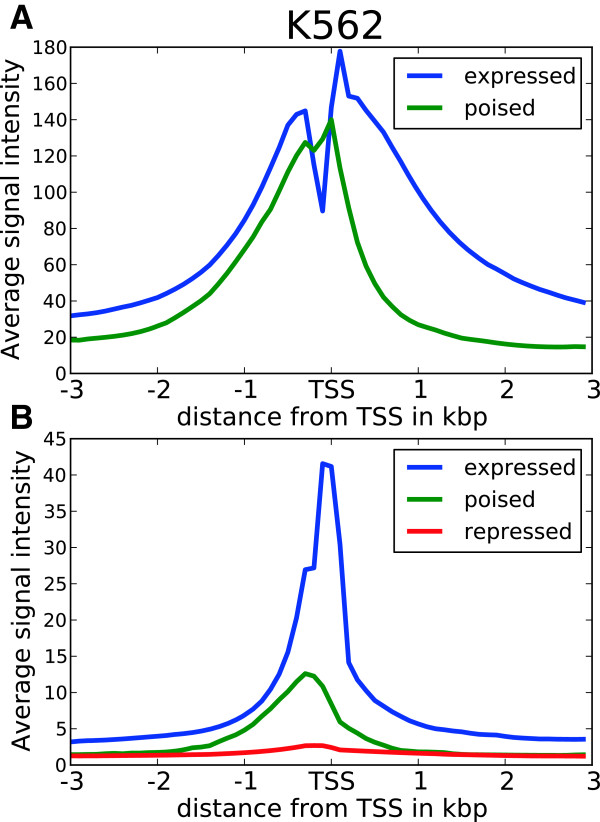
**Poised RTSSs enriched for active chromatin also have enrichment of Pol II.** A selection of 6184 characteristic poised RTSSs enriched for active chromatin marks also have additional enrichment of Pol II in K562. Plots for the other cell lines are in [Additional file 1: Figure S11 and S12]. **A)** Average pooled profile of all active chromatin marks for expressed RTSSs and the selected subset of 6184 poised RTSSs. **B)** Average Pol II profile around expressed RTSSs, the subset of selected poised RTSSs and all repressed RTSSs.

As described above, we found considerable enrichment of active chromatin marks at repressed RTSSs throughout our set of 179 369 globally defined RTSSs (Figure 2). Because of the general profile similarity of all active marks around repressed RTSSs, we pooled the profiles of all active marks around each repressed RTSS, and identified a robust subset of RTSSs with a general active profile for each cell line (Figure 8a; [Additional file 1: Table S10 and Figure S11]; Methods). This filtering procedure resulted in subsets of 6184 RTSSs for K562, 3813 for GM12878, 4345 for HeLa-S3 and 4303 for HepG2, which constitutes between 4% and 6% of all repressed RTSSs with significant signal in at least one chromatin mark. To separate the selected RTSSs from the generally repressed RTSSs, we from now on refer to the former as poised RTSSs. Between 15% and 30% of the poised RTSSs overlapped between the cell lines. Of the 13 693 poised RTSSs selected over all four cell lines, only 253 (2%) were present in all cell lines while 10 103 (74%) were present in only one cell line. The poised RTSSs reflect the genomic distribution of repressed RTSSs in general, in that less than 20% are proximal to annotated genes, and over 80% are intra- or intergenic, the latter also being highly cell line specific.

Genes responding rapidly to environmental stimuli, for example in immune response, have been shown in several studies to display only low levels of transcription, but with marks characteristic of an active chromatin state, also when the cell is unstimulated [38-41]. These genes were defined as being in a poised state, where the active chromatin poises the gene for rapid activation in response to external stimuli. Similar poised states were also recently shown to exist for enhancers [42]. An additional common feature described in these studies was the additional enrichment of polymerase II (Pol II) in the poised genes and enhancers, where the initiating form of Pol II, but not the elongating form, was generally observed in the poised regions. The existence of initiating Pol II was shown to transform to the elongating form rapidly in response to stimuli [43]. As the region changed from poised to active, more Pol II was also shown to be recruited to the region. To investigate whether our selected RTSSs displayed characteristics of such poised regions, we downloaded data on Pol II in all four cell lines from ENCODE (Methods), and investigated the Pol II enrichment in our poised RTSSs. Indeed, we found that our poised RTSSs showed enrichment of Pol II in all four cell lines (Figure 8b; [Additional file 1: Figure S12]). The enrichment was less than for expressed RTSSs, but considerably higher than the general average over all repressed RTSSs.

We also tried to investigate whether our selected regions would respond to external stimuli. For this analysis, we could only find one relevant dataset from ENCODE. The data was for Pol II enrichment in the K562 cell line after stimulation with interferon alpha (IFNα) and gamma (IFNγ). We observed similar levels of Pol II for our selected RTSSs relative to Pol II levels for expressed RTSSs before and after stimulation of both IFNα and IFNγ (details in S13, [Additional file 1: Figure S14 and S15]). This was in contrast to the previous studies which reported a general increase in Pol II levels after stimulation [43].

**Figure 9:**
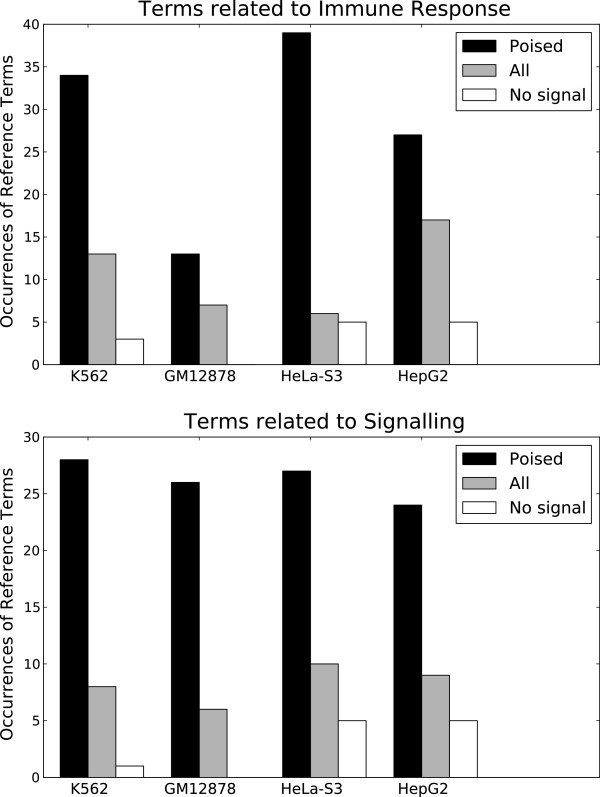
**Selected RTSSs enriched for active chromatin are enriched for specific terms.** Selected subsets of RTSSs enriched for active chromatin marks are also enriched for terms related to Immune Response and Signaling in all four cell-lines. In the legend *Poised* are the selected subsets of poised RTSSs, *All* are sets of RTSSs with equal sizes as the selected ones, but drawn randomly from the set of all repressed RTSSs in each respective cell line, while *No signal* are drawn randomly from the set of repressed RTSSs with no significant enrichment of active chromatin marks. The sets drawn from all repressed marks are also somewhat enriched for Immune Response and Signaling terms, however this association is considerably stronger for the selected RTSSs subsets.

To investigate possible functions of the selected poised RTSSs, we again used GREAT for GO annotation (Methods). We found that our selected RTSSs were highly enriched for terms related to Immune Response and Signaling in all four cell lines, compared to randomly selected sets of RTSSs (Figure 9). So even though the selected RTSSs only partly overlap between the cell lines, they seem to be related to similar functions in all four cell lines. In addition to functional terms, GREAT also returned lists of all the genes associated with the input genomic regions (RTSSs). In total the 13 693 poised RTSSs were associated with 1148 unique genes by GREAT (537 in K562, 380 in GM12878, 592 in HeLa-S3 and 293 in HepG2). As expected from the functional terms, the gene lists are dominated by genes typically related to early response, signaling and the immune-related processes, like *FOS*, *JUN*, *BCL3*, *EGR*-family, *TNF*-family, *NFkB*-family, MAP kinases, interleukins and interferons. When comparing our 1148 genes to a compiled set of 67 early response genes from a study in mice [44], we found exact matches for 44 of the 67 genes, while 15 of the remaining 23 matched closely related genes. As examples of the latter we found *SAA1* but not *SAA3*, *ARHGEF1* but not *ARHGEF3*, *NOS3* but not *NOS2* and *IRF1*, *2*, *4*, *5*, *6* and *9* but not *IRF7*. The cell type specificity of affected genes is comparable to the cell type specificity of the selected RTSSs, with only 46 (4%) genes affected in all cell lines, and 705 (61%) affected in only one cell line. Similar to ubiquitously expressed genes in general, the CpG content in promoters of the 46 genes affected in all cell lines was significantly higher (p < 0.05 by Monte Carlo sampling, Methods) than for promoters in the other 1102 genes. Affected genes are both repressed and (already) expressed in their respective cell-lines, in proportions similar to expressed and repressed genes in general. When considering only the repressed genes in the gene list, they showed higher signals for active chromatin marks compared to generally repressed genes, indicating that they may exist in a somewhat poised state as well [Additional file 1: Figure S16]. However, these signals were considerably less than for our selected poised RTSSs. Overall, genes related to immune response and cell signaling must be able to react rapidly in response to environmental cues, and it thus makes sense that response elements affecting such genes, here represented by nearby RTSSs, exist in a poised state with active chromatin marks. Although our set of selected RTSSs did not respond to stimulation by IFNα or IFNγ, the functional associations from GREAT, the strong association of the corresponding genes with signaling, immune and early response genes, and the enrichment of Pol II and active chromatin marks at the selected RTSSs, corroborates the indication that these RTSSs represents poised, mostly intra- and intergenic elements ready to be activated rapidly as a response to environmental cues. Finally, as for the expressed RTSSs, we observed increased correlation between global expression profiles within neighboring poised RTSSs compared to neighboring RTSSs in general (Figure 6b).

## Discussion

Several studies have now shown that the transcriptional landscape of human cells is far more diverse than previously anticipated [5,45,46]. In addition to the well-known protein coding transcripts, an abundance of intra- and intergenic non-coding transcripts are also produced, whose functions have yet to be determined. Based on results from previously published studies, we assume that one role of these non-coding transcripts is to affect the expression of genes in their neighborhood, and have used GREAT to assign functional relationships to these non-coding transcripts through their association with nearby genes. Moreover we have assumed that transcripts sharing similar patterns of chromatin enrichment and profile shape are associated with similar functions, and have thus grouped the transcripts into distinct clusters based on chromatin features. This strategy has revealed strong non-overlapping functional associations for the different clusters, many of which are reproducible across the four studied cell lines. Some of the identified clusters also display chromatin configurations which, to our knowledge, are yet uncharacterized. The two most prominent of these are clusters with a non-canonical H3K79me2 profile associated with cell division, nucleosome assembly and histone proteins found in all four cell lines, and clusters with sole H3K36me3 enrichment related to G-protein coupled receptor signaling found in three of the cell lines.

### Intra- and intergenic RTSSs have correlated expression with nearby genes

In this study we have assumed that an important function of regulatory elements, including RTSSs producing non-coding transcripts, is to affect the transcription of nearby genes. The RTSSs is a subset of the general collection of regulatory elements available to a cell, which also includes enhancers and insulators, many of which do not produce their own transcripts. There are individual examples of situations where regulatory elements affect flanking genes [47-50], and where the regulatory element and the affected gene are separated by several unaffected genes [51-53]. However, it less known how common these modes of regulation are on a global scale. Some studies have found the association of regulatory elements to flanking genes to be substantial [11,54,55], while other studies using 5C technology [56] to identify spatial genomic interactions have concluded that the association between a distal regulatory element and its closest gene is less common [57]. Studies of spatial genomic interactions have also revealed that a single regulatory element may affect several genes, and a gene may be affected by several regulatory elements, complicating the picture further [58]. However, while spatial interaction is necessary for some regulatory elements, like enhancers, to execute their function, transcript-producing regulatory elements do not necessarily need to interact directly with the genes to affect their transcription. GREAT allows associations with both upstream and downstream genes at the same time, but not beyond the closest gene or a genomic distance limit. Considering GREAT’s dependence on closest gene associations, the strong functional relations observed in this study may seem somewhat surprising. We acknowledge that several of the individual RTSS-to-gene associations identified by GREAT may be false positives. However, we see several reasons why the functional analysis still might work. i) The analysis in GREAT is based on statistical overrepresentation, which makes it robust against low levels of misclassification. ii) Several of the clusters are enriched for RTSSs that are proximal to, and often coincide with, their nearest gene. These RTSS-to-gene associations are thus very likely to be true. iii) Genes with similar function may have a tendency to be located in the same genomic region [59,60]. So, even if a specific RTSS-to-gene association is wrong, the RTSS may still be affiliated with a gene with similar function, resulting in a correct functional association. iv) Validation of RTSS-to-gene associations using global expression profiles shows that the correlations for RTSSs within the same clusters are higher than for closely located RTSSs in general, and that this improved correlation is most visible for distal RTSSs. This indicates that the RTSS-to-gene associations used by GREAT are more likely to be correct than a random association between two RTSSs within the same genomic region. We have not validated individual RTSS-to-gene associations in this study. However, several associations are interesting candidates for further investigation. Overall we have shown that clustering RTSSs based on chromatin configuration, and using GREAT for ontology annotation of each cluster, has produced functional annotations for these clusters that seem to be reasonable and are reproducible across cell lines.

### Inter- and intragenic RTSSs enriched for active chromatin marks and Pol II are poised for activation

The investigation of average chromatin profiles around repressed and expressed RTSSs has revealed a subtle relationship between open and closed chromatin, and between transcript repression and expression. Especially our analyses of a selected subset of poised RTSSs that are substantially enriched for active chromatin marks, but with zero expression levels, shows that chromatin state is not always directly correlated with active transcription. In addition we also observe slight enrichment of repressive marks, especially H3K9me3, at expressed RTSSs. Possible reasons for H3K9me3 enrichment in gene bodies have been described previously [61], but their potential effect on TSSs has to our knowledge not been studied. Our poised RTSSs were selected by quite conservative criteria, but should still make up a representative subset for this category of RTSSs. As evidence for this, an alternative selection procedure resulted in sets of poised RTSSs which were highly overlapping with the sets used for these analyses. Poised regulatory elements reside generally in regions of open chromatin, and are used by the cell to respond rapidly to environmental cues. Because they reside in open chromatin, their function can be initiated with at most a limited degree of chromatin remodeling, and often also without any *de novo* production of transcription factors [44], and this ensures rapid activation. We observed characteristics for our RTSSs which indicate that they represent such poised regulatory elements. First we observed substantial enrichment of Pol II at the RTSSs, which is a typical hallmark for poised regulatory elements. This enrichment was observed independently of the selection procedure. Previous reports have discussed the role of stalled Pol II at poised regulatory elements, and it has been suggested that one role of these elements is to load Pol II onto the gene promoter through the activation of long-range spatial interactions [62,63]. In our analysis, since the RTSSs actually represent transcription events, we find it more likely that Pol II initiates transcription at the respective RTSSs, though we cannot exclude that at least some RTSSs also function through other mechanisms. Second, the selected RTSSs are located in regions that are also occupied by genes associated with immune responses, cell signaling and general immediate cell responses, all of which are activated rapidly in response to environmental cues. Several of the poised RTSSs are located proximal to, or coincide with, the actual genes, creating quite robust RTSS-to-gene associations. For the distal RTSSs we again observed a higher global correlation of expression between the poised RTSSs in the genomic region than for general RTSSs in the same region, confirming that many of the RTSS-to-gene associations are also likely to be relevant. Third, common immediate response genes like *FOS* and *JUN* were associated with poised RTSSs in all cell lines, and we observed a higher CpG content in genes associated with all cell types than genes associated with three or less cell types, in accordance with other data on subsets of immediate-early response genes [44].

### The predefined number of clusters reveals functional features despite lack of fine-structure in data

In our study we set the predefined number of clusters for the k-means clustering to 5 for clustering of individual chromatin marks, and 10 for the combination of marks. Other studies have identified higher number of profiles, both for each chromatin mark and for combinations of marks [10,17], where the final number of states has been determined through various optimizations of a clustering procedure. Visual inspection of score plots from PCA revealed no obvious separation of groups of profiles for any chromatin marks, leaving no suggestion for an initial estimation of the number of clusters. However, the variation in RTSS profile shapes should still warrant that a separation into groups is meaningful. Thus the number of clusters was chosen to be suitable for keeping the number of states equal for all chromatin marks and cell lines, and at a level convenient for interpretation. The numbers chosen turned out to be sufficient for producing relevant functional associations, and thus this works as a proof of principle. We anticipate that more sophisticated ways of selecting clusters will probably produce stronger and more detailed functional associations than the ones observed in this study.

The regulatory landscape governing transcription in different cell types is highly complex. However, it is also predictable, in that the same cell type responds similarly every time it is subjected to the same environmental cue, and coordinated, in that several transcriptional elements respond in the same manner to stimulation. Genome wide mapping of various features, whether it is expression level measurements, TSS activity level, chromatin configuration, DNase HS or transcription factor binding and activity, all leave traces of this coordinated action. Thus, it is an important challenge to integrate such data and determine at what level it is meaningful to look for general patterns that are robust and predictable on a global scale, to investigate what these patterns mean in terms of function and phenotype, and what the main components that govern these patterns are. In this study we have used combinations of chromatin marks around a global set of experimentally defined TSSs, and identified subsets of TSSs with similar chromatin configuration, several of which have functional associations. Hopefully this and related strategies, together with integration of even more genome wide features, will continue to reveal patterns of ubiquitous and cell type specific gene regulation, expression and function.

## Conclusions

We have integrated chromatin data from the ENCODE consortium with the robust set of globally defined TSSs from FANTOM5 to investigate how chromatin features can be used to distinguish TSSs with different properties in four cell lines analyzed by both consortia. We find that most TSSs are repressed in the cell lines studied here, however, a substantial number of the repressed TSSs are enriched with active chromatin marks. These TSSs are strongly associated with immediate-early response processes and cell signaling. Expressed TSSs can be clustered into subsets based on combinations of both enrichment and profile shape of individual chromatin marks. We identified three main groups of clusters which differ in average TSS expression, CpG island enrichment, TSS location with respect to nearby genes and functional GO terms. Interestingly, groups with clusters enriched for TSSs distal to nearby genes show the strongest functional associations. Finally we show that nearby TSSs with similar chromatin configuration show better correlation in global expression profiles than nearby TSSs in general, thus validating the link between chromatin states and cellular function.

## Methods

### Data sources

ChIP-Seq mapped tag libraries, and enrichment regions for the 10 histone modifications H3K4me1, H3K4me2, H3K4me3, H3K27me3, H3K36me3, H3K9me3, H3K27ac, H3K9ac, H3K79me2, H4K20me1, histone variant H2A.Z (Broad Histone, Broad Institute), DNase hypersensitivity (DNase HS, Duke DNaseI HS, Duke University) and Pol II (SYDH TFBS, Stanford/Yale/USC/Harvard) were downloaded from ENCODE for the four cell lines K562, GM12878, HeLa-S3 and HepG2 [64]. In addition, 4 ChIP-Seq datasets of Pol II after stimulation with IFNα and IFNγ measured after 6 and 30 hours were also downloaded from ENCODE (SYDH TFBS, Stanford/Yale/USC/Harvard). Nucleosome position sequencing data for the cell lines K562 and GM12878 were downloaded as *bigWig* files from ENCODE (Stanf Nucleosome, Stanford/BYU). For RTSS from FANTOM5, we started with a preliminary global CAGE RTSS dataset of 180 338 robust RTSS (this set was later expanded to 184 827 for the FANTOM5 main paper [4], the additional RTSS were not included in this study), in addition to cell-type specific expression of these clusters in the four selected cell lines. Three RTSS expression replicates were pooled into a single expression profile in each cell line. RTSS overlapping with unmappable regions from ENCODE [64] (mapability, exludable regions from Duke University and Stanford) were removed prior to analysis, reducing the number of global RTSS to 179 369. In addition, we encountered unexpected profiles for histone modification H3K27me3 in HepG2. These profiles were not confirmed by a second H3K27me3 dataset (UW Histone, University of Washington)) from ENCODE. We thus decided to discard this dataset from the analysis. An overview of all datasets used in the analysis are listed in [Additional file 1: Table S17].

### Overlap of chromatin marks with expressed/repressed RTSSs

To define expressed RTSS, we used a mapped tag threshold of 5 for the cell lines GM12878, HeLaS3 and HepG2 and 3 for K562. The reason for the lower threshold in K562 was that the CAGE tag library for K562 contained a lower total number of tags (10.7 m) than the other three cell lines (30.2 m, 26.5 m and 33.1 m respectively). Only RTSSs regions with zero tag count were defined as repressed. RefSeq genes (UCSC Genome Browser 18.10.2011) [65,66] were used for gene annotations. RTSSs in the category ±150 bp proximal to annotated RefSeq TSS were required to have the same strand directionality as the annotated TSS. Overlaps between RTSSs and chromatin marks were calculated for each mark individually using downloaded enrichment peak-profiles from ENCODE (filename extension *.broadPeak* for histone modifications and histone variant H2A.Z and *.narrowPeak* for DNase HS). An overlap between an enrichment peak and a RTSS was identified if the enrichment profile overlapped the RTSS plus a 500 bp extension from each end of the RTSS region. The extension was used because some chromatin marks associate with RTSSs up- or downstream, rather than at the exact position of the RTSS. Isolated RTSSs were defined as RTSSs with a genomic distance of at least 2kbp from any other RTSSs. The p-value for each overlap was calculated by the Genomic Hyperbrowser [67] using a Monte Carlo scheme with 100 permutations [Additional file 1: Table S18]. Details of the calculations can also be found at [68].

### Processing of chromatin marks around RTSSs

Profiles around each of the 179 369 globally defined RTSSs in each cell line for all chromatin marks were calculated from ChIP-Seq mapped sequence read libraries downloaded from ENCODE. Replicates for each chromatin mark were pooled. RTSS center positions were used as genomic anchor points for profile regions spanning 3 kbp in both directions from the anchor point. Because the average sequence read length was estimated to be around 200 bp (ENCODE, Broad Histone, Broad Institute), start positions for the mapped reads were shifted by +100 bp for reads mapped to the positive strand, and -100 bp for reads mapped to the negative strand. Each profile was then calculated by summing all start positions in 100 bp intervals up and downstream of the anchor point, extending 3kbp in each direction. To limit the impact of noise, only RTSSs overlapping with ENCODE-defined significantly enriched regions for each chromatin mark were used to calculate the average profiles. Nucleosome data for K562 and GM12878 were downloaded as *bigWig* files from ENCODE, and profiles were calculated by summing values in 100 bp intervals ±3kbp around RTSSs as described for the chromatin marks.

### Clustering of individual chromatin marks within each cell line

Clustering was performed for each chromatin mark in each cell line individually using k-means clustering with number of clusters set to 5. We chose this number both because it generally produced subprofiles that were clearly distinct in shape, and to avoid too much combinatorial variation for the subsequent meta-clustering (see below). For clustering we used profiles for all expressed RTSSs in each cell line. The profiles were calculated using the RTSS center position as anchor point, and averaging sequence read intensities in 100 bp windows extending 3kbp both up and downstream. RTSSs with profiles containing less than 100 reads for a chromatin mark were filtered out for the clustering of this mark. Applying this filter resulted in between 15 000 and 50 000 profiles clustered for each active mark, and between 1000 and 15 000 profiles for each repressive mark. All profiles selected for clustering were smoothed prior to clustering using Gaussian convolution with window size of 7 bins. Removing edge effects caused by the smoothing reduced the number of measuring points in each profile from 60 to 48. Clustering was performed using the *kcluster* function in the Python *Bio.Cluster* package. We used *Pearson Correlation* as distance measure rather than *Euclidian Distance* to emphasize profile shape rather than intensity differences, and also to reduce the effect of normalization. We did repeated analyses with number of passes, *npass,* set to 10, and found that this number made each clustering fairly reproducible as evaluated by visual inspection of the resulting profiles. We thus chose 200 passes in the final clustering of each chromatin mark, which should be sufficient to produce robust cluster profiles. All other parameters were set to default. To investigate the effect of confounding, we also selected profiles from isolated expressed RTSSs and clustered them separately. Clustered profiles using only the isolated clusters were generally comparable to cluster profiles from the full sets of expressed RTSSs. We thus used results from the full set clustering for further analysis.

### Meta-clustering of correlation coefficients for multiple chromatin marks within each cell line

The initial clustering produced 5 average subprofiles for each of the 12 chromatin marks in each cell-line. For each expressed RTSS, a Pearson correlation coefficient was calculated between each of the RTSS chromatin profiles and the five subprofiles for this chromatin profile. This resulted in 60 correlation coefficients for each of the expressed RTSSs (55 for HepG2, because H3K27me3 was excluded from this cell line). Correlation coefficients where the total chromatin signal was below the predefined threshold of 100 where set to zero. The matrix of expressed RTSSs and chromatin profiles was then subjected to k-means clustering, with number of clusters set to 10 and number of passes to 1000. Other cluster parameters were the same as previously defined. The clustering was performed independently in each cell line.

### Robust subset of repressed RTSSs with active marks

In each cell line profiles for all active marks around each repressed RTSS were pooled to create a general active profile for each RTSS. A RTSS was selected for the robust subset if it i) overlapped with a peak-region of significant enrichment for any chromatin mark, ii) the total signal for the pooled profile was above a threshold set to 1000 reads, iii) the correlation of the RTSS profile to the average pooled repressed profile was above 0.5, and iv) the correlation of the RTSS profile to the average repressed profile was significantly better (p-value ≤ 0.05) than the correlation to the average expressed profile. For the last criteria we implemented a statistical test for comparing dependent correlations [69]. A total number of 6184 RTSSs for K562, 3813 for GM12878, 4345 for HeLa-S3 and 4303 for HepG2 passed these filtering criteria [Additional file 1: Table S9]. To confirm the robustness of the selected subsets, we also applied a second procedure to select repressed RTSSs with active marks. Instead of pooling the samples, we now used the five criteria described above on each chromatin mark individually, using a threshold of 100 (instead of 1000) on each individual mark. Then only repressed RTSSs which passed all criteria in at least three active marks were selected. This resulted in slightly fewer RTSSs for each cell line compared to the other selection procedure. Between 60% and 80% of the RTSSs selected by the second procedure were also selected by the first procedure. This overlap is high, considering that the selected RTSSs only constitute around 5% of the total number of repressed RTSSs enriched for any chromatin mark. We thus conclude that the selected subsets represent a robust selection of repressed RTSSs with active marks in each cell line.

### Gene ontology enrichment analysis by GREAT

RTSS regions for each of the 40 metaclusters (10 in each cell-line) were individually submitted to the Genomic Regions Enrichment of Annotations Tool (GREAT) [37] using default parameters and the full set of expressed RTSSs from each cluster’s corresponding cell line as background. Terms and associated genes for each term were extracted for the most relevant categories, which we determined to be *Molecular Function, Biological Process, PANTHER Pathway, Pathway Commons, BioCyc Pathway* and *MSigDB Pathway.* Only terms displayed by GREAT were included in the analysis. In default mode, GREAT only displays the top 20 terms for each category which pass two statistical tests (p-value < = 0.05): A binomial test which accounts for over-representation in genomic regions, and a hypergeometric test which accounts for over-representation in functionally associated gene sets. In addition, the region fold enrichment must be larger than 2 for a term to be reported. For each cluster, localization enrichment with respect to associated genes was retrieved from *Region-Gene Association Graphs (Binned by orientation and distance to TSS)* displayed by GREAT*.* An issue with the initial analysis using RTSS locations in each cluster was the possibility of confounding of nearby RTSSs. RTSSs located less than a few hundred bp apart may have a confounded chromatin signature, which may bias the significance of some terms in GREAT. To deal with this issue, we applied two strategies for CAGE analysis. In the permissive strategy we used all RTSSs in each metacluster as input, thus allowing more weight to be put on regions where many RTSSs are located close together, while in the conservative strategy we merged all RTSSs within a 100 bp window surrounding anchor RTSSs. The RTSSs used as anchors for merging were the ones having the highest proximity to other RTSSs. The matrix used for PCA on GREAT terms was constructed by first listing all genes associated with significant terms for all metaclusters. Then, for each gene and each cluster, a value of 1 was assigned if significant terms for this cluster contained the gene, and 0 if the gene was not contained in the significant terms. This procedure resulted in a matrix where each of the 40 clusters is a sample, each gene is a variable, and each elements in the matrix have the value 0 or 1. For the poised RTSSs we collected terms from the same GREAT categories as for the metaclusters, but now we used the general human genomic background provided by GREAT rather than a customized background for the analysis. To evaluate the GREAT terms for the selected repressed RTSSs we compared them to terms generated using random sets of RTSSs with set sizes equal to the selected RTSS sets, and drawn randomly from i) the total set of all repressed RTSSs, and ii) the set of repressed RTSSs with not overlapping significantly enriched chromatin regions from ENCODE. To enumerate the terms related to immune response, we counted terms containing variants of the words *immune, interferon, interleukin, cytokine, inflammation, TNF, NFkB* and *TCF.* For terms related to signaling we only counted variants of the word *signaling*. Associated genes were also extracted from the GREAT reports.

### CpG island enrichment analysis

CpG island coverage and enrichment in RTSS promoters (defined as the region 200 bp upstream of a RTSS) in 40 metaclusters, as well as promoters for genes affected by our selection of poised RTSSs, was computed using the Genomic HyperBrowser [67]. A track of genomic locations for CpG islands was downloaded from the UCSC genome browser, and enrichment factors for each metacluster and gene set were computed as the ratio of observed bp overlap with the CpG island track versus the expected bp overlap across all promoters in each metacluster or gene set. For CpG content of genes affected by our poised RTSSs, we constructed a hypothesis test to investigate whether genes affected in all four cell lines (case) where more significantly enriched for CpG islands than genes affected in one, two or three cell lines (control). The p-value was computed using a Monte Carlo scheme where case and control marks were permuted randomly across all promoter regions of the analysis. Further details on the analysis, including the possibility to reproduce results, is given in a Galaxy page at [70].

### Validation of RTSS-to-gene associations

The correlation between two RTSSs was calculated as Pearson correlation between expression levels over all 975 cell types and tissues analyzed in FANTOM5. For each cluster and the set of poised RTSSs in each cell-line, we used annotated TSSs for genes associated with each cluster from GREAT, and calculated intra-correlations between all RTSSs in increasing distances of 0.05, 0.2, 0.5, 1, 5, 10, 50 and 150 kbp upstream and downstream from the annotated gene TSS. Correlations were calculated for all globally defined RTSSs within the region, and RTSSs contained in each cluster only for the same region. Random correlations were calculated as all intra-correlations between 100 randomly selected RTSSs from the global set.

## Abbreviations

RTSS(s): Robust clusters of transcription start site(s); TSS(s): Transcription start site(s); CAGE: Cap analysis of gene expression; TF: Transcription factor; PCA: Principal component analysis.

RIKEN Omics Science Center ceased to exist as of April 1st, 2013, due to RIKEN reorganization.

## Competing interests

The authors declare that they have no competing interests.

## Authors’ contributions

MR and FD conceived of the project. MR did most of the data analysis and drafted the manuscript. GKS did statistical analysis using the Genomic HyperBrowser. FD supervised the project and contributed to the final manuscript. HK managed the data handling, COD, PC and ARRF were responsible for FANTOM5 management and concept. All authors read and approved the final manuscript.

## Supplementary Material

Additional file 1 The file contains supplementary Figure S1 to S9, S11, S12 and S14 to S16, supplementary Table S10, S17 and S18, and supplementary text S13. Click here for file
