# Supplementary material for: Chromatin states reveal functional associations for globally defined transcription start sites in four human cell lines
Source: BMC Genomics. 2014 Mar 26;15:120. doi: 10.1186/1471-2164-15-120 (PMC3986914; doi:10.1186/1471-2164-15-120)
Supplement: Additional file 1 — The file contains supplementary Figure S1 to S9, S11, S12 and S14 to S16, supplementary Table S10, S17 and S18, and supplementary text S13. [file 1471-2164-15-120-S1.pdf]

Additional file 1

Supplementary figures and tables with legends

S1: Active chromatin marks overlap with repressed RTSS in all four cell lines.

S2: Chromatin profiles for all chromatin marks around the RTSS center position in GM12878, HeLaS3 and HepG2.

S3: Principal Component Analysis (PCA) of H3K3me2 profiles around expressed RTSS in GM12878 displays a continuum of cluster profiles, rather than discrete, separable sets of clusters.

S4: Average subprofiles for 12 chromatin marks in all cell lines. Note that the profiles of the repressive mark H3K27me3 in HepG2 are different from H3K27me3 in the other cell lines, show resemblance to active marks in HepG2. This observation was not confirmed when we downloaded a second dataset for H3K27me3 in HepG2 from ENCODE. We thus concluded that this dataset was probably suspect, and excluded it from the analysis.

S5: Heatmap of subprofile enrichment in each metacluster for all cell lines.

S6: Average subprofile over all RTSS in each metacluster for each chromatin mark in all cell lines.

S7: Metaclusters have different average RTSS expression level. A) Expression levels for metaclusters can be divided into high (green), intermediate (blue) and low (red). Expression levels displayed in the boxplot have been log2 transformed. B) Metacluster size and average expression level of metaclusters are correlated. Color codes are for the different cell lines K562 (green), GM12878 (red), HeLaS3 (blue) and HepG2 (black).

S8: Number of unique GREAT terms for each metacluster differs in within- and between-cell comparisons. Generally metacluster contain more unique terms compared to metaclusters in the same cell type compared to metaclusters in other cell types. However, there are also considerable cluster-specific variations. A) Permissive GREAT analysis. B) Conservative GREAT analysis.

S9: PCA of metacluster terms from GREAT identifies groups with different functional associations. The two first components from a Principal Component Analysis (PCA) on the functional terms from GREAT for all clusters in all cell lines. Cell line abbreviations are K5 for K562, Gm for GM12878, He for HeLa-S3 and Hp for HepG2, and c1-c10 are cluster indexes as used previously.A) PCA plot for all cluster scores. Clusters with scores in the same quadrant of the PCA plot have related functional terms, and the distance between the two clusters corresponds to the degree of similarity. Clusters with scores close to origo (the crossing of the axes arrows) have few functional terms associated with them, while those with scores further away from origo are enriched for several terms. B) Interpretations of groupings in the PCA plot, with additional cluster properties also taken into consideration. Three main groups are apparent. The terms proximal, normal and distal refers to the localization of RTSS enrichment relative to genes as calculated by GREAT (Figure 6). The more distal clusters with low or intermediate RTSS expression show the strongest functional associations. Displayed PCA plot are from the conservative GREAT analysis.

S10: See Table caption

S11: Average pooled profile of all active chromatin marks for expressed RTSS and the selected subset of poised RTSS in all cell lines.

S12: Average Pol II profile enrichment around expressed RTSS, the subset of selected poised RTSS and all repressed RTSS in all cell lines.

S13: Description if IFNα and IFNγ stimulation

S14: General Pol II levels do not change at poised RTSS in K562 after stimulation with IFNα and IFNγ.

S15: RTSS with increased Pol II signal 30 hours after stimulation by IFNα and IFNγ are depleted for active chromatin marks in the unstimulated state, while some enrichment of active marks are observed at unstimulated RTSS with increased Pol II signal 6 hours after stimulation by IFNγ, but not by IFNα.

S16: Repressed genes associated with poised RTSS by GREAT are enriched for active chromatin marks compared to generally repressed genes, but less enriched than the poised RTSS. The curves are combined averages over RTSS and genes in all four cell lines.

S17: Overview of all datasets from ENCODE downloaded and used for the analysis.

S18: P-values calculated for the overlaps in Figure 1 and S1 using the Genomic Hyperbrowser. Sampling method is either *uniform*, indicating a random shuffling of all RTSS regions within the chromosome arms, or *interseg* which also preserves the distance distribution between RTSS regions in the randomisations.

S1

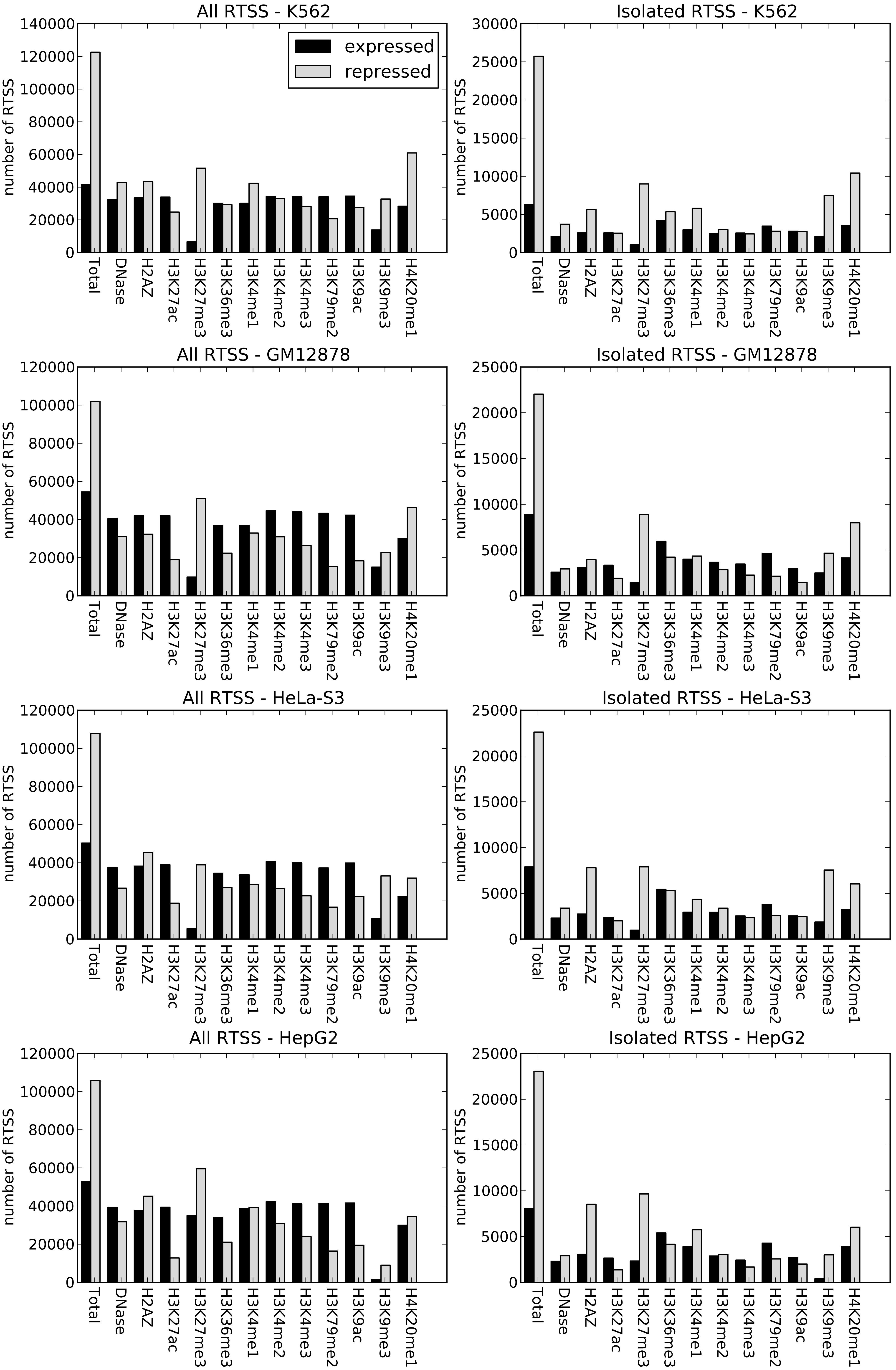

All RTSS - K562
Isolated RTSS - K562
All RTSS - GM12878
Isolated RTSS - GM12878
All RTSS - HeLa-S3
Isolated RTSS - HeLa-S3
All RTSS - HepG2
Isolated RTSS - HepG2
expressed
repressed
number of RTSS
Total
DNase
H2AZ
H3K27ac
H3K27me3
H3K36me3
H3K4me1
H3K4me2
H3K4me3
H3K79me2
H3K9ac
H3K9me3
H4K20me1

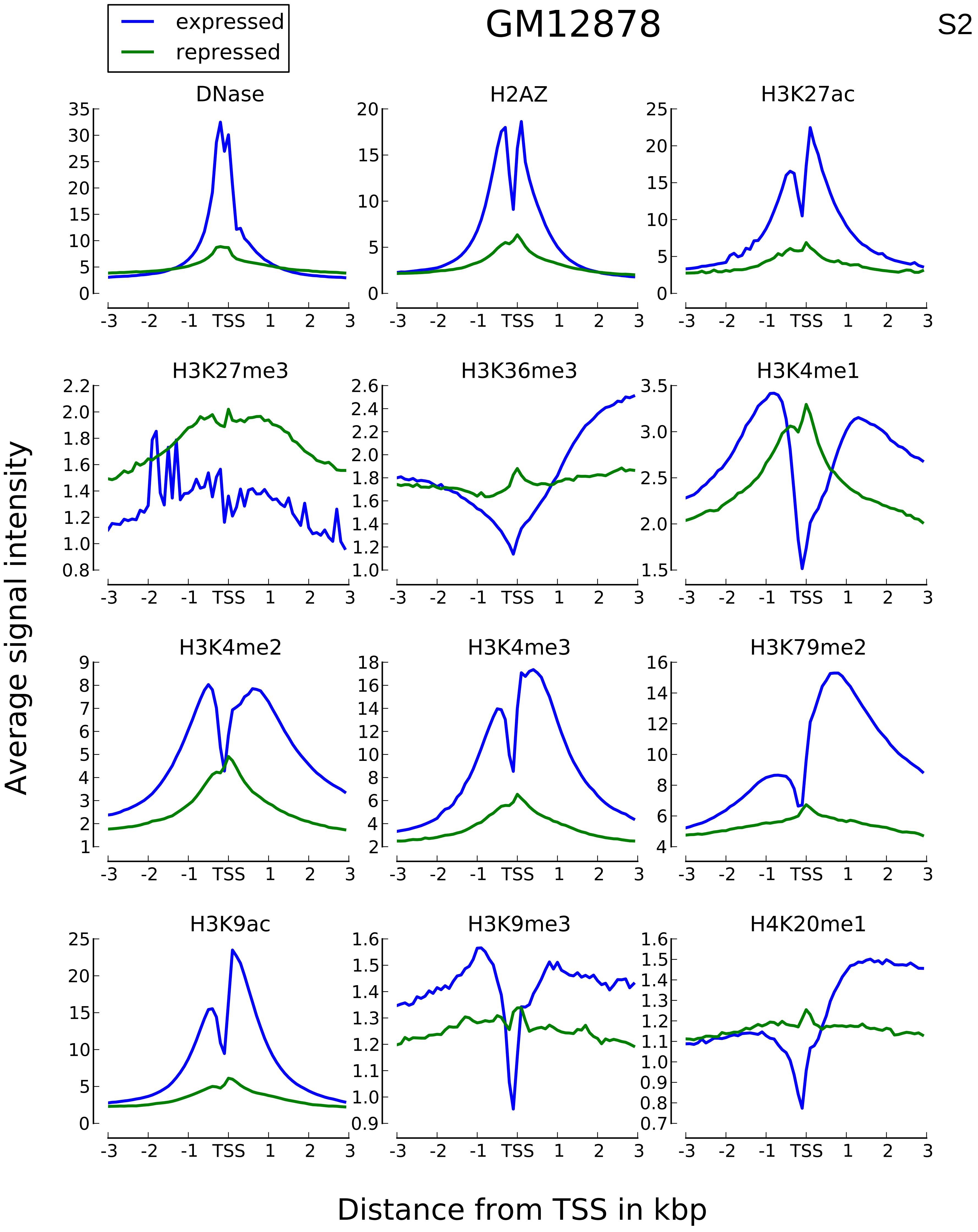

expressed
repressed
GM12878
S2
DNase
H2AZ
H3K27ac
H3K27me3
H3K36me3
H3K4me1
H3K4me2
H3K4me3
H3K79me2
H3K9ac
H3K9me3
H4K20me1
-3 -2 -1 TSS 1 2 3
Average signal intensity
Distance from TSS in kbp

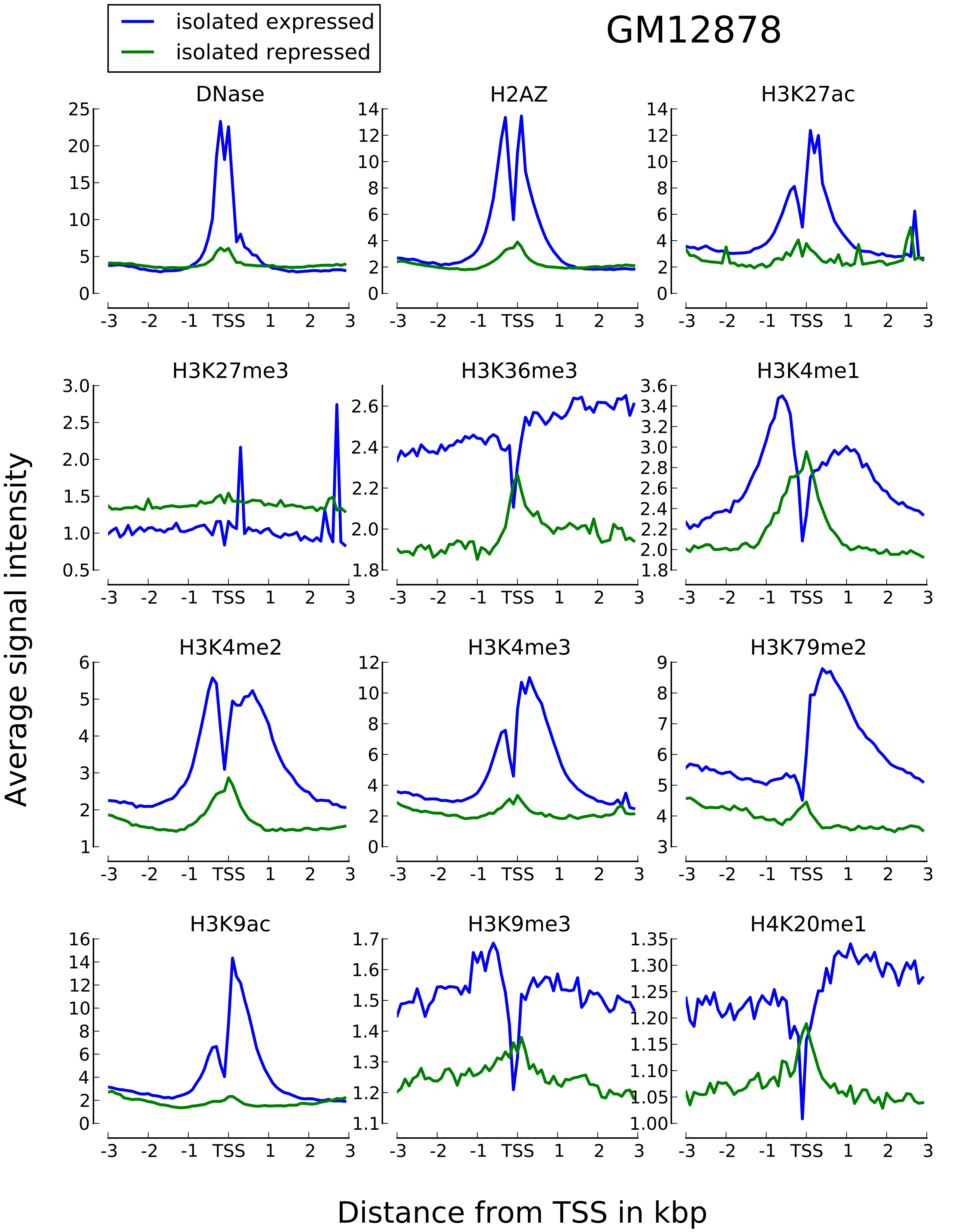

isolated expressed
isolated repressed
GM12878
DNase
H2AZ
H3K27ac
H3K27me3
H3K36me3
H3K4me1
H3K4me2
H3K4me3
H3K79me2
H3K9ac
H3K9me3
H4K20me1
Average signal intensity
Distance from TSS in kbp

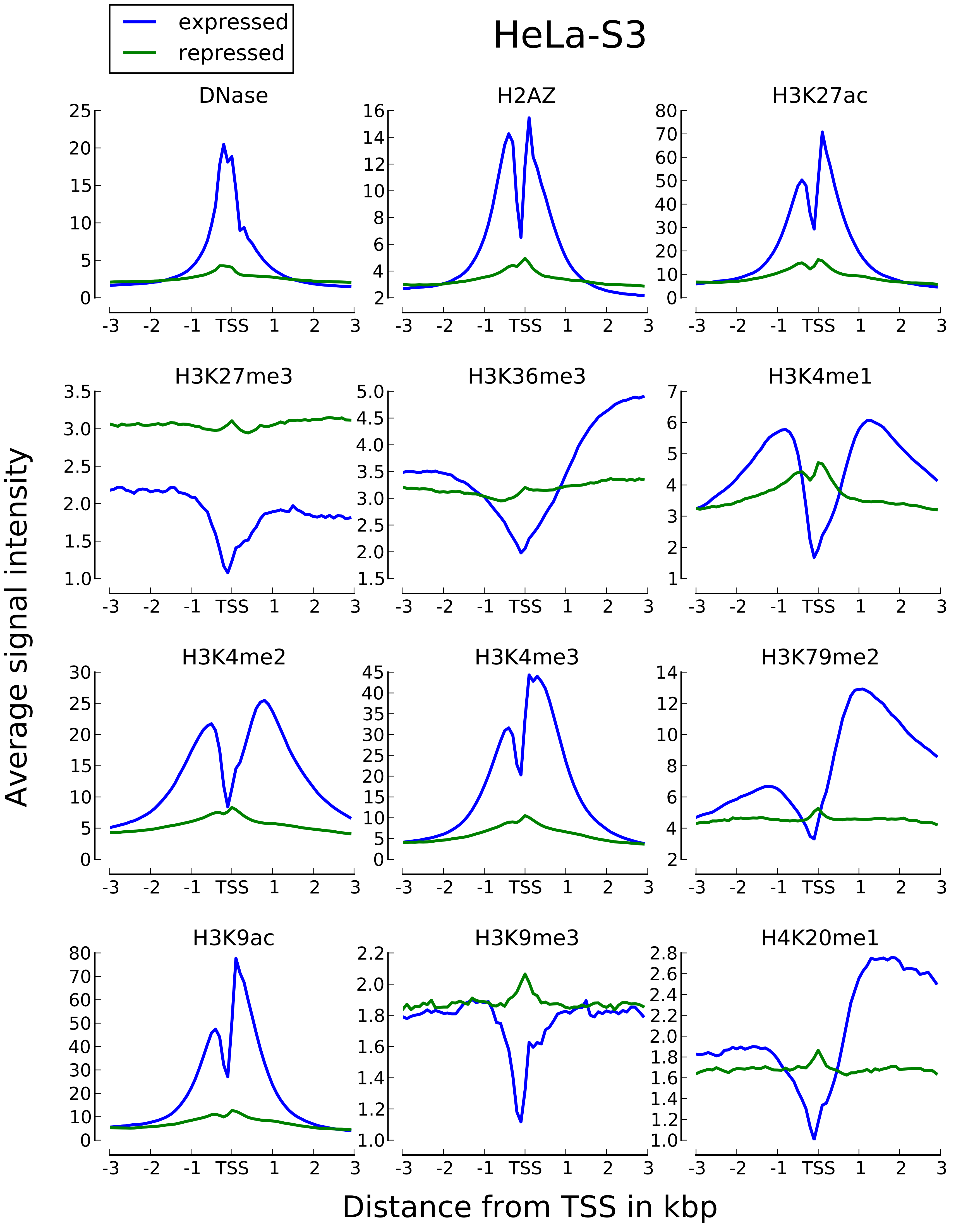

HeLa-S3
expressed
repressed
DNase
H2AZ
H3K27ac
H3K27me3
H3K36me3
H3K4me1
H3K4me2
H3K4me3
H3K79me2
H3K9ac
H3K9me3
H4K20me1
-3 -2 -1 TSS 1 2 3
Average signal intensity
Distance from TSS in kbp

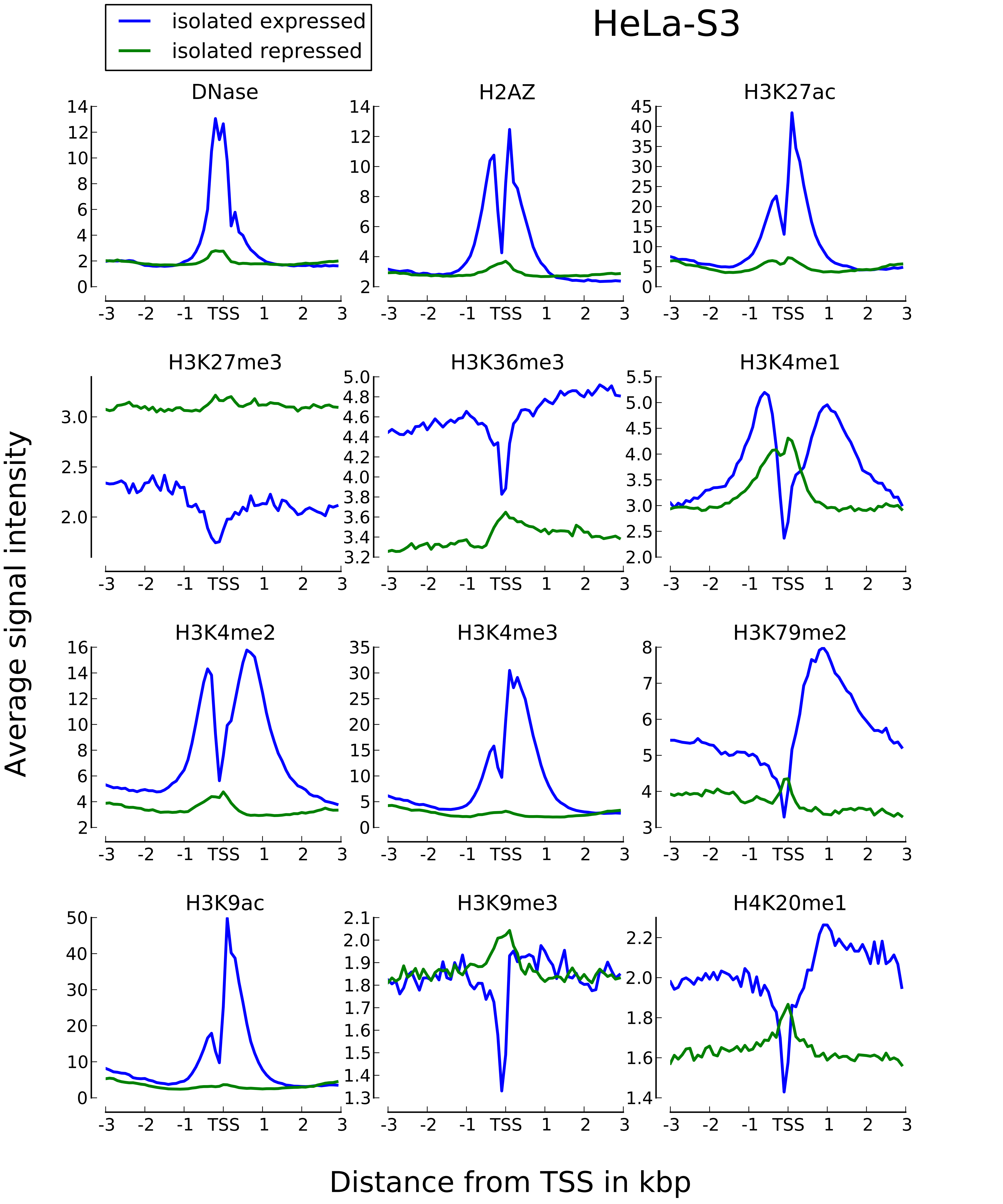

isolated expressed
isolated repressed
HeLa-S3
DNase
H2AZ
H3K27ac
H3K27me3
H3K36me3
H3K4me1
H3K4me2
H3K4me3
H3K79me2
H3K9ac
H3K9me3
H4K20me1
-3 -2 -1 TSS 1 2 3
Average signal intensity
Distance from TSS in kbp

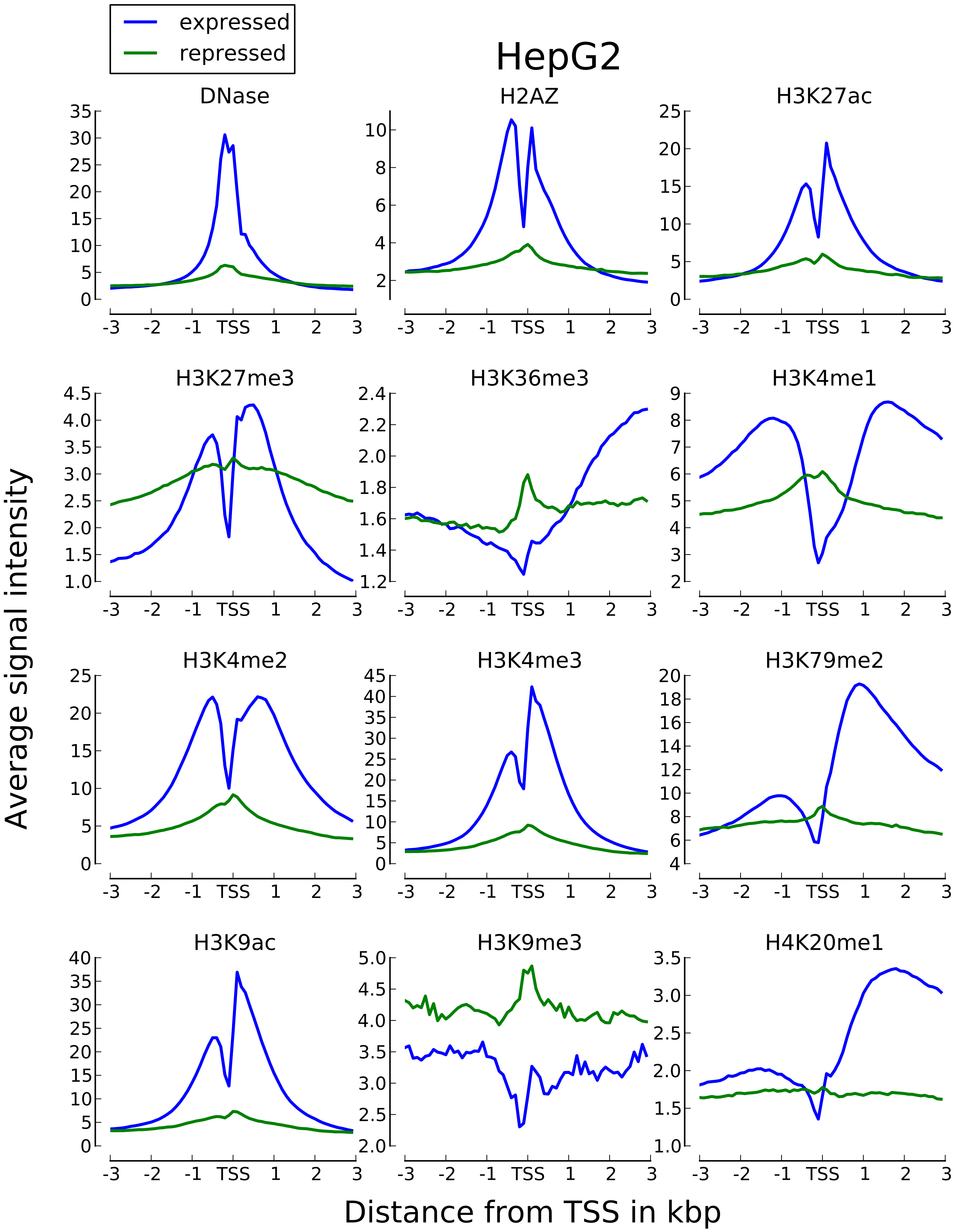

expressed
repressed
HepG2
DNase
H2AZ
H3K27ac
H3K27me3
H3K36me3
H3K4me1
H3K4me2
H3K4me3
H3K79me2
H3K9ac
H3K9me3
H4K20me1
Average signal intensity
Distance from TSS in kbp

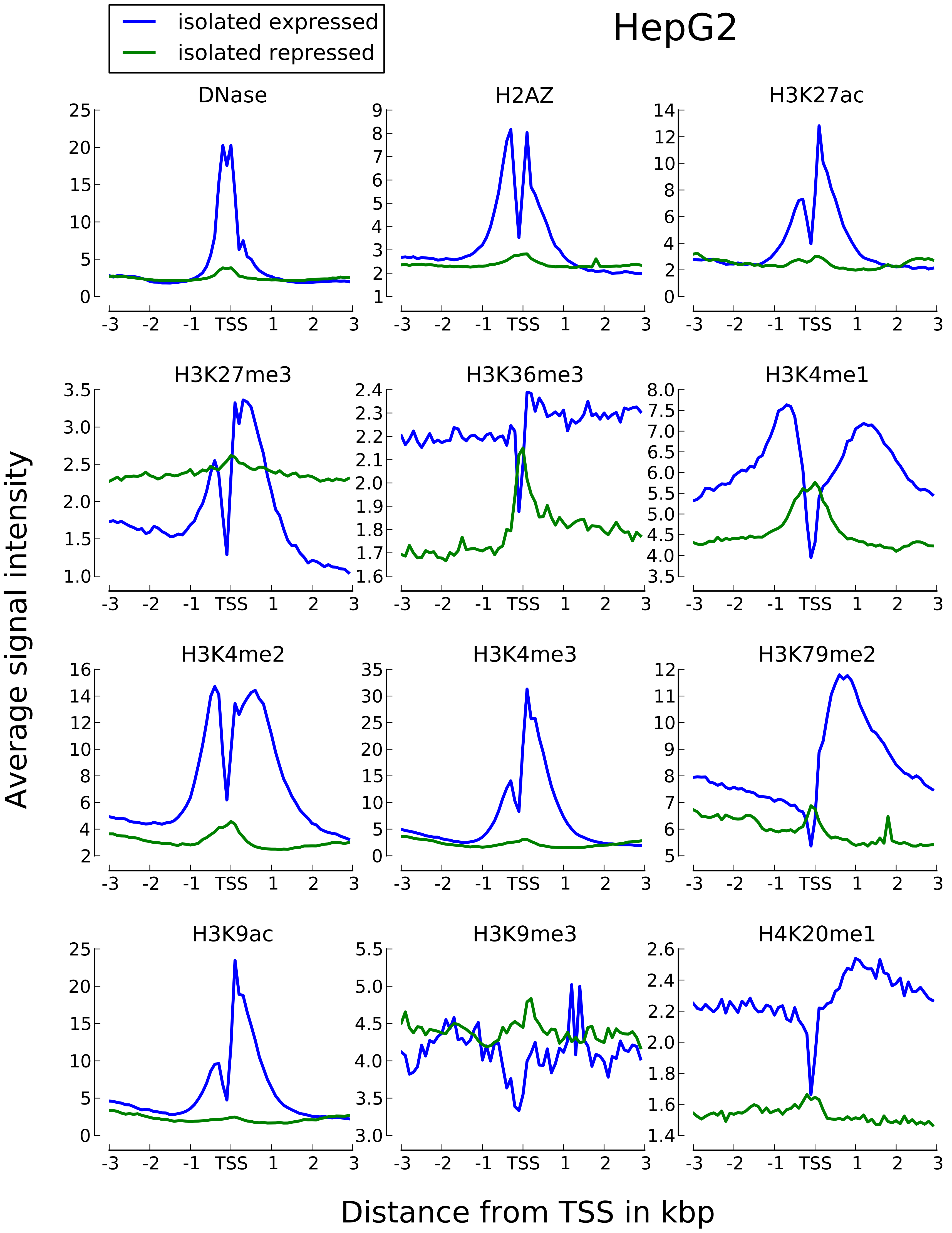

isolated expressed
isolated repressed
HepG2
DNase
H2AZ
H3K27ac
H3K27me3
H3K36me3
H3K4me1
H3K4me2
H3K4me3
H3K79me2
H3K9ac
H3K9me3
H4K20me1
Average signal intensity
Distance from TSS in kbp

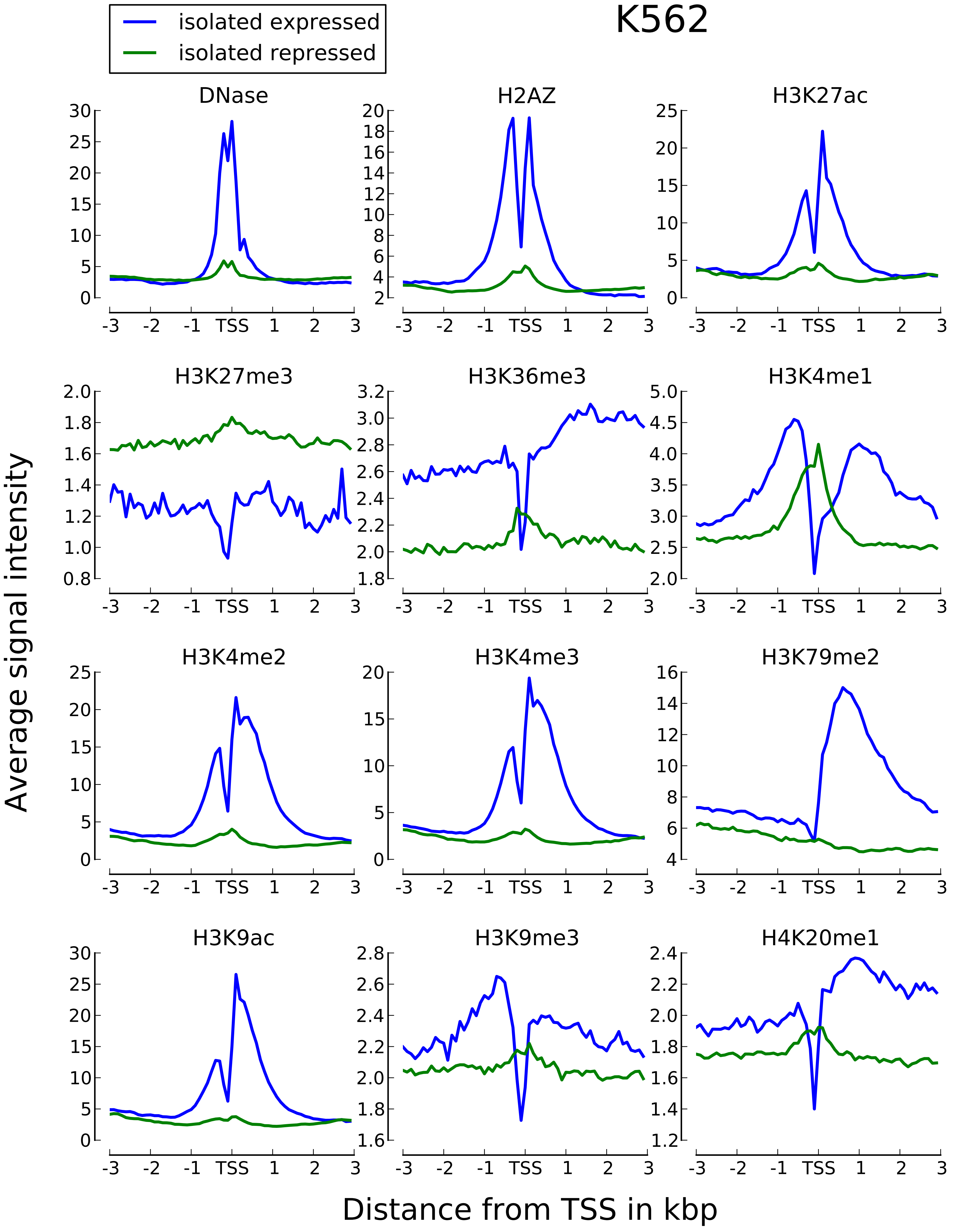

K562
isolated expressed
isolated repressed
DNase
H2AZ
H3K27ac
H3K27me3
H3K36me3
H3K4me1
H3K4me2
H3K4me3
H3K79me2
H3K9ac
H3K9me3
H4K20me1
Average signal intensity
Distance from TSS in kbp

S3

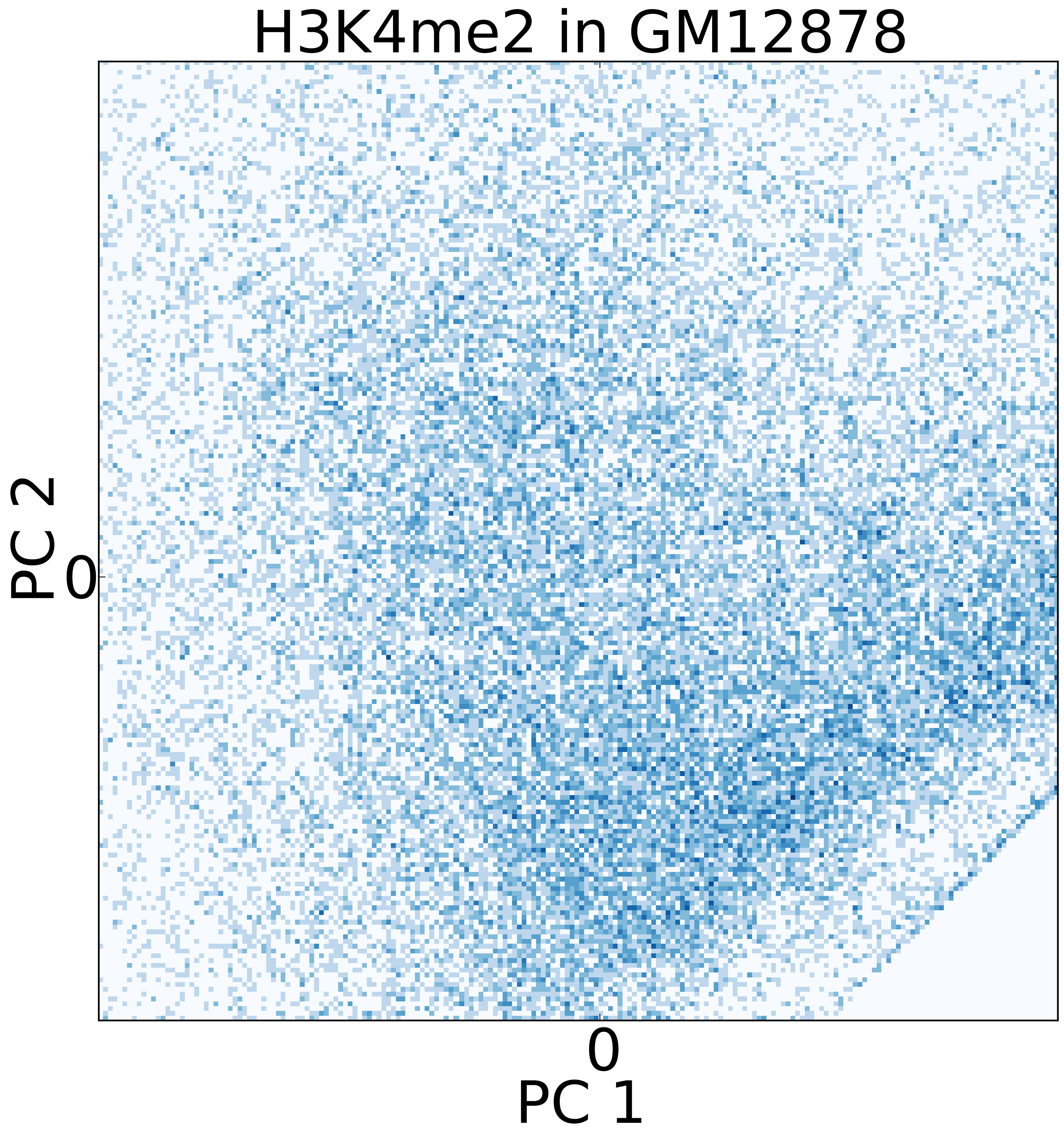

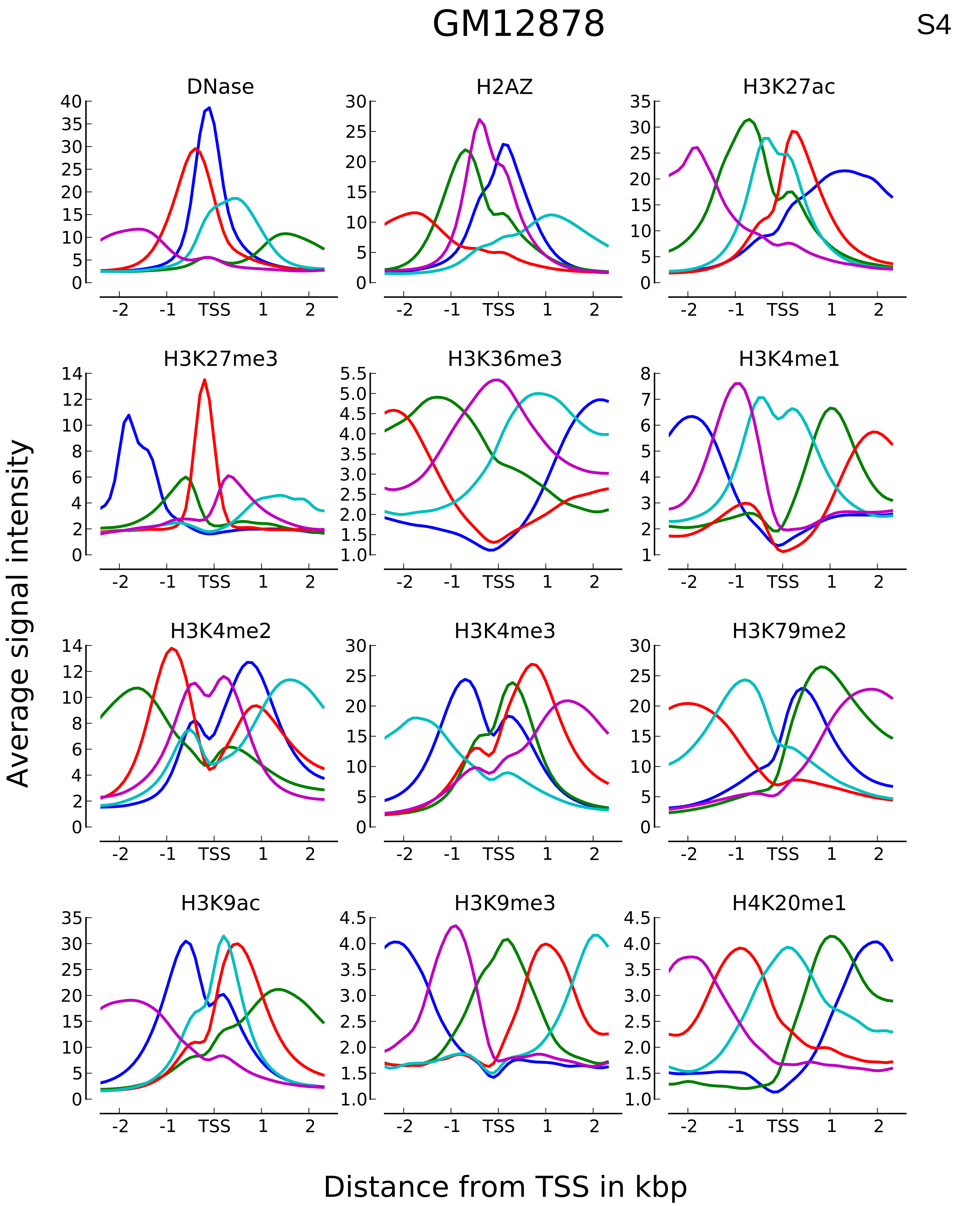
GM12878
S4
DNase
H2AZ
H3K27ac
H3K27me3
H3K36me3
H3K4me1
H3K4me2
H3K4me3
H3K79me2
H3K9ac
H3K9me3
H4K20me1
Average signal intensity
Distance from TSS in kbp
-2
-1
TSS
1
2

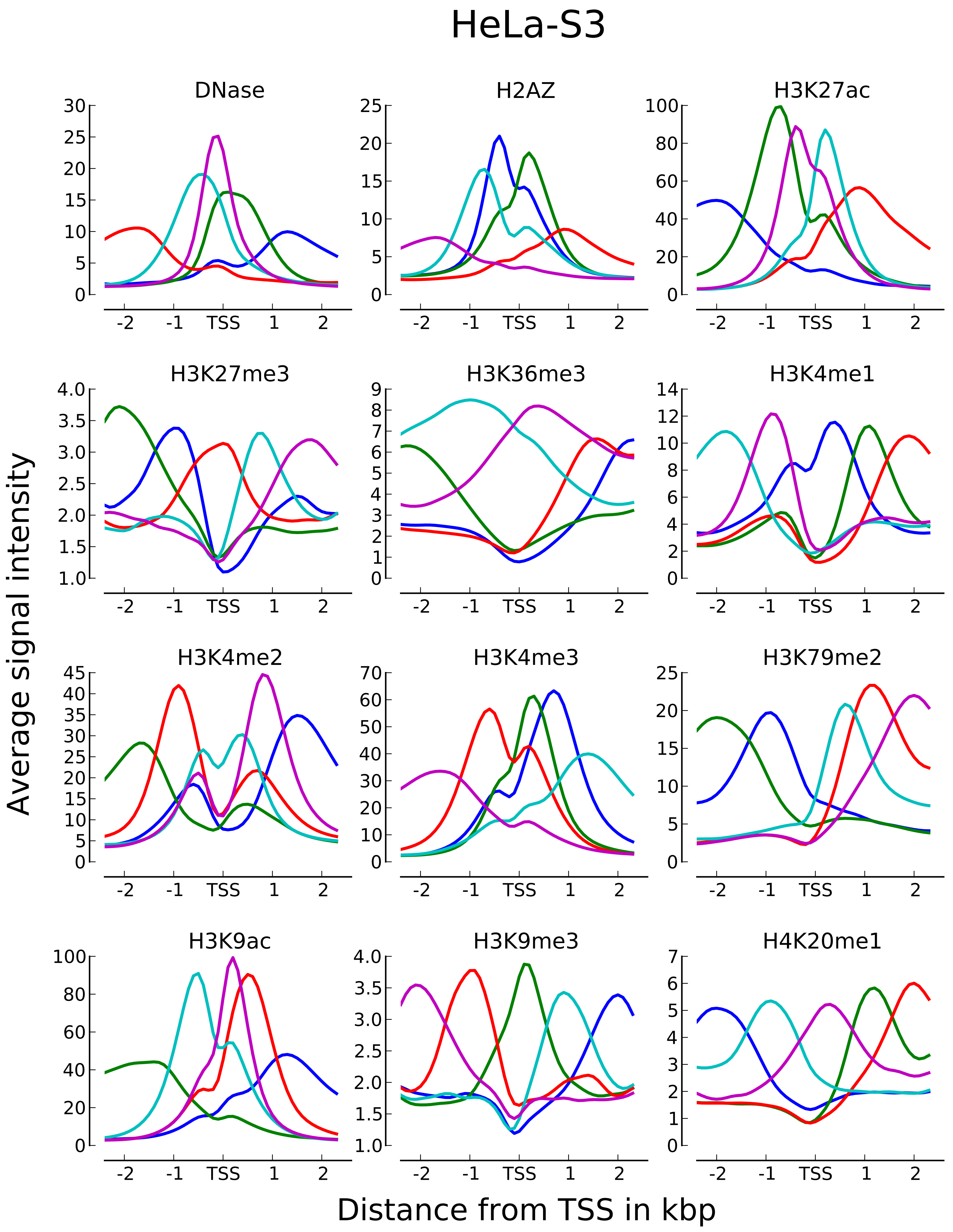

HeLa-S3
DNase
H2AZ
H3K27ac
H3K27me3
H3K36me3
H3K4me1
H3K4me2
H3K4me3
H3K79me2
H3K9ac
H3K9me3
H4K20me1
Average signal intensity
Distance from TSS in kbp
-2
-1
TSS
1
2

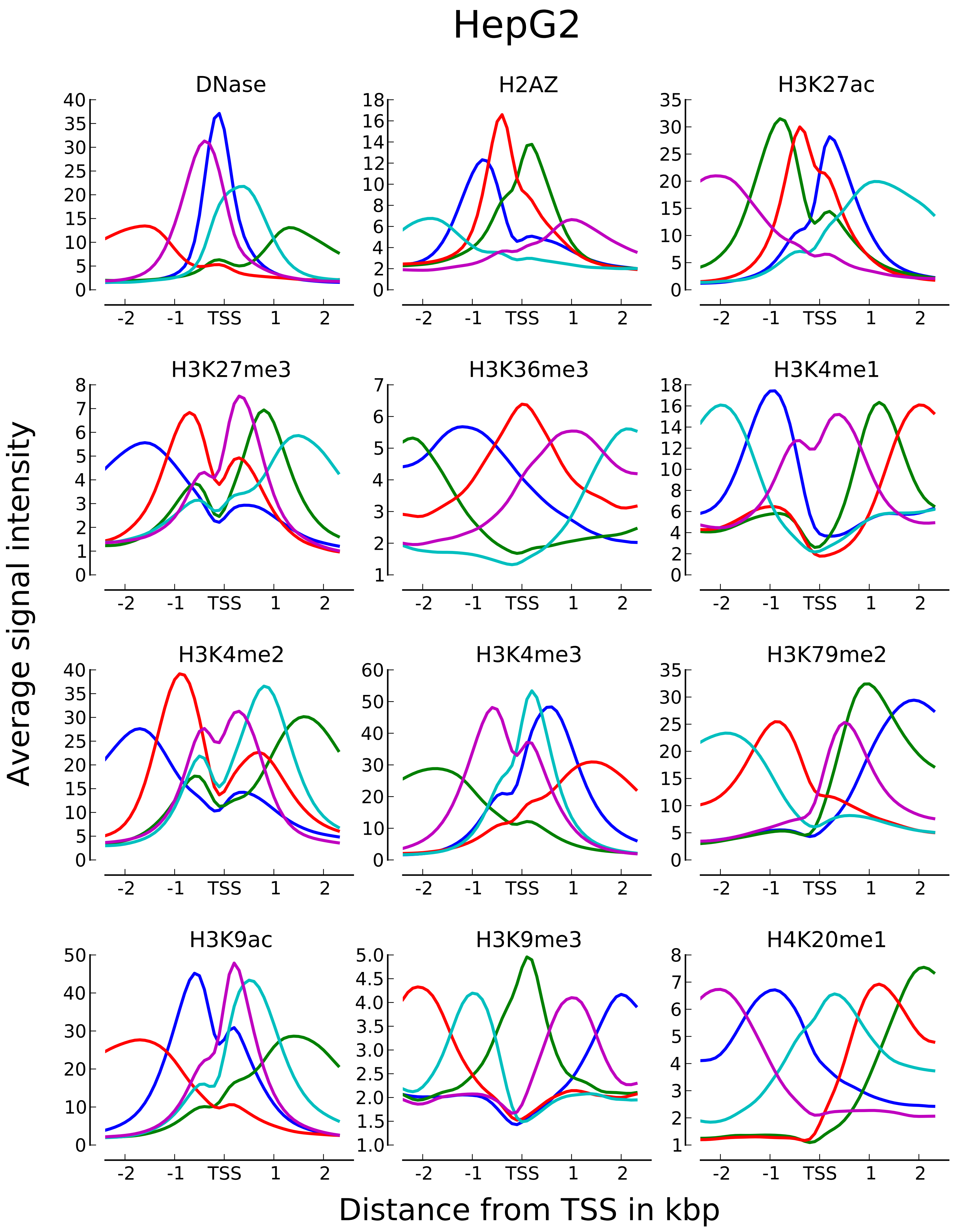
HepG2
DNase
H2AZ
H3K27ac
H3K27me3
H3K36me3
H3K4me1
H3K4me2
H3K4me3
H3K79me2
H3K9ac
H3K9me3
H4K20me1
Average signal intensity
Distance from TSS in kbp
-2
-1
TSS
1
2

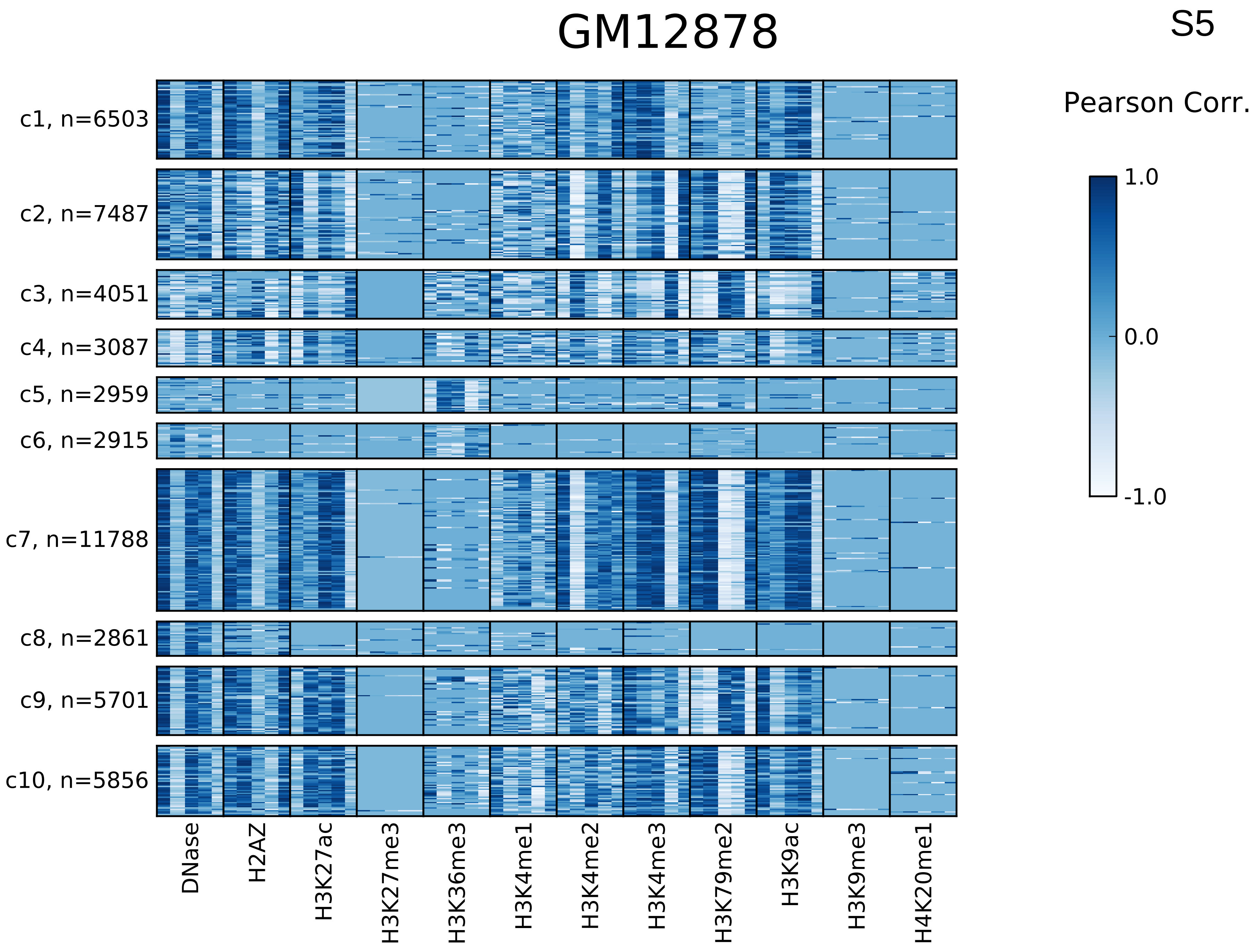
GM12878
S5
Pearson Corr.
1.0
0.0
-1.0
c1, n=6503
c2, n=7487
c3, n=4051
c4, n=3087
c5, n=2959
c6, n=2915
c7, n=11788
c8, n=2861
c9, n=5701
c10, n=5856
DNase
H2AZ
H3K27ac
H3K27me3
H3K36me3
H3K4me1
H3K4me2
H3K4me3
H3K79me2
H3K9ac
H3K9me3
H4K20me1

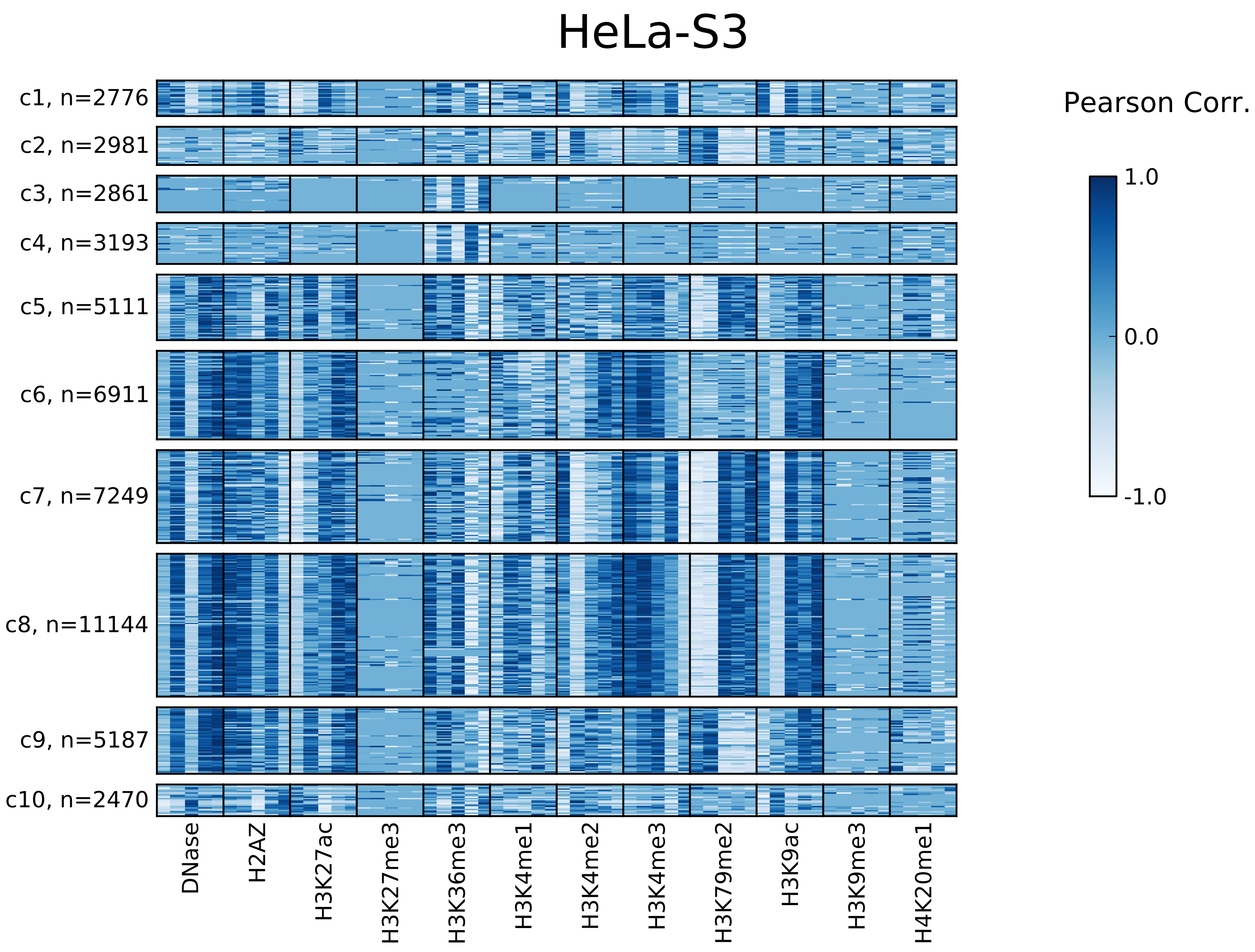
HeLa-S3
Pearson Corr.
1.0
0.0
-1.0
c1, n=2776
c2, n=2981
c3, n=2861
c4, n=3193
c5, n=5111
c6, n=6911
c7, n=7249
c8, n=11144
c9, n=5187
c10, n=2470
DNase
H2AZ
H3K27ac
H3K27me3
H3K36me3
H3K4me1
H3K4me2
H3K4me3
H3K79me2
H3K9ac
H3K9me3
H4K20me1

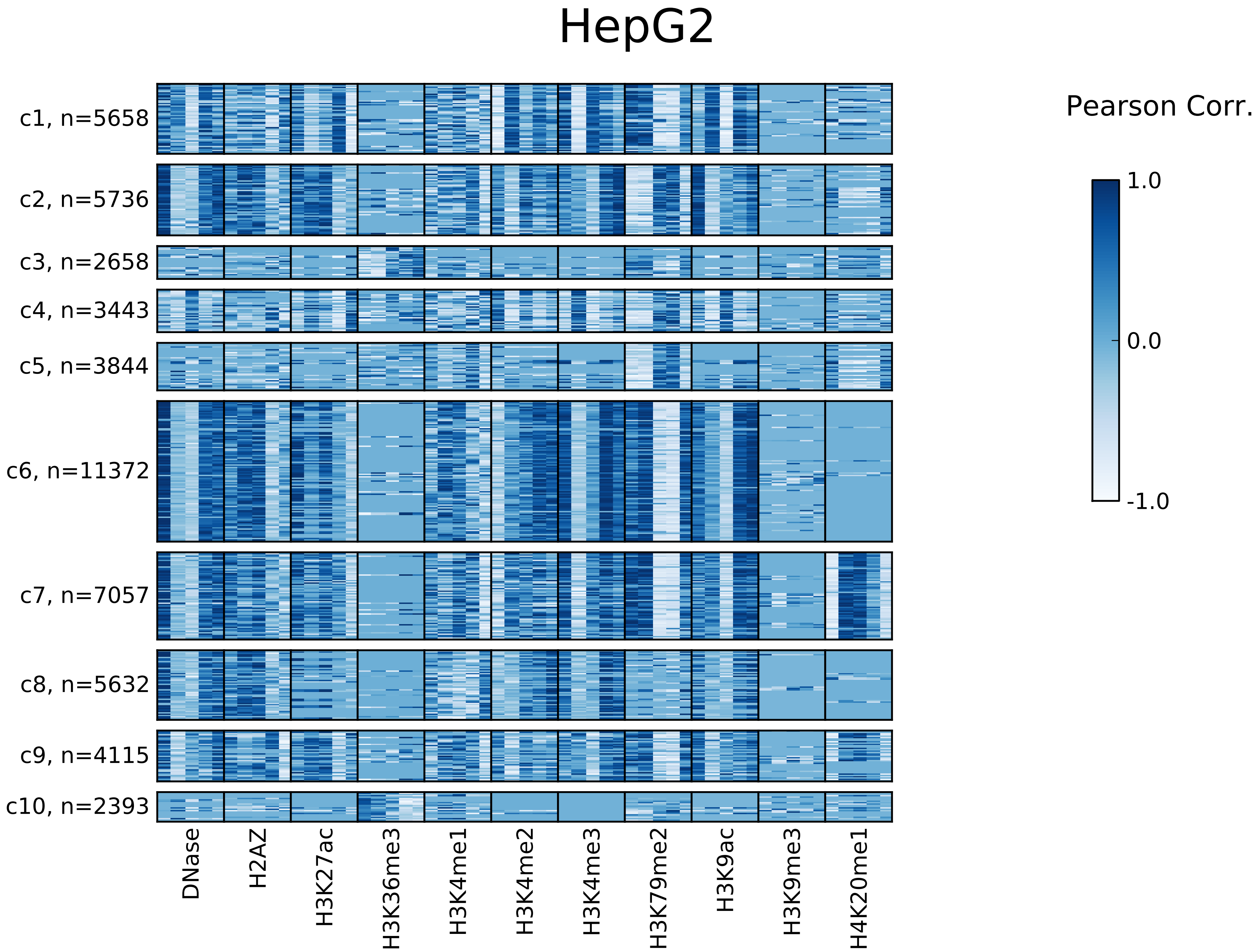

HepG2
Pearson Corr.
1.0
0.0
-1.0
c1, n=5658
c2, n=5736
c3, n=2658
c4, n=3443
c5, n=3844
c6, n=11372
c7, n=7057
c8, n=5632
c9, n=4115
c10, n=2393
DNase
H2AZ
H3K27ac
H3K36me3
H3K4me1
H3K4me2
H3K4me3
H3K79me2
H3K9ac
H3K9me3
H4K20me1

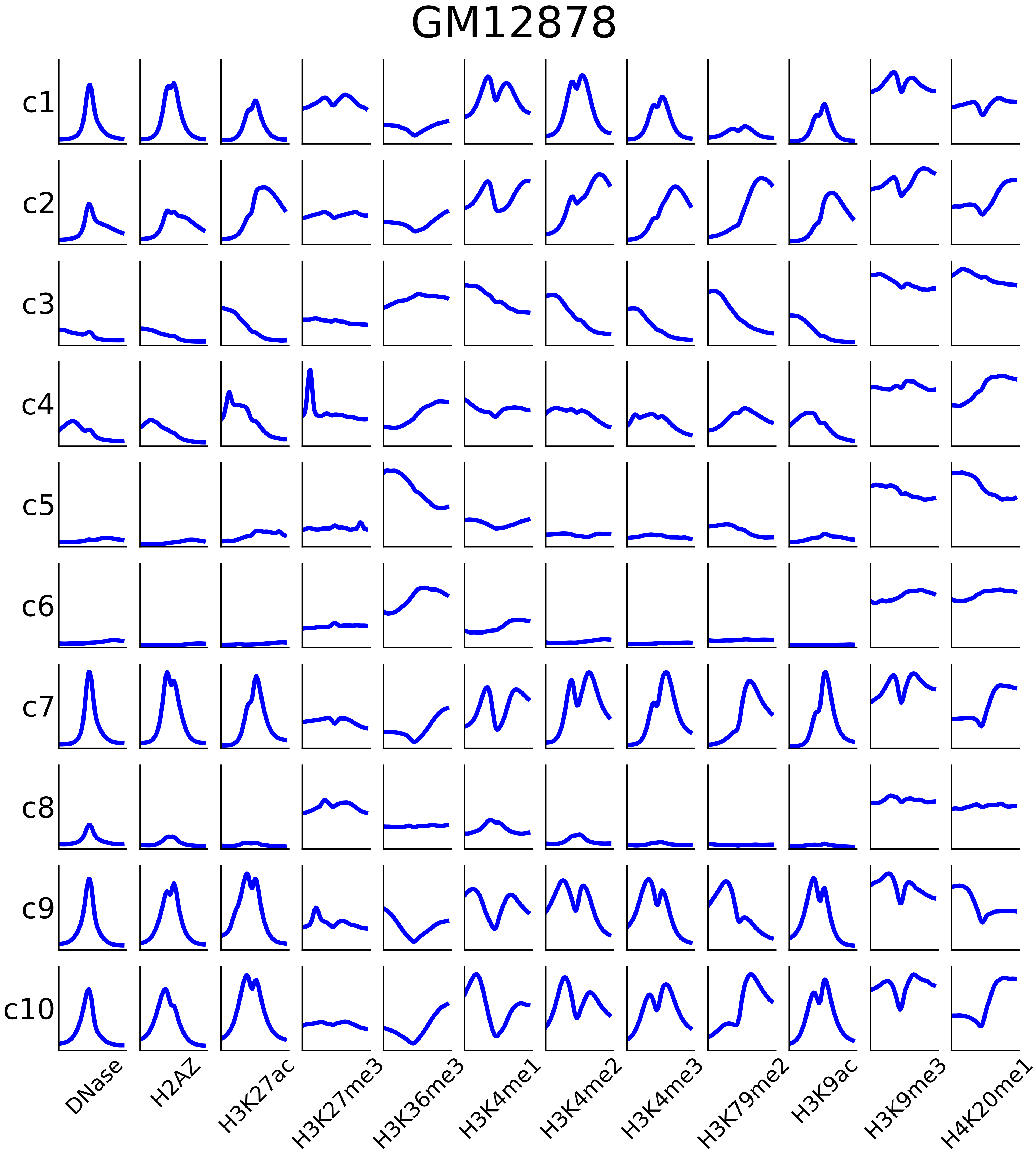
GM12878
c1
c2
c3
c4
c5
c6
c7
c8
c9
c10
DNase
H2AZ
H3K27ac
H3K27me3
H3K36me3
H3K4me1
H3K4me2
H3K4me3
H3K79me2
H3K9ac
H3K9me3
H4K20me1

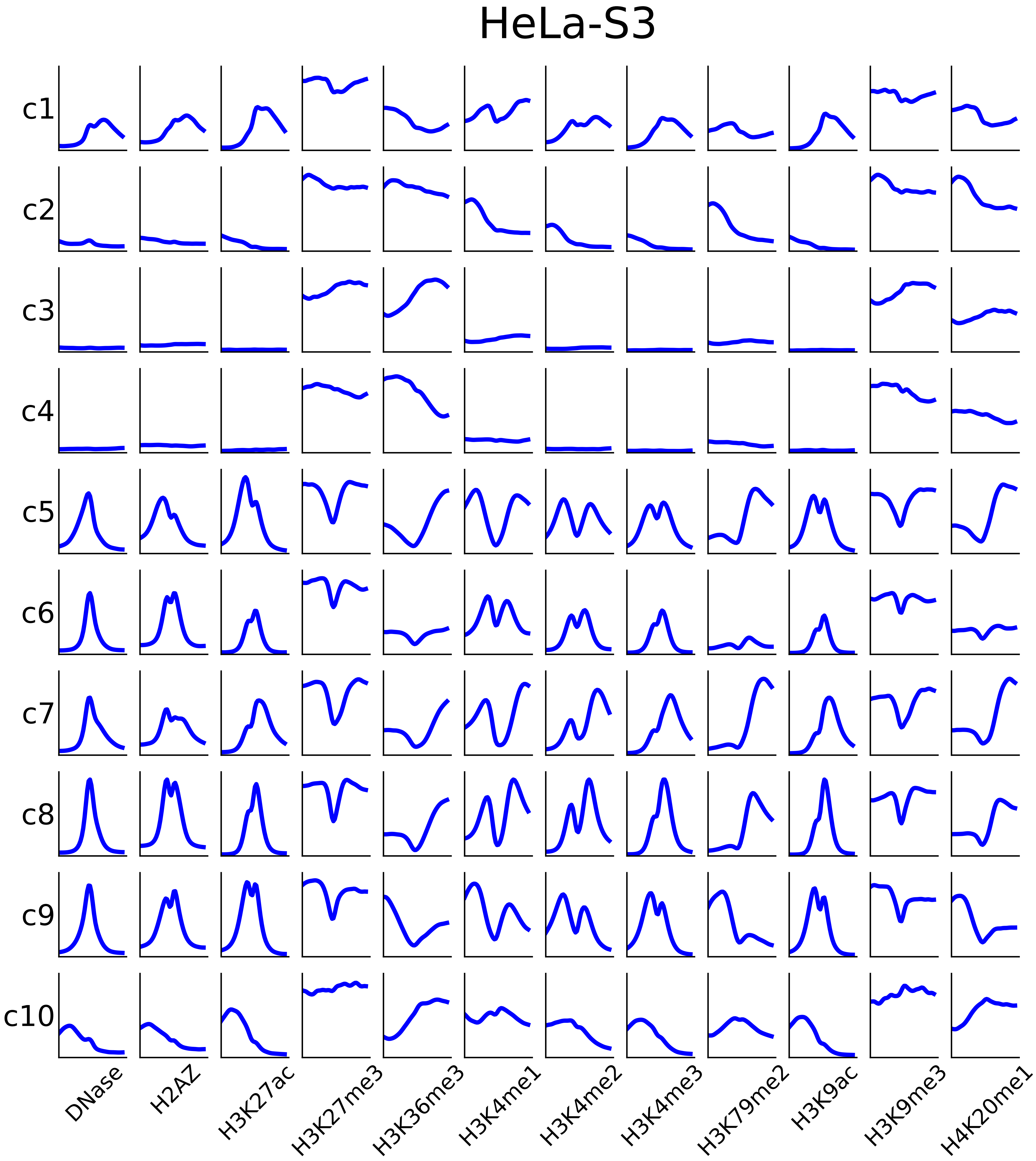

HeLa-S3
c1
c2
c3
c4
c5
c6
c7
c8
c9
c10
DNase
H2AZ
H3K27ac
H3K27me3
H3K36me3
H3K4me1
H3K4me2
H3K4me3
H3K79me2
H3K9ac
H3K9me3
H4K20me1

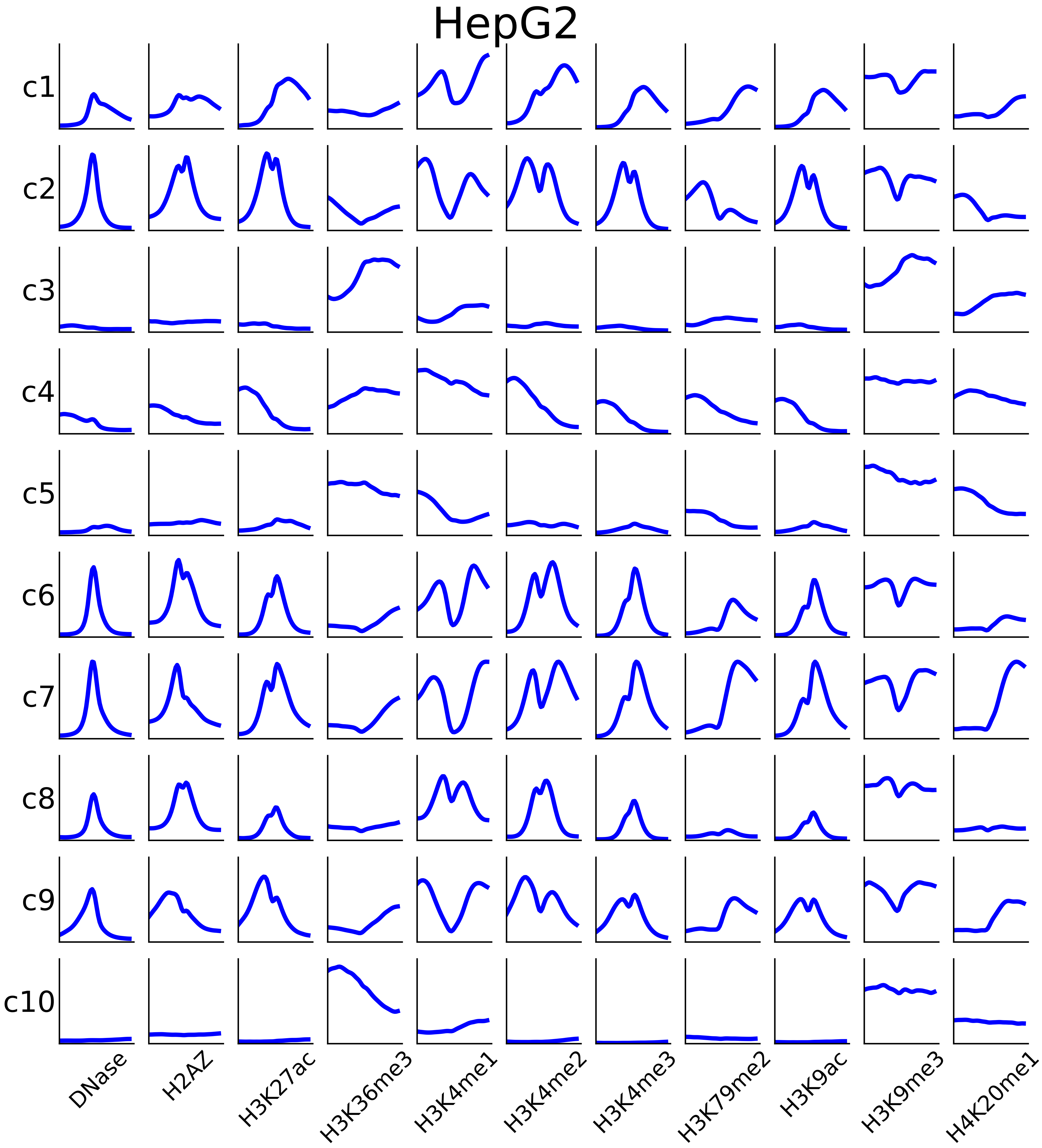
HepG2
c1
c2
c3
c4
c5
c6
c7
c8
c9
c10
DNase
H2AZ
H3K27ac
H3K36me3
H3K4me1
H3K4me2
H3K4me3
H3K79me2
H3K9ac
H3K9me3
H4K20me1

S7

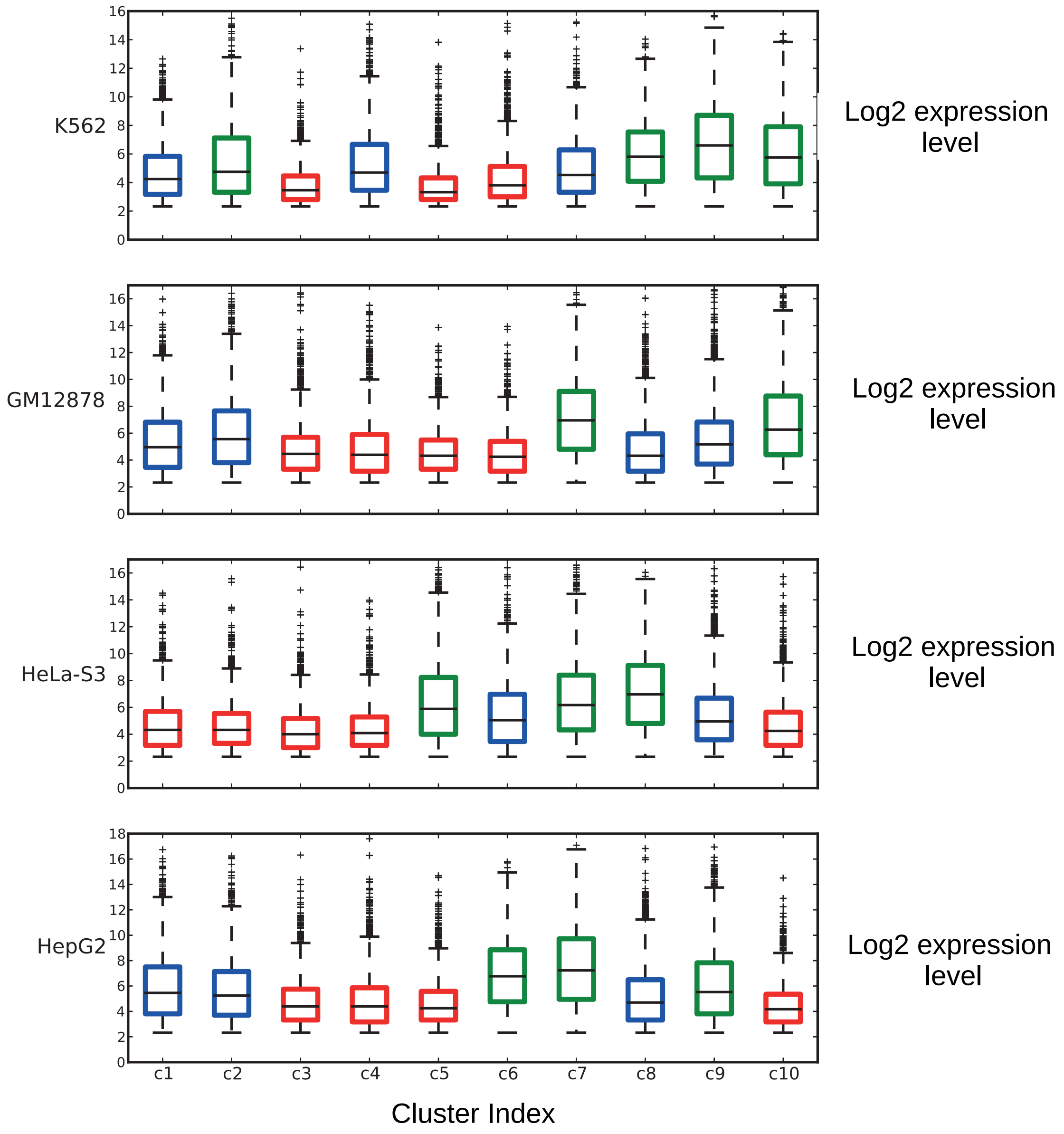

K562
GM12878
HeLa-S3
HepG2
Log2 expression level
Log2 expression level
Log2 expression level
Log2 expression level
c1
c2
c3
c4
c5
c6
c7
c8
c9
c10
Cluster Index

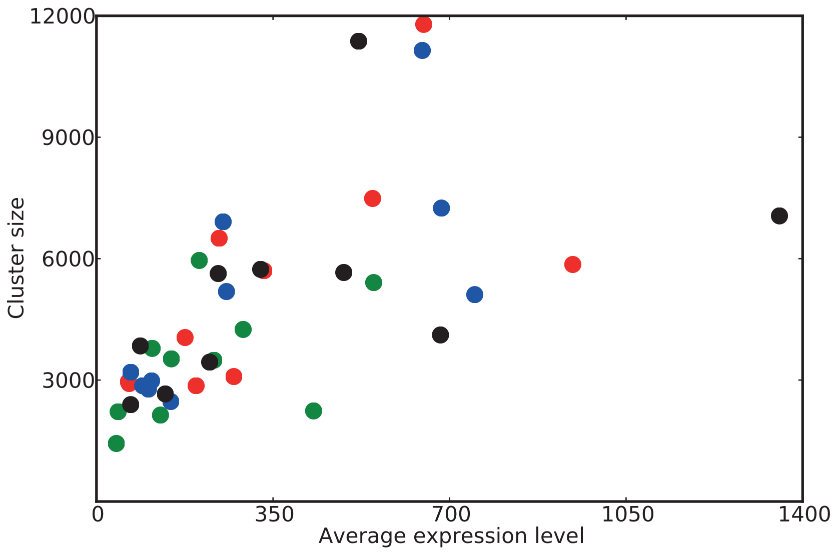

Cluster size
Average expression level
12000
9000
6000
3000
0
350
700
1050
1400

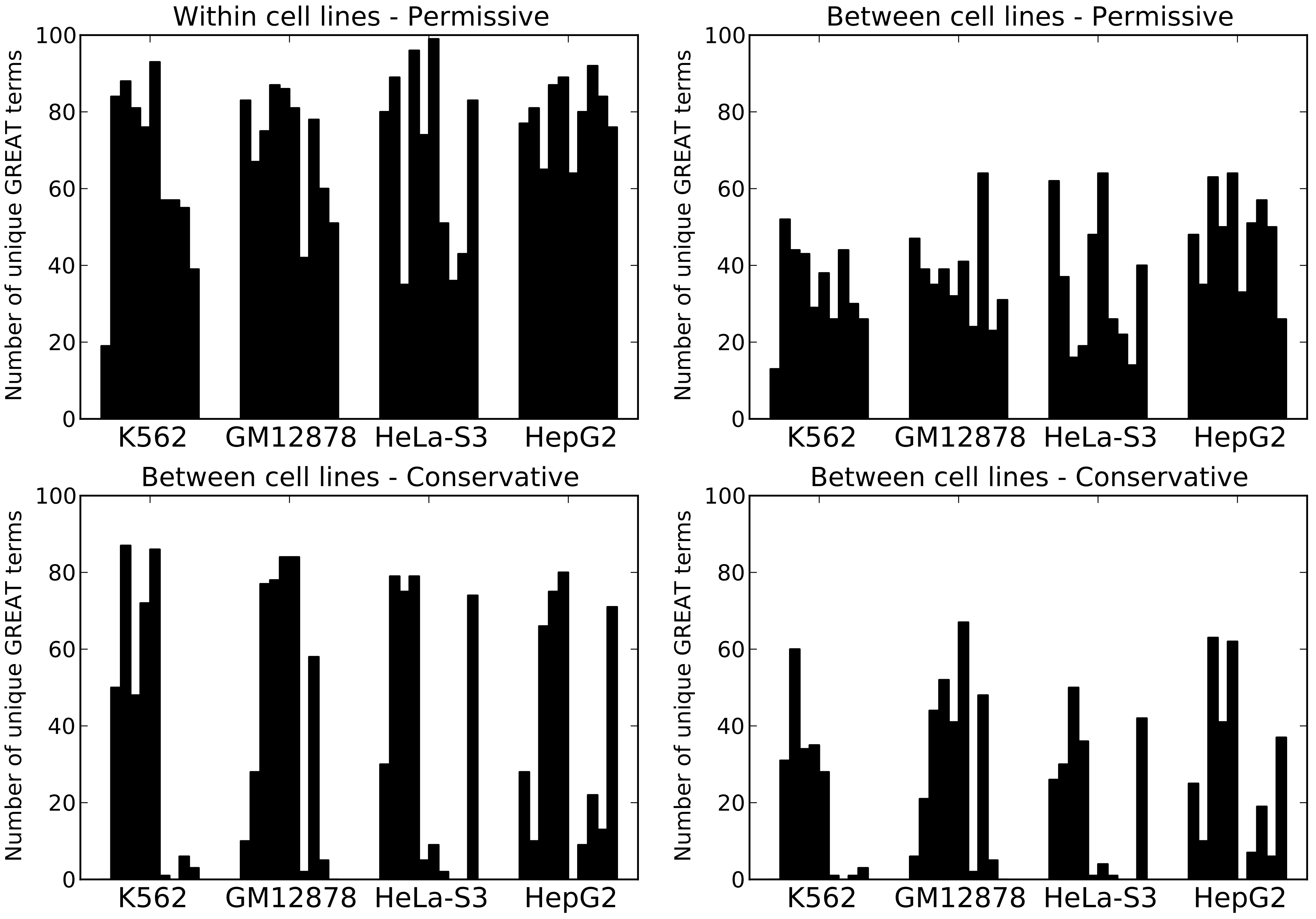
Within cell lines - Permissive
Between cell lines - Permissive
Between cell lines - Conservative
Between cell lines - Conservative
Number of unique GREAT terms
0
20
40
60
80
100
K562
GM12878
HeLa-S3
HepG2

A

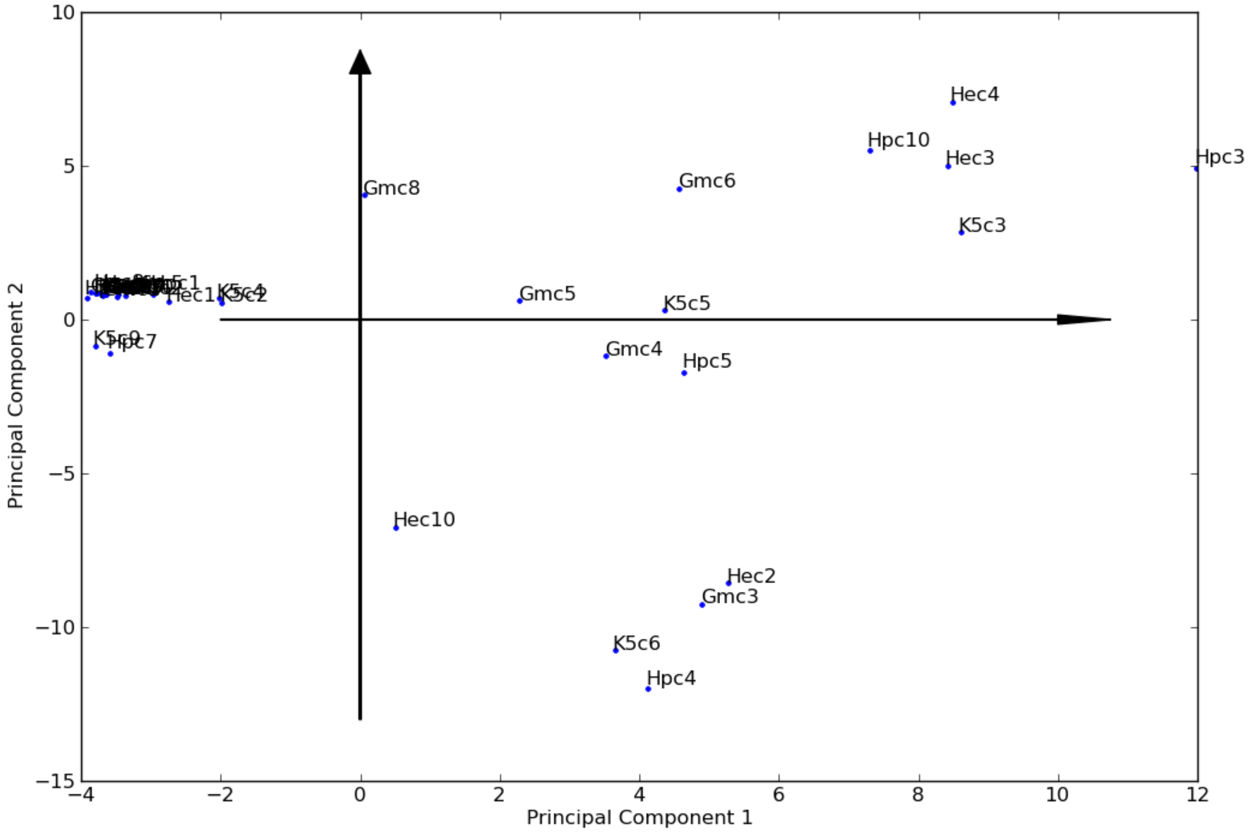

Principal Component 2
Principal Component 1
Hec4
Hpc10
Hec3
Hpc3
Gmc8
Gmc6
K5c3
Gmc5
K5c5
Gmc4
Hpc5
Hec10
Hec2
Gmc3
K5c6
Hpc4
K5c4
K5c2
Hec1
Hpc7

B

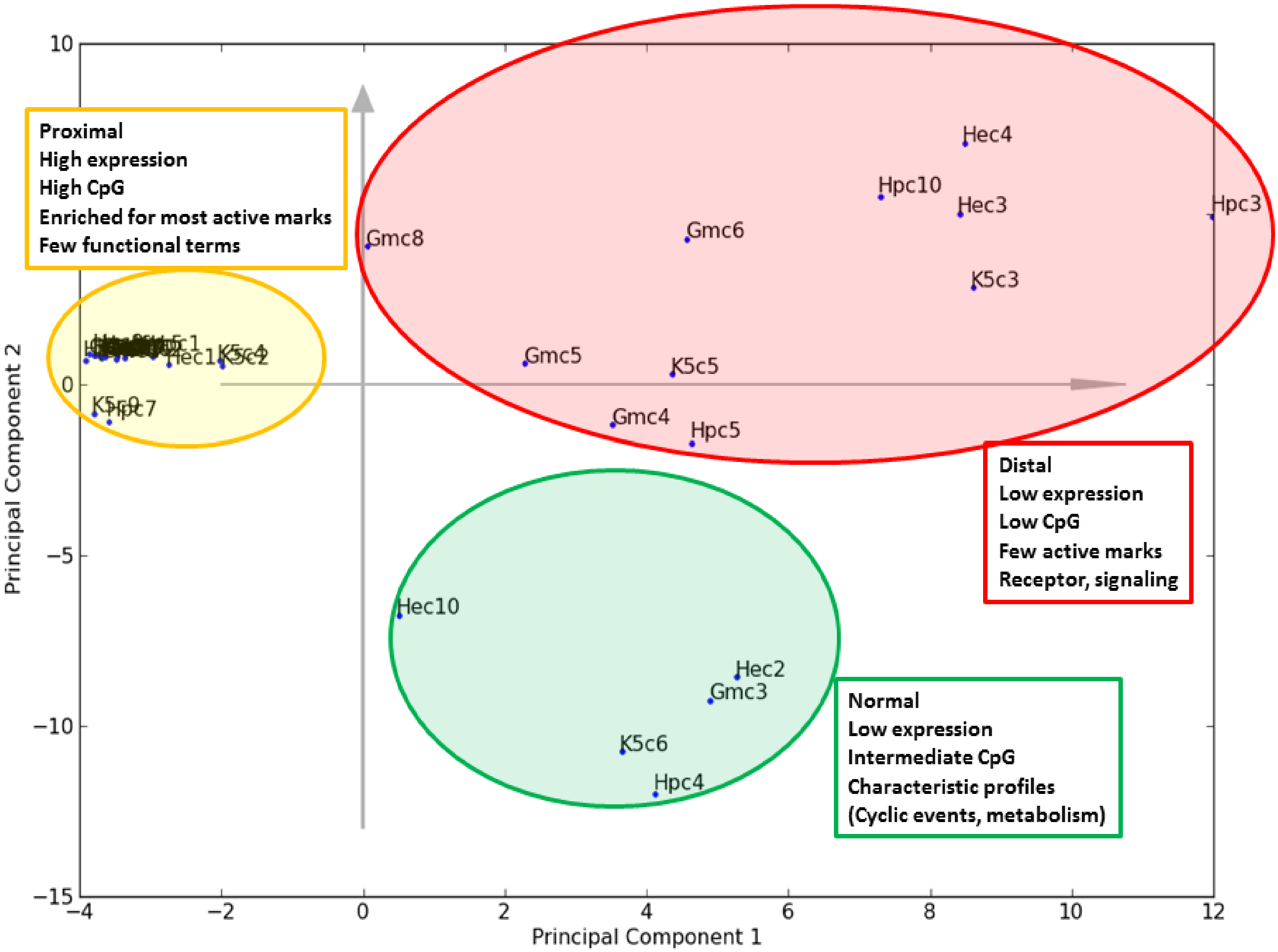

Proximal
High expression
High CpG
Enriched for most active marks
Few functional terms
Distal
Low expression
Low CpG
Few active marks
Receptor, signaling
Normal
Low expression
Intermediate CpG
Characteristic profiles
(Cyclic events, metabolism)
Principal Component 2
Principal Component 1
Hec4
Hpc10
Hec3
Hpc3
Gmc8
Gmc6
K5c3
Gmc5
K5c5
Gmc4
Hpc5
Hec10
Hec2
Gmc3
K5c6
Hpc4
K5c4
K5c2
Hec1
Hpc7

Supplementary Table S10: Filtering of repressed RTSS with active marks.

	Total repressed	Peak-overlap	Signal > 1000	Corr. > 0.5	p-value ≤ 0.05	Filtered subset
K562	122 575	99 100	34 279	20 948	26 018	6 184
GM12878	101 964	80 594	24 625	17 775	19 297	3 813
HeLaS3	107 756	90 982	34 900	11 946	18 249	4 345
HepG2	105 793	91 323	28 950	18 325	25 541	4 303

S11

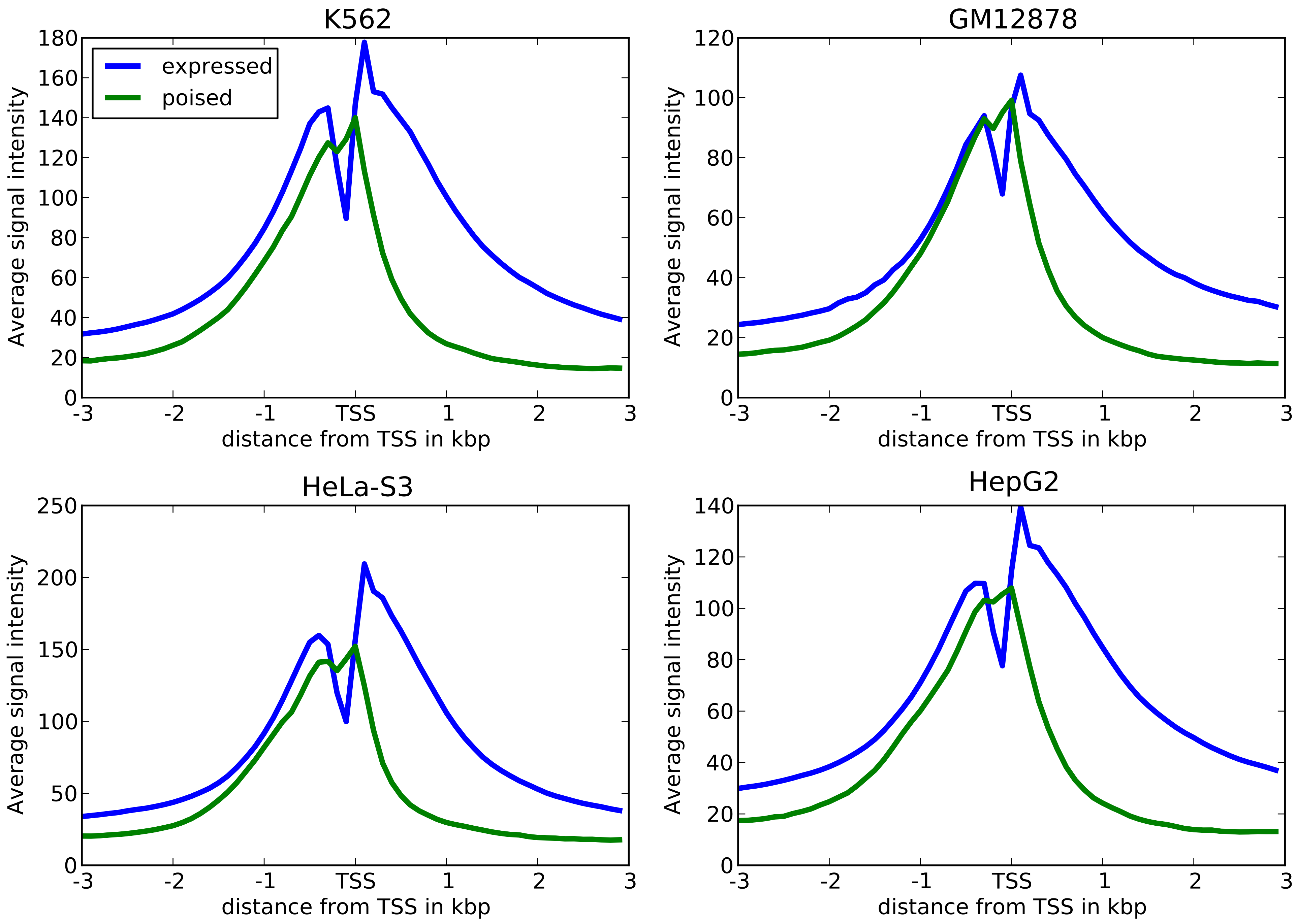

K562
GM12878
HeLa-S3
HepG2
expressed
poised
Average signal intensity
distance from TSS in kbp
-3
-2
-1
TSS
1
2
3

S12

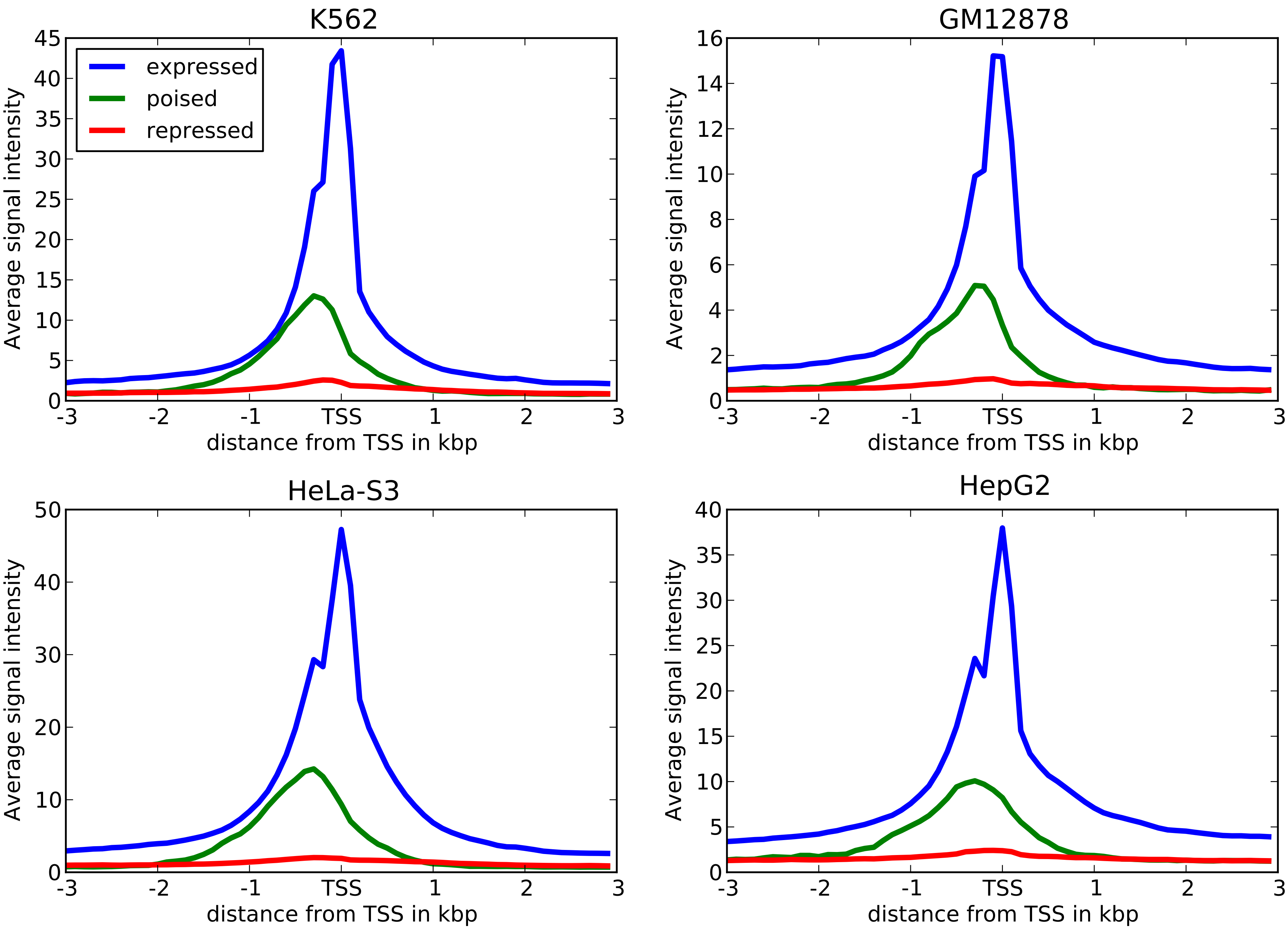
K562
GM12878
HeLa-S3
HepG2
expressed
poised
repressed
Average signal intensity
distance from TSS in kbp
TSS
-3
-2
-1
1
2
3
0
5
10
15
20
25
30
35
40
45
2
4
6
8
12
14
16
50

Supplementary S13

Poised RTSS and response to stimulation by IFNα and IFNγ

In this analysis we wanted to investigate whether our selected poised RTSS would respond to external stimuli. For this analysis, we could only find one relevant dataset from ENCODE. This was ChIP-Seq data for Pol II in unstimulated cells, and for Pol II in cells stimulated by IFNα and IFNγ (ENCODE, SYDH TFBS, Stanford/Yale/USC/Harvard) , both sampled after 6 and 30 hours. We observed similar levels of Pol II for our selected RTSS relative to Pol II levels for expressed RTSS before and after stimulation of both IFNα and IFNγ (Supplementary S13). This was in contrast to the previous studies which reported a general increase in Pol II levels after stimulation (Hargreaves et al. 2009).

Because of this inconsistency we further investigated RTSS that were actually stimulated by IFNα and IFNγ. To identify the RTSS most strongly stimulated in the four datasets, the absolute difference and the ratio of Pol II levels between stimulated and unstimulated cells were calculated for each of 179 369 globally defined RTSS in a ±300bp window around the RTSS center. In each stimulated dataset, the RTSS absolute difference and ratio values were sorted independently in descending order, and the total enrichment score for each RTSS calculated as the sum of the ranks in the two sorted lists. The 5000 RTSS with the highest rank for each of the four stimulated datasets were used in the analysis.

The top 5000 RTSS with increased Pol II signal after IFNα and IFNγ stimulation showed a poor overlap with our poised RTSS (123 (2%) and 194 (3%) RTSS overlap, respectively), while the overlap between IFNα and IFNγ RTSS with increased Pol II was 33%. We observed enrichment of active chromatin marks in IFNγ stimulated RTSS, but this enrichment was less than for our selected poised RTSS. For IFNα stimulated RTSS we observed little enrichment for active chromatin marks (Supplementary S14). We also compared the enrichment of active chromatin marks in IFNα and IFNγ stimulated RTSS after 6 and 30 hours of stimulation, calculating the enrichment over the top 5000 RTSS with the highest increase in Pol II for each dataset (Supplementary S14). The RTSS with the strongest increase in Pol II levels after 30 hours showed a general depletion of active chromatin marks in the unstimulated state.

We see two likely reasons for the absent changes in Pol II levels at our poised RTSS after IFNα and IFNγ stimulation. First, we observe that both the poised RTSS and the genes associated with the RTSS are highly cell type specific, with most RTSS and genes found in only one cell type. In addition we observed that the RTSS and genes affected by IFNα and IFNγ have a similar degree of overlap as the RTSS and genes in general between the cell lines, but that both the IFNα and IFNγ stimulated RTSS overlap poorly with our set of poised RTSS. One can picture a complex situation where similar stimuli invoke different responses depending on the cell line, and also that different stimuli invoke different responses in the same cell line. Thus stimulation by IFNα and IFNγ may simply initiate a response that is different from, or with just minor overlap with, the one represented by our poised RTSS. Second, Pol II changes at RTSS were measured 6 and 30 hours after stimulation with IFNα and IFNγ, which may be long enough for the earliest response to attenuate, and what we observe is actually secondary and further downstream responses. The lower level of active chromatin marks in the unstimulated cells for the regions with the highest change in Pol II after 6h, and the absence of active chromatin marks in regions with the highest change after 30h are indications of this. Overall we find it likely that our set of poised RTSS represent

regulatory elements that rapidly produce transcripts in response to environmental stimuli. Though some of the regulatory elements coincide with annotated gene TSS, many are distal to genes and are likely to produce non-coding transcripts. It is tempting to speculate whether rapid responses initiate at the distal elements and then propagate a signal to nearby genes. Which responses that trigger these elements and how the elements relate to the genes they regulate are interesting candidates for further studies.

S14

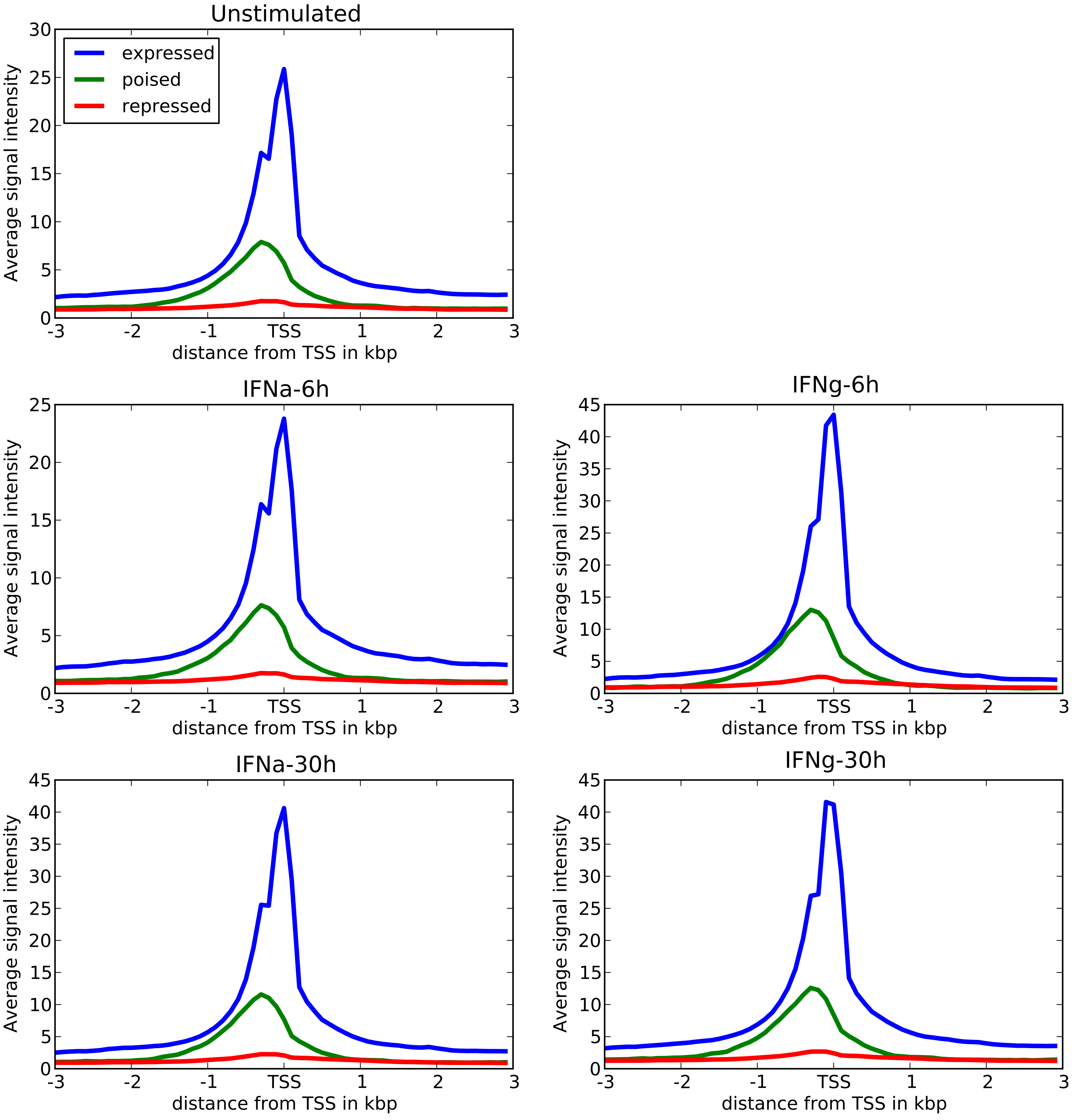

Unstimulated
expressed
poised
repressed
Average signal intensity
0
5
10
15
20
25
30
-3
-2
-1
TSS
1
2
3
distance from TSS in kbp
IFNa-6h
IFNg-6h
IFNa-30h
IFNg-30h
35
40
45

S15

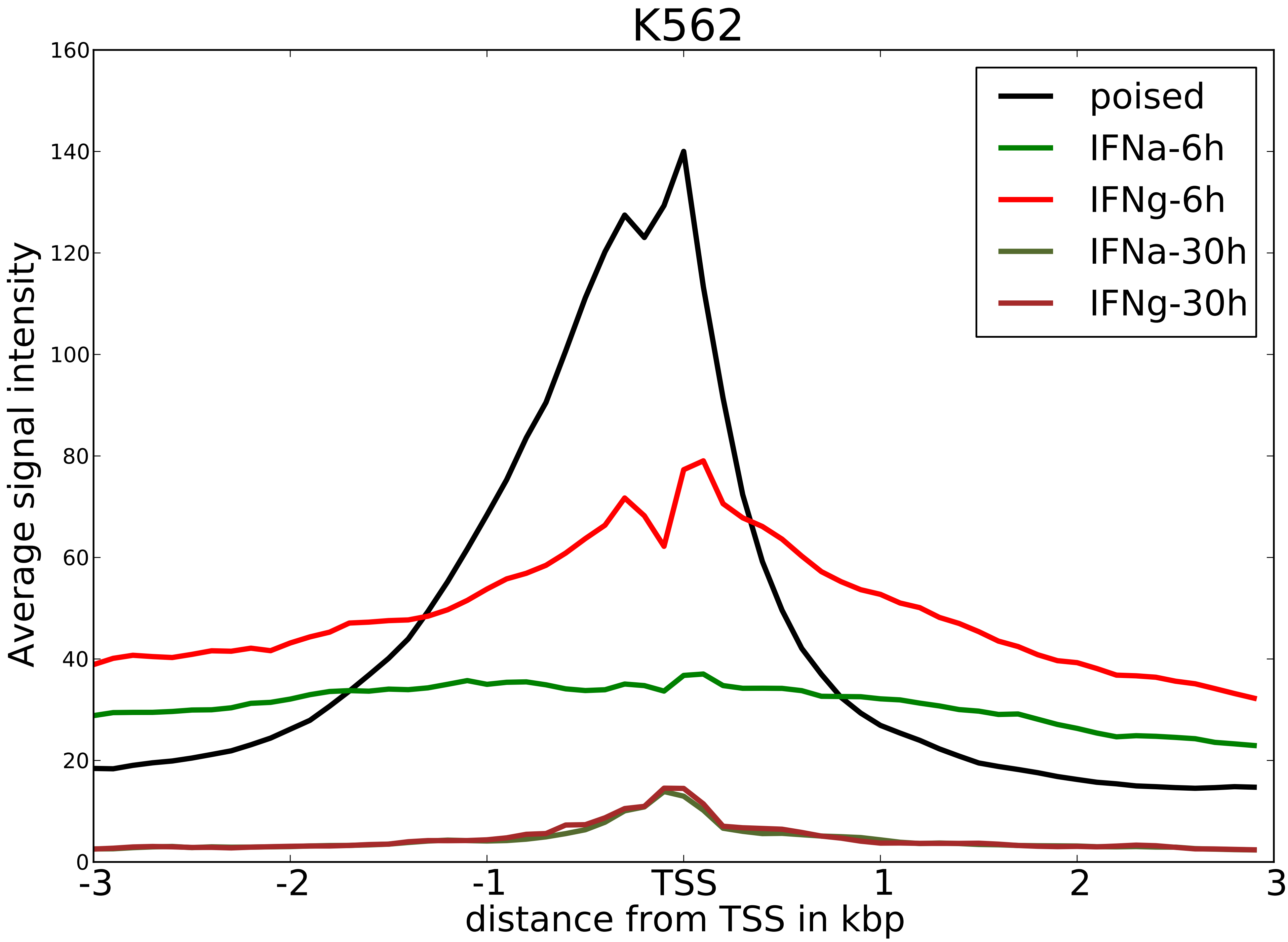
K562
poised
IFNa-6h
IFNg-6h
IFNa-30h
IFNg-30h
Average signal intensity
160
140
120
100
80
60
40
20
0
-3
-2
-1
TSS
1
2
3
distance from TSS in kbp

S16

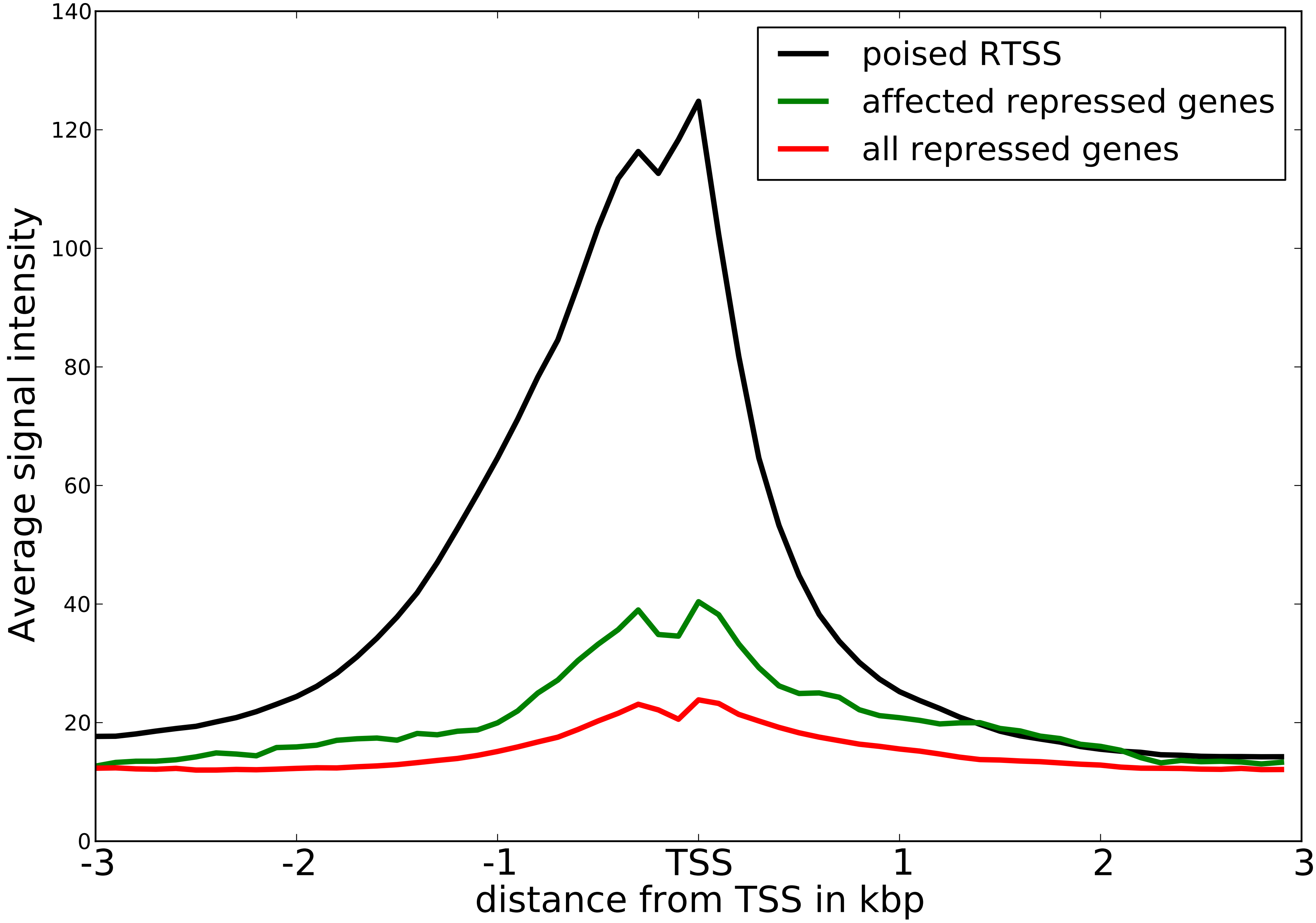

poised RTSS
affected repressed genes
all repressed genes
Average signal intensity
0
20
40
60
80
100
120
140
-3
-2
-1
TSS
1
2
3
distance from TSS in kbp

Data Provider	Cell Line	Biological Assay	Data Type	Dataset Identifier
Broad	K562	Histone Modification	Rawdata	wgEncodeBroadHistoneK562H2azStdAlnRep1.bam
Broad	K562	Histone Modification	Rawdata	wgEncodeBroadHistoneK562H2azStdAlnRep2.bam
Broad	K562	Histone Modification	Rawdata	wgEncodeBroadHistoneK562H3k27acStdAlnRep1.bam
Broad	K562	Histone Modification	Rawdata	wgEncodeBroadHistoneK562H3k27acStdAlnRep2.bam
Broad	K562	Histone Modification	Rawdata	wgEncodeBroadHistoneK562H3k27me3StdAlnRep1.bam
Broad	K562	Histone Modification	Rawdata	wgEncodeBroadHistoneK562H3k27me3StdAlnRep2.bam
Broad	K562	Histone Modification	Rawdata	wgEncodeBroadHistoneK562H3k36me3StdAlnRep1.bam
Broad	K562	Histone Modification	Rawdata	wgEncodeBroadHistoneK562H3k36me3StdAlnRep2.bam
Broad	K562	Histone Modification	Rawdata	wgEncodeBroadHistoneK562H3k4me1StdAlnRep1.bam
Broad	K562	Histone Modification	Rawdata	wgEncodeBroadHistoneK562H3k4me1StdAlnRep2.bam
Broad	K562	Histone Modification	Rawdata	wgEncodeBroadHistoneK562H3k4me2StdAlnRep1.bam
Broad	K562	Histone Modification	Rawdata	wgEncodeBroadHistoneK562H3k4me2StdAlnRep2.bam
Broad	K562	Histone Modification	Rawdata	wgEncodeBroadHistoneK562H3k4me3StdAlnRep1.bam
Broad	K562	Histone Modification	Rawdata	wgEncodeBroadHistoneK562H3k4me3StdAlnRep2.bam
Broad	K562	Histone Modification	Rawdata	wgEncodeBroadHistoneK562H3k79me2StdAlnRep1.bam
Broad	K562	Histone Modification	Rawdata	wgEncodeBroadHistoneK562H3k79me2StdAlnRep2.bam
Broad	K562	Histone Modification	Rawdata	wgEncodeBroadHistoneK562H3k9acStdAlnRep1.bam
Broad	K562	Histone Modification	Rawdata	wgEncodeBroadHistoneK562H3k9acStdAlnRep2.bam
Broad	K562	Histone Modification	Rawdata	wgEncodeBroadHistoneK562H3k9me1StdAlnRep1.bam
Broad	K562	Histone Modification	Rawdata	wgEncodeBroadHistoneK562H3k9me3StdAlnRep1.bam
Broad	K562	Histone Modification	Rawdata	wgEncodeBroadHistoneK562H3k9me3StdAlnRep2.bam
Broad	K562	Histone Modification	Rawdata	wgEncodeBroadHistoneK562H4k20me1StdAlnRep1.bam
Broad	K562	Histone Modification	Rawdata	wgEncodeBroadHistoneK562H4k20me1StdAlnRep2.bam
Broad	Gm12878	Histone Modification	Rawdata	wgEncodeBroadHistoneGm12878H2azStdAlnRep1.bam
Broad	Gm12878	Histone Modification	Rawdata	wgEncodeBroadHistoneGm12878H2azStdAlnRep2.bam
Broad	Gm12878	Histone Modification	Rawdata	wgEncodeBroadHistoneGm12878H3k27acStdAlnRep1.bam
Broad	Gm12878	Histone Modification	Rawdata	wgEncodeBroadHistoneGm12878H3k27acStdAlnRep2.bam
Broad	Gm12878	Histone Modification	Rawdata	wgEncodeBroadHistoneGm12878H3k27me3StdAlnRep1.bam
Broad	Gm12878	Histone Modification	Rawdata	wgEncodeBroadHistoneGm12878H3k27me3StdAlnRep2.bam
Broad	Gm12878	Histone Modification	Rawdata	wgEncodeBroadHistoneGm12878H3k36me3StdAlnRep1.bam
Broad	Gm12878	Histone Modification	Rawdata	wgEncodeBroadHistoneGm12878H3k36me3StdAlnRep2.bam
Broad	Gm12878	Histone Modification	Rawdata	wgEncodeBroadHistoneGm12878H3k4me1StdAlnRep1.bam
Broad	Gm12878	Histone Modification	Rawdata	wgEncodeBroadHistoneGm12878H3k4me1StdAlnRep2.bam
Broad	Gm12878	Histone Modification	Rawdata	wgEncodeBroadHistoneGm12878H3k4me2StdAlnRep1.bam
Broad	Gm12878	Histone Modification	Rawdata	wgEncodeBroadHistoneGm12878H3k4me2StdAlnRep2.bam
Broad	Gm12878	Histone Modification	Rawdata	wgEncodeBroadHistoneGm12878H3k4me3StdAlnRep1.bam
Broad	Gm12878	Histone Modification	Rawdata	wgEncodeBroadHistoneGm12878H3k4me3StdAlnRep2.bam
Broad	Gm12878	Histone Modification	Rawdata	wgEncodeBroadHistoneGm12878H3k79me2StdAlnRep1.bam
Broad	Gm12878	Histone Modification	Rawdata	wgEncodeBroadHistoneGm12878H3k79me2StdAlnRep2.bam
Broad	Gm12878	Histone Modification	Rawdata	wgEncodeBroadHistoneGm12878H3k9acStdAlnRep1.bam
Broad	Gm12878	Histone Modification	Rawdata	wgEncodeBroadHistoneGm12878H3k9acStdAlnRep2.bam
Broad	Gm12878	Histone Modification	Rawdata	wgEncodeBroadHistoneGm12878H3k9me3StdAlnRep1.bam
Broad	Gm12878	Histone Modification	Rawdata	wgEncodeBroadHistoneGm12878H3k9me3StdAlnRep2.bam
Broad	Gm12878	Histone Modification	Rawdata	wgEncodeBroadHistoneGm12878H4k20me1StdAlnRep1.bam
Broad	Gm12878	Histone Modification	Rawdata	wgEncodeBroadHistoneGm12878H4k20me1StdAlnRep2.bam
Broad	Helas3	Histone Modification	Rawdata	wgEncodeBroadHistoneHelas3H2azAlnRep1.bam
Broad	Helas3	Histone Modification	Rawdata	wgEncodeBroadHistoneHelas3H2azAlnRep2.bam
Broad	Helas3	Histone Modification	Rawdata	wgEncodeBroadHistoneHelas3H3k04me1StdAlnRep1.bam
Broad	Helas3	Histone Modification	Rawdata	wgEncodeBroadHistoneHelas3H3k04me1StdAlnRep2.bam
Broad	Helas3	Histone Modification	Rawdata	wgEncodeBroadHistoneHelas3H3k09me3AlnRep1.bam
Broad	Helas3	Histone Modification	Rawdata	wgEncodeBroadHistoneHelas3H3k09me3AlnRep2.bam
Broad	Helas3	Histone Modification	Rawdata	wgEncodeBroadHistoneHelas3H3k27acStdAlnRep1.bam
Broad	Helas3	Histone Modification	Rawdata	wgEncodeBroadHistoneHelas3H3k27acStdAlnRep2.bam
Broad	Helas3	Histone Modification	Rawdata	wgEncodeBroadHistoneHelas3H3k27me3StdAlnRep1.bam
Broad	Helas3	Histone Modification	Rawdata	wgEncodeBroadHistoneHelas3H3k27me3StdAlnRep2.bam
Broad	Helas3	Histone Modification	Rawdata	wgEncodeBroadHistoneHelas3H3k36me3StdAlnRep1.bam
Broad	Helas3	Histone Modification	Rawdata	wgEncodeBroadHistoneHelas3H3k36me3StdAlnRep2.bam
Broad	Helas3	Histone Modification	Rawdata	wgEncodeBroadHistoneHelas3H3k4me2StdAlnRep1.bam
Broad	Helas3	Histone Modification	Rawdata	wgEncodeBroadHistoneHelas3H3k4me2StdAlnRep2.bam
Broad	Helas3	Histone Modification	Rawdata	wgEncodeBroadHistoneHelas3H3k4me3StdAlnRep1.bam

Broad	Helas3	Histone Modification	Rawdata	wgEncodeBroadHistoneHelas3H3k4me3StdAlnRep2.bam
Broad	Helas3	Histone Modification	Rawdata	wgEncodeBroadHistoneHelas3H3k79me2StdAlnRep1.bam
Broad	Helas3	Histone Modification	Rawdata	wgEncodeBroadHistoneHelas3H3k79me2StdAlnRep2.bam
Broad	Helas3	Histone Modification	Rawdata	wgEncodeBroadHistoneHelas3H3k9acStdAlnRep1.bam
Broad	Helas3	Histone Modification	Rawdata	wgEncodeBroadHistoneHelas3H3k9acStdAlnRep2.bam
Broad	Helas3	Histone Modification	Rawdata	wgEncodeBroadHistoneHelas3H4k20me1StdAlnRep1.bam
Broad	Helas3	Histone Modification	Rawdata	wgEncodeBroadHistoneHelas3H4k20me1StdAlnRep2.bam
Broad	Hepg2	Histone Modification	Rawdata	wgEncodeBroadHistoneHepg2H2azStdAlnRep1.bam
Broad	Hepg2	Histone Modification	Rawdata	wgEncodeBroadHistoneHepg2H2azStdAlnRep2.bam
Broad	Hepg2	Histone Modification	Rawdata	wgEncodeBroadHistoneHepg2H3k04me1StdAlnRep1.bam
Broad	Hepg2	Histone Modification	Rawdata	wgEncodeBroadHistoneHepg2H3k04me1StdAlnRep2.bam
Broad	Hepg2	Histone Modification	Rawdata	wgEncodeBroadHistoneHepg2H3k09me3AlnRep1.bam
Broad	Hepg2	Histone Modification	Rawdata	wgEncodeBroadHistoneHepg2H3k09me3AlnRep2.bam
Broad	Hepg2	Histone Modification	Rawdata	wgEncodeBroadHistoneHepg2H3k27acStdAlnRep1.bam
Broad	Hepg2	Histone Modification	Rawdata	wgEncodeBroadHistoneHepg2H3k27acStdAlnRep2.bam
Broad	Hepg2	Histone Modification	Rawdata	wgEncodeBroadHistoneHepg2H3k27me3StdAlnRep1.bam
Broad	Hepg2	Histone Modification	Rawdata	wgEncodeBroadHistoneHepg2H3k27me3StdAlnRep2.bam
Broad	Hepg2	Histone Modification	Rawdata	wgEncodeBroadHistoneHepg2H3k36me3StdAlnRep1.bam
Broad	Hepg2	Histone Modification	Rawdata	wgEncodeBroadHistoneHepg2H3k36me3StdAlnRep2.bam
Broad	Hepg2	Histone Modification	Rawdata	wgEncodeBroadHistoneHepg2H3k4me2StdAlnRep1.bam
Broad	Hepg2	Histone Modification	Rawdata	wgEncodeBroadHistoneHepg2H3k4me2StdAlnRep2.bam
Broad	Hepg2	Histone Modification	Rawdata	wgEncodeBroadHistoneHepg2H3k4me3StdAlnRep1.bam
Broad	Hepg2	Histone Modification	Rawdata	wgEncodeBroadHistoneHepg2H3k4me3StdAlnRep2.bam
Broad	Hepg2	Histone Modification	Rawdata	wgEncodeBroadHistoneHepg2H3k79me2StdAlnRep1.bam
Broad	Hepg2	Histone Modification	Rawdata	wgEncodeBroadHistoneHepg2H3k79me2StdAlnRep2.bam
Broad	Hepg2	Histone Modification	Rawdata	wgEncodeBroadHistoneHepg2H3k9acStdAlnRep1.bam
Broad	Hepg2	Histone Modification	Rawdata	wgEncodeBroadHistoneHepg2H3k9acStdAlnRep2.bam
Broad	Hepg2	Histone Modification	Rawdata	wgEncodeBroadHistoneHepg2H4k20me1StdAlnRep1.bam
Broad	Hepg2	Histone Modification	Rawdata	wgEncodeBroadHistoneHepg2H4k20me1StdAlnRep2.bam
UW	Hepg2	H3K27me3	Rawdata	wgEncodeUwHistoneHepg2H3k27me3StdAlnRep1.bam
UW	Hepg2	H3K27me3	Rawdata	wgEncodeUwHistoneHepg2H3k27me3StdAlnRep2.bam
Duke	Gm12878	DNase HS	Rawdata	wgEncodeOpenChromDnaseGm12878AlnRep1.bam
Duke	Gm12878	DNase HS	Rawdata	wgEncodeOpenChromDnaseGm12878AlnRep2.bam
Duke	Helas3	DNase HS	Rawdata	wgEncodeOpenChromDnaseHelas3AlnRep1.bam
Duke	Helas3	DNase HS	Rawdata	wgEncodeOpenChromDnaseHelas3AlnRep2.bam
Duke	Hepg2	DNase HS	Rawdata	wgEncodeOpenChromDnaseHepg2AlnRep1.bam
Duke	Hepg2	DNase HS	Rawdata	wgEncodeOpenChromDnaseHepg2AlnRep2.bam
Duke	K562	DNase HS	Rawdata	wgEncodeOpenChromDnaseK562AlnRep1.bam
Duke	K562	DNase HS	Rawdata	wgEncodeOpenChromDnaseK562AlnRep2.bam
Broad	K562	Histone Modification	Enrichment Region	wgEncodeBroadHistoneK562H2azStdPk.broadPeak
Broad	K562	Histone Modification	Enrichment Region	wgEncodeBroadHistoneK562H3k27acStdPk.broadPeak
Broad	K562	Histone Modification	Enrichment Region	wgEncodeBroadHistoneK562H3k27me3StdPk.broadPeak
Broad	K562	Histone Modification	Enrichment Region	wgEncodeBroadHistoneK562H3k36me3StdPk.broadPeak
Broad	K562	Histone Modification	Enrichment Region	wgEncodeBroadHistoneK562H3k4me1StdPk.broadPeak
Broad	K562	Histone Modification	Enrichment Region	wgEncodeBroadHistoneK562H3k4me2StdPk.broadPeak
Broad	K562	Histone Modification	Enrichment Region	wgEncodeBroadHistoneK562H3k4me3StdPk.broadPeak
Broad	K562	Histone Modification	Enrichment Region	wgEncodeBroadHistoneK562H3k79me2StdPk.broadPeak
Broad	K562	Histone Modification	Enrichment Region	wgEncodeBroadHistoneK562H3k9acStdPk.broadPeak
Broad	K562	Histone Modification	Enrichment Region	wgEncodeBroadHistoneK562H3k9me1StdPk.broadPeak
Broad	K562	Histone Modification	Enrichment Region	wgEncodeBroadHistoneK562H3k9me3StdPk.broadPeak
Broad	K562	Histone Modification	Enrichment Region	wgEncodeBroadHistoneK562H4k20me1StdPk.broadPeak
Broad	Gm12878	Histone Modification	Enrichment Region	wgEncodeBroadHistoneGm12878H2azStdPk.broadPeak
Broad	Gm12878	Histone Modification	Enrichment Region	wgEncodeBroadHistoneGm12878H3k27acStdPk.broadPeak
Broad	Gm12878	Histone Modification	Enrichment Region	wgEncodeBroadHistoneGm12878H3k27me3StdPk.broadPeak
Broad	Gm12878	Histone Modification	Enrichment Region	wgEncodeBroadHistoneGm12878H3k36me3StdPk.broadPeak
Broad	Gm12878	Histone Modification	Enrichment Region	wgEncodeBroadHistoneGm12878H3k4me1StdPk.broadPeak
Broad	Gm12878	Histone Modification	Enrichment Region	wgEncodeBroadHistoneGm12878H3k4me2StdPk.broadPeak
Broad	Gm12878	Histone Modification	Enrichment Region	wgEncodeBroadHistoneGm12878H3k4me3StdPk.broadPeak
Broad	Gm12878	Histone Modification	Enrichment Region	wgEncodeBroadHistoneGm12878H3k79me2StdPk.broadPeak
Broad	Gm12878	Histone Modification	Enrichment Region	wgEncodeBroadHistoneGm12878H3k9acStdPk.broadPeak
Broad	Gm12878	Histone Modification	Enrichment Region	wgEncodeBroadHistoneGm12878H3k9me3StdPk.broadPeak

Broad	Gm12878	Histone Modification	Enrichment Region	wgEncodeBroadHistoneGm12878H4k20me1StdPk.broadPeak
Broad	Helas3	Histone Modification	Enrichment Region	wgEncodeBroadHistoneHelas3H2azPk.broadPeak
Broad	Helas3	Histone Modification	Enrichment Region	wgEncodeBroadHistoneHelas3H3k04me1StdPk.broadPeak
Broad	Helas3	Histone Modification	Enrichment Region	wgEncodeBroadHistoneHelas3H3k09me3Pk.broadPeak
Broad	Helas3	Histone Modification	Enrichment Region	wgEncodeBroadHistoneHelas3H3k27acStdPk.broadPeak
Broad	Helas3	Histone Modification	Enrichment Region	wgEncodeBroadHistoneHelas3H3k27me3StdPk.broadPeak
Broad	Helas3	Histone Modification	Enrichment Region	wgEncodeBroadHistoneHelas3H3k36me3StdPk.broadPeak
Broad	Helas3	Histone Modification	Enrichment Region	wgEncodeBroadHistoneHelas3H3k4me2StdPk.broadPeak
Broad	Helas3	Histone Modification	Enrichment Region	wgEncodeBroadHistoneHelas3H3k4me3StdPk.broadPeak
Broad	Helas3	Histone Modification	Enrichment Region	wgEncodeBroadHistoneHelas3H3k79me2StdPk.broadPeak
Broad	Helas3	Histone Modification	Enrichment Region	wgEncodeBroadHistoneHelas3H3k9acStdPk.broadPeak
Broad	Helas3	Histone Modification	Enrichment Region	wgEncodeBroadHistoneHelas3H4k20me1StdPk.broadPeak
Broad	Hepg2	Histone Modification	Enrichment Region	wgEncodeBroadHistoneHepg2H2azStdPk.broadPeak
Broad	Hepg2	Histone Modification	Enrichment Region	wgEncodeBroadHistoneHepg2H3k04me1StdPk.broadPeak
Broad	Hepg2	Histone Modification	Enrichment Region	wgEncodeBroadHistoneHepg2H3k09me3Pk.broadPeak
Broad	Hepg2	Histone Modification	Enrichment Region	wgEncodeBroadHistoneHepg2H3k27acStdPk.broadPeak
Broad	Hepg2	Histone Modification	Enrichment Region	wgEncodeBroadHistoneHepg2H3k27me3StdPk.broadPeak
Broad	Hepg2	Histone Modification	Enrichment Region	wgEncodeBroadHistoneHepg2H3k36me3StdPk.broadPeak
Broad	Hepg2	Histone Modification	Enrichment Region	wgEncodeBroadHistoneHepg2H3k4me2StdPk.broadPeak
Broad	Hepg2	Histone Modification	Enrichment Region	wgEncodeBroadHistoneHepg2H3k4me3StdPk.broadPeak
Broad	Hepg2	Histone Modification	Enrichment Region	wgEncodeBroadHistoneHepg2H3k79me2StdPk.broadPeak
Broad	Hepg2	Histone Modification	Enrichment Region	wgEncodeBroadHistoneHepg2H3k9acStdPk.broadPeak
Broad	Hepg2	Histone Modification	Enrichment Region	wgEncodeBroadHistoneHepg2H4k20me1StdPk.broadPeak
Duke	K562	DNase HS	Enrichment Region	wgEncodeOpenChromDnaseK562Pk.narrowPeak
Duke	Gm12878	DNase HS	Enrichment Region	wgEncodeOpenChromDnaseGm12878Pk.narrowPeak
Duke	Helas3	DNase HS	Enrichment Region	wgEncodeOpenChromDnaseHelas3Pk.narrowPeak
Duke	Hepg2	DNase HS	Enrichment Region	wgEncodeOpenChromDnaseHepg2Pk.narrowPeak
SYDH	Gm12878	Polymerase 2	Rawdata	wgEncodeSydhTfbsGm12878Pol2StdAlnRep1.bam
SYDH	Gm12878	Polymerase 2	Rawdata	wgEncodeSydhTfbsGm12878Pol2StdAlnRep2.bam
SYDH	Helas3	Polymerase 2	Rawdata	wgEncodeSydhTfbsHelas3Pol2StdAlnRep1.bam
SYDH	Helas3	Polymerase 2	Rawdata	wgEncodeSydhTfbsHelas3Pol2StdAlnRep2.bam
SYDH	Hepg2	Polymerase 2	Rawdata	wgEncodeSydhTfbsHepg2Pol2IggrabAlnRep1.bam
SYDH	Hepg2	Polymerase 2	Rawdata	wgEncodeSydhTfbsHepg2Pol2IggrabAlnRep2.bam
SYDH	K562	Polymerase 2	Rawdata	wgEncodeSydhTfbsK562Pol2StdAlnRep1.bam
SYDH	K562	Polymerase 2	Rawdata	wgEncodeSydhTfbsK562Pol2StdAlnRep2.bam
SYDH	Gm12878	Polymerase 2	Enrichment Region	wgEncodeSydhTfbsGm12878Pol2StdPk.narrowPeak
SYDH	Helas3	Polymerase 2	Enrichment Region	wgEncodeSydhTfbsHelas3Pol2StdPk.narrowPeak
SYDH	Hepg2	Polymerase 2	Enrichment Region	wgEncodeSydhTfbsHepg2Pol2IggrabPk.narrowPeak
SYDH	K562	Polymerase 2	Enrichment Region	wgEncodeSydhTfbsK562Pol2StdPk.narrowPeak
SYDH	K562	IFN-stimulated	Rawdata	wgEncodeSydhTfbsK562Pol2Ifna30StdAlnRep1.bam
SYDH	K562	IFN-stimulated	Rawdata	wgEncodeSydhTfbsK562Pol2Ifna30StdAlnRep2.bam
SYDH	K562	IFN-stimulated	Rawdata	wgEncodeSydhTfbsK562Pol2Ifna6hStdAlnRep1.bam
SYDH	K562	IFN-stimulated	Rawdata	wgEncodeSydhTfbsK562Pol2Ifna6hStdAlnRep2.bam
SYDH	K562	IFN-stimulated	Rawdata	wgEncodeSydhTfbsK562Pol2Ifng30StdAlnRep1.bam
SYDH	K562	IFN-stimulated	Rawdata	wgEncodeSydhTfbsK562Pol2Ifng30StdAlnRep2.bam
SYDH	K562	IFN-stimulated	Rawdata	wgEncodeSydhTfbsK562Pol2Ifng6hStdAlnRep1.bam
SYDH	K562	IFN-stimulated	Rawdata	wgEncodeSydhTfbsK562Pol2Ifng6hStdAlnRep2.bam
SYDH	K562	IFN-stimulated	Enrichment Region	wgEncodeSydhTfbsK562Pol2Ifna30StdPk.narrowPeak
SYDH	K562	IFN-stimulated	Enrichment Region	wgEncodeSydhTfbsK562Pol2Ifna6hStdPk.narrowPeak
SYDH	K562	IFN-stimulated	Enrichment Region	wgEncodeSydhTfbsK562Pol2Ifng30StdPk.narrowPeak
SYDH	K562	IFN-stimulated	Enrichment Region	wgEncodeSydhTfbsK562Pol2Ifng6hStdPk.narrowPeak
SYDH	Gm12878	Nucleosome	bigWig track	wgEncodeSydhNsomeGm12878Sig.bigWig
SYDH	K562	Nucleosome	bigWig track	wgEncodeSydhNsomeK562Sig.bigWig
Stanford	All	Genomic regions	Unmappable regions	wgEncodeDacMapabilityConsensusExcludable.bed
Duke	All	Genomic regions	Unmappable regions	wgEncodeDukeMapabilityRegionsExcludable.bed

S18

Permutation	Cell type	RTSS track	DNase	H2AZ	H3K27ac	H3K27me3	H3K36me3	H3K4me1	H3K4me2	H3K4me3	H3K79me2	H3K9ac	H3K9me3	H4K20me1
uniform	gm12878	all expressed	9,999E-05	9,999E-05	9,999E-05	1	9,999E-05	9,999E-05	9,999E-05	9,999E-05	9,999E-05	9,999E-05	9,999E-05	9,999E-05
uniform	gm12878	all repressed	9,999E-05	9,999E-05	9,999E-05	9,999E-05	0,045908184	9,999E-05	9,999E-05	9,999E-05	9,999E-05	9,999E-05	9,999E-05	9,999E-05
uniform	gm12878	isolated expressed	9,999E-05	9,999E-05	9,999E-05	1	9,999E-05	9,999E-05	9,999E-05	9,999E-05	9,999E-05	9,999E-05	9,999E-05	9,999E-05
uniform	gm12878	isolated repressed	9,999E-05	9,999E-05	9,999E-05	9,999E-05	0,712871287	9,999E-05	9,999E-05	9,999E-05	0,900990099	9,999E-05	0,012345679	9,999E-05
interseg	gm12878	all expressed	9,999E-05	9,999E-05	9,999E-05	1	9,999E-05	9,999E-05	9,999E-05	9,999E-05	9,999E-05	9,999E-05	9,999E-05	9,999E-05
interseg	gm12878	all repressed	9,999E-05	9,999E-05	9,999E-05	9,999E-05	0,174129353	9,999E-05	9,999E-05	9,999E-05	9,999E-05	9,999E-05	9,999E-05	9,999E-05
interseg	gm12878	isolated expressed	9,999E-05	9,999E-05	9,999E-05	1	9,999E-05	9,999E-05	9,999E-05	9,999E-05	9,999E-05	9,999E-05	9,999E-05	9,999E-05
interseg	gm12878	isolated repressed	9,999E-05	9,999E-05	9,999E-05	9,999E-05	0,673267327	9,999E-05	9,999E-05	9,999E-05	0,920792079	9,999E-05	0,019981835	9,999E-05
interseg	Helas3	all expressed	9,999E-05	9,999E-05	9,999E-05	1	9,999E-05	9,999E-05	9,999E-05	9,999E-05	9,999E-05	9,999E-05	1	9,999E-05
interseg	Helas3	all repressed	9,999E-05	9,999E-05	9,999E-05	9,999E-05	0,174129353	9,999E-05	9,999E-05	9,999E-05	9,999E-05	9,999E-05	1	9,999E-05
interseg	Helas3	isolated expressed	9,999E-05	9,999E-05	9,999E-05	1	9,999E-05	9,999E-05	9,999E-05	9,999E-05	9,999E-05	9,999E-05	1	9,999E-05
interseg	Helas3	isolated repressed	9,999E-05	9,999E-05	9,999E-05	9,999E-05	0,109634551	9,999E-05	9,999E-05	9,999E-05	0,034941764	9,999E-05	0,821782178	9,999E-05
interseg	Hepg2	all expressed	9,999E-05	9,999E-05	9,999E-05	9,999E-05	9,999E-05	9,999E-05	9,999E-05	9,999E-05	9,999E-05	9,999E-05	1	9,999E-05
interseg	Hepg2	all repressed	9,999E-05	9,999E-05	9,999E-05	9,999E-05	0,782178218	9,999E-05	9,999E-05	9,999E-05	9,999E-05	9,999E-05	1	9,999E-05
interseg	Hepg2	isolated expressed	9,999E-05	9,999E-05	9,999E-05	9,99989E-05	9,999E-05	9,999E-05	9,999E-05	9,999E-05	9,999E-05	9,999E-05	1	9,999E-05
interseg	Hepg2	isolated repressed	9,999E-05	9,999E-05	0,035663338	9,999E-05	0,326732673	9,999E-05	9,999E-05	9,999E-05	0,405940594	9,999E-05	1	9,999E-05
interseg	K562	all expressed	9,999E-05	9,999E-05	9,999E-05	1	9,999E-05	9,999E-05	9,999E-05	9,999E-05	9,999E-05	9,999E-05	1	9,999E-05
interseg	K562	all repressed	9,999E-05	9,999E-05	9,999E-05	9,999E-05	9,999E-05	9,999E-05	9,999E-05	9,999E-05	9,999E-05	9,999E-05	1	9,999E-05
interseg	K562	isolated expressed	9,999E-05	9,999E-05	9,999E-05	1	9,999E-05	9,999E-05	9,999E-05	9,999E-05	9,999E-05	9,999E-05	0,841584158	9,999E-05
interseg	K562	isolated repressed	9,999E-05	9,999E-05	9,999E-05	9,999E-05	9,999E-05	9,999E-05	9,999E-05	9,999E-05	0,000405398	9,999E-05	1	9,999E-05
